# Comparative transcriptomics of ice‐crawlers demonstrates cold specialization constrains niche evolution in a relict lineage

**DOI:** 10.1111/eva.13120

**Published:** 2020-09-11

**Authors:** Sean D. Schoville, Sabrina Simon, Ming Bai, Zachary Beethem, Roman Y. Dudko, Monika J. B. Eberhard, Paul B. Frandsen, Simon C. Küpper, Ryuichiro Machida, Max Verheij, Peter C. Willadsen, Xin Zhou, Benjamin Wipfler

**Affiliations:** ^1^ Department of Entomology University of Wisconsin‐Madison Madison WI USA; ^2^ Biosystematics Group Wageningen University & Research PB Wageningen The Netherlands; ^3^ Key Laboratory of Zoological Systematics and Evolution Institute of Zoology Chinese Academy of Sciences Beijing China; ^4^ Institute of Systematics and Ecology of Animals Siberian Branch of the Russian Academy of Sciences Novosibirsk Russia; ^5^ Tomsk State University Tomsk Russia; ^6^ Zoological Institute and Museum General Zoology and Zoological Systematics University of Greifswald Greifswald Germany; ^7^ Department of Plant & Wildlife Sciences Brigham Young University Provo UT USA; ^8^ Data Science Lab Office of the Chief Information Officer Smithsonian Institution Washington DC U.S.A; ^9^ Sugadaira Research Station Mountain Science Center University of Tsukuba Ueda, Nagano Japan; ^10^ Department of Entomology College of Plant Protection China Agricultural University Beijing China; ^11^ Zoologisches Forschungsmuseum Alexander Koenig Bonn Germany; ^12^ Present address: Department of Biomedical Sciences School of Veterinary Medicine University of Pennsylvania Philadelphia PA USA; ^13^ Present address: Department of Entomology and Plant Pathology North Carolina State University Campus Box 7613 Raleigh NC USA

**Keywords:** adaptation, gene regulation, Grylloblattodea, insect phylogenomics, niche conservatism, protein evolution

## Abstract

Key changes in ecological niche space are often critical to understanding how lineages diversify during adaptive radiations. However, the converse, or understanding why some lineages are depauperate and relictual, is more challenging, as many factors may constrain niche evolution. In the case of the insect order Grylloblattodea, highly conserved thermal breadth is assumed to be closely tied to their relictual status, but has not been formerly tested. Here, we investigate whether evolutionary constraints in the physiological tolerance of temperature can help explain relictualism in this lineage. Using a comparative transcriptomics approach, we investigate gene expression following acute heat and cold stress across members of Grylloblattodea and their sister group, Mantophasmatodea. We additionally examine patterns of protein evolution, to identify candidate genes of positive selection. We demonstrate that cold specialization in Grylloblattodea has been accompanied by the loss of the inducible heat shock response under both acute heat and cold stress. Additionally, there is widespread evidence of selection on protein‐coding genes consistent with evolutionary constraints due to cold specialization. This includes positive selection on genes involved in trehalose transport, metabolic function, mitochondrial function, oxygen reduction, oxidative stress, and protein synthesis. These patterns of molecular adaptation suggest that Grylloblattodea have undergone evolutionary trade‐offs to survive in cold habitats and should be considered highly vulnerable to climate change. Finally, our transcriptomic data provide a robust backbone phylogeny for generic relationships within Grylloblattodea and Mantophasmatodea. Major phylogenetic splits in each group relate to arid conditions driving biogeographical patterns, with support for a sister‐group relationship between North American *Grylloblatta* and Altai‐Sayan *Grylloblattella*, and a range disjunction in Namibia splitting major clades within Mantophasmatodea.

## 1 INTRODUCTION

Species that are considered relicts are by definition old, taxonomically depauperate lineages, where extinction has substantially diminished their former diversity (Grandcolas, Nattier, & Trewick, 2014; Habel, Assmann, Schmitt, & Avise, 2010). This can be difficult to assess when the fossil record is poor, but can be approximated by deeply divergent evolutionary lineages that lack extant diversity relative to sister taxa (although this assumes that speciation rates are not exceptionally low). Notably, this definition of relictualism does not imply conservation of characters or a lack of evolution, and so it remains unclear why relict taxa would experience a disproportionally high extinction rate. Do such relict species exhibit evolutionary constraints that limit ecological divergence? One possibility is that relict species are also niche specialists, which utilize a narrow range of ecological conditions and often exhibit restricted biogeographic distributions (Sexton, Montiel, Shay, Stephens, & Slatyer, 2017). Research on niche evolution remains central to understanding rates of ecological divergence in different regions and taxonomic groups (Ackerly, 2009). While there has been considerable interest in niche‐determining factors that allow lineages to diversify and generate ecological novelty in adaptive radiations (Gavrilets & Losos, 2009; Schluter, 2000), much less attention has been paid to factors that constrain niche space and therefore constrain diversification. Improving our understanding of how relict species interact with their environments and evolve would broaden our understanding of mechanisms linked to extinction rates and whether there are factors that constrain niche evolution. This knowledge, in turn, would aid in efforts to conserve these phylogenetically divergent lineages in the face of habitat loss and environmental change (Ohlemüller et al., 2008; Vane‐Wright, Humphries, & Williams, 1991; Winter, Devictor, & Schweiger, 2013).

Relict species are often used to argue for habitat stability in the regions that they inhabit, as niche conservatism is assumed, but tests for niche evolution are not always undertaken (Grandcolas, 2017). The insect group Grylloblattodea, commonly known as ice‐crawlers and rock‐crawlers, provide a case study of a relict lineage appearing to have had much greater diversity in the past and a broader geographical distribution (Schoville & Kim, 2011; Vrsansky, Storozhenko, Labandeira, & Ihraingova, 2001). Grylloblattodea are also considered niche specialists, as they are only found in cool and humid microhabitats in mountainous terrain in eastern Asia and western North America (Schoville, 2014; Schoville, Slatyer, Bergdahl, & Valdez, 2015). Ice‐crawlers, like other insects, are poikilotherms that depend on environmental conditions to regulate body temperatures, but they are noteworthy among insects for exhibiting cold specialization. The genus *Grylloblatta* forages nocturnally on snowfields and prefers ambient temperatures close to 0°C (Mills & Pepper, 1937). The most heat tolerant Grylloblattodeans, members of *Galloisiana* in Japan, live in cool, rocky soil and prefer only slightly warmer ambient temperatures (4°C–10°C; Nagashima, Ando, & Fukushima, 1982). In contrast, other Polyneopteran insects prefer much warmer temperatures, even ambient temperatures >30°C (Forsman, 2000; Harris, McQuillan, & Hughes, 2013). The closest relatives to Grylloblattodea, their sister taxon Mantophasmatodea (known as the heelwalkers or gladiators), are found in sparsely vegetated regions of South Africa that are on average warmer, although temperatures can vary widely within a single day, from freezing to 35°C (Roth, Molina, & Predel, 2014). Males of the mantophasmid *Karoophasma biedouwense* prefer temperatures of 25°C for sexual recruitment of females, and cease activity below 15°C and above 30°C (Eberhard, Metze, & Küpper, 2019). In contrast, North American ice‐crawlers are freeze‐avoidant (surviving up to their supercooling point, −3.9 ± 1.0°C) and stenothermal (Edwards, 1982; Schoville et al., 2015; Sinclair, Alvarado, & Ferguson, 2015), with strong conservation in both thermal tolerance breadth (31°C) and critical thermal limits (−4.0 to 27.0°C) based on laboratory experiments. This implies evolutionary constraints exist for thermal niche evolution, and indeed, some species of *Galloisiana* and *Namkungia* have shifted to subterranean habitats and become eyeless where they have persisted in warm climatic regions of East Asia (Schoville & Kim, 2011).

Insect survival strategies at freezing temperatures may relate to environmental predictability: Freeze tolerance (survival of internal ice formation) predominates in unpredictable environments, whereas freeze avoidance (ice formation is lethal) is more common in predictable environments (Sinclair, Addo‐Bediako, & Chown, 2003). The constrained thermal breadth and foraging behavior of Grylloblattodea suggest freeze avoidance (Schoville et al., 2015), and this may have evolved due to the energetic requirements of living in consistently cold habitats. Organisms in cold habitats must adjust energy metabolism, ion homeostasis, neuromuscular control, and protein function to survive (Hayward, Manso, & Cossins, 2014). These adjustments may lead to irreversible evolutionary trade‐offs. For example, in stenothermal Antarctic fish, cold specialization has led to a loss of inducible heat shock responses, and upregulation of pathways involved in protein degradation (especially ubiquitination and proteosomal degradation), antioxidant response, protein synthesis, and metabolism (Hofmann, Buckley, Airaksinen, Keen, & Somero, 2000; Logan & Buckley, 2015). Upon exposure to heat, these fish experience elevated oxidative stress and inflammation that constrain performance in warm or highly variable thermal environments (Logan & Buckley, 2015). However, among cold‐specialized insects, research has shown that there are a broad array of strategies to deal with cold environmental conditions (Hayward et al., 2014; Storey & Storey, 2012). Most of these insects rely on inducible mechanisms of gene regulation to cope with cold conditions, which can be reversed under warmer conditions. It is unclear how cold specialization has evolved in Grylloblattodea or whether the accompanying physiological changes constrain niche evolution.

Cellular responses to environmental stressors can shed light on the physiological mechanisms that might constrain evolutionary change in different organisms (Tomanek, 2008). In particular, changes in gene expression following exposure to thermal stress provide insight into gene regulatory pathways that underlie temperature tolerance, although the magnitude and timing of gene expression responses can depend on stress exposure and gene expression is not always strongly correlated with protein abundance and activity (Evans, 2015; Schwanhäusser et al., 2011). Here, we examine whether acute thermal stress activates gene expression differentially in members of Grylloblattodea. We also examine genomic divergence patterns for evidence of positive selection in protein‐coding genes related to cold specialization. Finally, these data provide an opportunity to resolve uncertainty in the deeper phylogeny of Grylloblattodea (Jarvis & Whiting, 2006; Schoville, Uchifune, & Machida, 2013), particularly in the sister‐group relationship among Asian and North American genera. Using a comparative approach, we investigate cold specialization and physiological niche divergence in Grylloblattodea relative to their sister‐group Mantophasmatodea. Grylloblattodea and Mantophasmatodea diverged more than 150 million years ago (Eberhard, Schoville, & Klass, 2018; Misof et al., 2014), and, despite this long period of evolutionary history, they remain the two smallest insect orders (33 and 25 currently described extant species, respectively). Both lineages had formerly widespread fossil ancestors, and, as a result, they are both considered relictual orders (Roth et al., 2014; Schoville & Kim, 2011). Here, however, we focus on the evolutionary consequences of relictualism in Grylloblattodea, to closely examine the role of niche evolution in this climate specialized lineage.

## 2 MATERIALS AND METHODS

### 2.1 Taxon sampling and thermal stress experiments

A total of 84 specimens (Table 1) were collected for this project during 2012–2016 by the authors, and with the assistance of international collaborators, under a variety of permits (California Department of Fish and Wildlife #006977, Lassen Volcanic National Park #LAVO‐01880, Lava Beds National Monument #LABE‐00053, Mt. Rainier National Park #MORA‐00185, North Cascade National Park #NOCA‐00005, Yosemite National Park #YOSE‐00234, Sequoia and Kings National Park #SEKI‐00091, Washington State Department of Fish and Wildlife #14‐116, Schoville 154‐184, and Western Cape Province Cape Nature, South Africa #0056‐AAA041‐00139, Eberhard). Some samples were collected as part of the 1KITE project (http://www.1kite.org/; Table S1), and transcriptomes of three species used in this study (*Grylloblatta bifratrilecta*, *Galloisiana* sp. "Joshin'etsu‐Kogen Highlands," and *Tanzaniophamsa* sp. nov.) were published in Misof et al. (2014).

**TABLE 1:** Taxon sampling, RNA sequencing treatments, and resulting genetic datasets. Previously published data are noted with an asterisk (*). See Table S2 for additional detail

Grylloblattodea	Locality	Treatment (sample size)	# Orthologs Orthograph Dataset 1	# Orthologs HAMSTR Dataset 2	NCBI SRA biosample
*Grylloblattina djakonovi*	Mnogoudobnoye, Primorsky Krai, Russia	1KITE (1)	2,345	1,528	SAMN04005167
*Grylloblattella pravdini*	Yambash Mtn., Russia	1KITE (1)	2,086	1,448	SAMN04005166
	Evrechela Mountain, Russia	Control (3) Heat (3) Cold (3)	2,627	N/A	SAMN14584010 to SAMN14584018
*Galloisiana sinensis*	Changbaishan, Jilin Prov., China	1KITE (1)	2,364	1,517	SAMN04005163
*Galloisiana yezoensis*	Yubari, Hokkaido, Japan	Control (2) Heat (2) Cold (2)	2,593	1,420	SAMN14584019 to SAMN14584024
*Galloisiana* sp. "Kanto Mountains"	Kami‐oshima, Kanagawa, Honshu, Japan	1KITE (1)	2,449	1,535	SAMN04005162
*Galloisiana* sp. "Joshin'etsu‐Kogen Highlands"	Sugadaira Montane Research Center, Nagano, Honshu, Japan	1KITE* (1)	2,736	1,552	SAMN02047172
*Grylloblatta bifratrilecta*	Sonora Pass, CA, U.S.A.	1KITE* (1) Control (2) Heat (2) Cold (2)	2,355	1,523	SAMN02047192, SAMN14584025 to SAMN14584030
*Grylloblatta* sp. "Lillburn Cave"	Redwood Creek, CA, U.S.A.	Control (2) Heat (2) Cold (2)	2,692	1,454	SAMN14584031 to SAMN14584036
*Grylloblatta* sp. "Sierra Buttes"	Sierra Buttes, CA, U.S.A.	Control (2) Heat (2) Cold (1)	2,612	1,405	SAMN14584037 to SAMN14584041
*Grylloblatta chandleri*	Lassen Peak, CA, U.S.A.	Control (1)	1,904	1,093	SAMN14584042
*Grylloblatta gurneyi*	Lava Beds National Monument, CA, U.S.A.	Control (1) Heat (1)	2,149	1,223	SAMN14584043 SAMN14584044
	Mt. Shasta, CA, U.S.A.	Control (1) Heat (2)	2,322	1,275	SAMN14584045 to SAMN14584047
*Grylloblatta marmoreus*	Marble Mountains, CA, U.S.A.	Control (2) Heat (1) Cold (1)	2,581	1,395	SAMN14584048 to SAMN14584051
*Grylloblatta* sp. "Trinity Alps"	Thompson Peak, CA, U.S.A.	Control (2)	1,617	984	SAMN14584052 SAMN14584053
*Grylloblatta campodeiformis occidentalis*	Table Mountain, Mt. Baker N.F., WA, U.S.A.	Control (1)	1,914	1,109	SAMN14584054
*Grylloblatta* sp. "North Cascades"	Paradise, Mt. Rainier, WA, U.S.A.	Control (1) Heat (1) Cold (2)	2,525	1,379	SAMN14584055 to SAMN14584058
*Grylloblatta* sp. "North Cascades"	Whitechuck Mountain, WA, U.S.A.	Control (2) Heat (2) Cold (2)	2,650	1,457	SAMN14584059 to SAMN14584064
Mantophasmatodea					
Austrophasmatidae sp. 1 (36)	Vanrhynsdorp, West Coast District, Western Cape Prov., South Africa	1KITE (1)	1,992	877	SAMN04005129
Austrophasmatidae sp. 2 (72)	Richtersfeld, Northern Cape Prov., South Africa	1KITE (1)	2,217	773	SAMN04005128
*Karoophasma biedouwense*	Clanwilliam Dam, Western Cape Province, South Africa	Control (5) Heat (5) Cold (5)	3,191	1,575	SAMN14584065 to SAMN14584079
*Mantophasma* sp. 1	Waterberg‐Plateau, Waterberg, Namibia	1KITE (1)	2,171	1,423	SAMN03339343
*Mantophasma* sp. 2 (109)	Spitzkoppe, Erongo, Namibia	1KITE (1)	2,146	938	SAMN04005187
*Pachyphasma brandbergense*	Brandberg Plateau, Erongo, Namibia	1KITE (1)	1,612	913	SAMN04005210
*Striatophasma naukluftense*	Naukluft Mountains, Hardap, Namibia	1KITE (1)	2,258	1,070	SAMN04005246
*Tanzaniophasma* sp. nov.	Dedza Mountains, Malawi	1KITE* (1)	2,552	1,070	SAMN02047176
*Tyrannophasma gladiator*	Brandberg Plateau, Erongo, Namibia	1KITE (1)	2,398	1,145	SAMN04005258

Sampling comprises 17 Grylloblattodea taxa (Table 1), including the Asian genera *Grylloblattina*, *Grylloblattella*, and *Galloisiana*, and nine species from the North American genus *Grylloblatta*. Both alpine and cave species of *Grylloblatta* were collected, and for two species, multiple populations were sampled, including a cave and alpine population of *Grylloblatta gurneyi*. Nine species of Mantophasmatodea were collected (Table 1), representing the six genera *Karoophasma*, *Mantophasma*, *Pachyphasma*, *Striatophasma*, *Tanzaniophasma*, and *Tyrannophasma*, as well as two undescribed taxa from the family Austrophasmatidae. Specimens were collected in RNAlater (Sigma‐Aldrich) and manually homogenized for all body tissues, transported at room temperature, and stored frozen (at −80°C) for up to 6 months prior to sequencing. All samples have been deposited at the National Center for Biotechnology Information (NCBI) in the in the Short‐Read Archive (see Table 1 and Table S1 for accessions; additional data, e.g., sex, collection date, is provided on NCBI or in Table S2).

A subset of 66 samples were exposed to either an acute heat stress treatment, cold stress treatment, or control treatment (ambient conditions) to characterize thermal stress responses (Table 1; Table S2). Stressful conditions were chosen to be near estimates of critical thermal minima and maxima (Schoville et al., 2015), and to characterize rapid responses to extreme temperature exposure. Across published studies, the recorded intervals of mRNA levels following stress vary across experiments, yet it is well known that initiation of stress response pathways occurs within minutes (De Nadal, Ammerer, & Posas, 2011) and changes in mRNA levels are readily detectable 10–30 min postexposure in a wide range of organisms, from yeast (Gasch et al., 2000) to fruit flies (Udaka, Ueda, & Goto, 2010). Here, we chose to focus on an acute 30 min exposure to temperature stress (details of these treatments, including temperature settings, can be found in Table S2). Briefly, specimens were exposed directly to a test temperature, which varied among taxa to avoid inducing mortality. For ice‐crawlers, −5°C was used for a cold treatment, except for *Galloisiana yezoensis*, which was 2.5°C. Under the heat treatment, 20^o^C was used for most taxa, except *Grylloblattella pravdini* received 25°C and *G. yezoensis* received 35°C. For *K. biedouwense*, the cold treatment occurred at 0°C, and the heat treatment occurred at 40°C. Samples were directly preserved in RNA later at the end of the temperature exposure. With the exception of one adult male, all mantophasmatodean samples were adult females. For Grylloblattodea taxa, adult females were preferentially chosen for these experiments, but, in some cases, males were used, or late stage nymphs (for which sex could not be determined), and in one case four 2nd‐3rd instar nymphs were pooled as a single sample to obtain sufficient RNA. Due to the rarity of grylloblattodean specimens, sample sizes were small (ranging from two to nine individuals, see Table 1).

### 2.2 RNA extraction and library preparation

For samples from the 1KITE project, RNA extraction, cDNA library preparation, and transcriptome sequencing (Illumina HiSeq 2000 platform with 150 bp paired‐end (PE) reads and a 250 bp insert size) were conducted at the Beijing Genomics Institute (BGI) Shenzhen and are described in detail by Misof et al. (2014). For the remaining samples, manually homogenized tissues were further homogenized using a Kinematica Polytron PT10‐35 and total RNA was extracted using Trizol reagent (Life Technologies), according to the manufacturer's protocol. RNA was assessed for concentration and size quality using a 2100 Bioanalyzer (Agilent Technologies, Inc.) Total RNA nano chip. Sequencing libraries were generated by the UW‐Madison Biotechnology Center using mRNA TruSeq kits (Illumina Corp.), as 50 bp PE libraries with a 130 bp insert size. RNAseq libraries were then sequenced on the Illumina HiSeq 2500 platform.

### 2.3 Transcriptome assemblies and annotation

Using the short‐read RNAseq data, transcriptome assemblies were generated de novo for each taxon. Samples from the 1KITE project were trimmed and assembled using soapdenovo‐trans with a 31kmer size (Xie et al., 2014), following protocols described in detail by Peters et al. (2017). For all other samples, raw reads for each species or population were first assessed for quality using fastqc v.0.10.1 (Babraham Bioinformatics, 2011). Based on these results, we used trimmomatic v0.32 (Bolger, Lohse, & Usadel, 2014) to trim the raw reads, removing three base pairs from the head of each read, and any leading and trailing bases with quality scores less than 34. The program trinity v2.0.3 (Haas et al., 2013) was used to generate de novo transcriptome assemblies for each species from all pooled samples, using default settings. Transcriptomes were analyzed for vector contamination using the program seqclean (https://sourceforge.net/projects/seqclean/) and the NCBI univec database (ftp://ftp.ncbi.nlm.nih.gov/pub/UniVec/, accessed August 24, 2017). On average, 0.014% of each transcriptome was filtered out. Transcriptomes were then annotated for gene function using trinotate v3.0.2 (Bryant et al., 2017, accessed August 24, 2017). This pipeline uses the uniprot Swiss‐Protein database (The UniProt Consortium, 2019) and pfam database (Sonnhammer, Eddy, & Durbin, 1997, accessed August 24, 2017) to match sequences to reference proteins based on amino acid similarity.

### 2.4 Prediction of orthologous genes and phylogenetic analysis

In order to generate a reliable dataset for phylogenetic analysis, we identified orthologous genes that were shared across our study taxa. We used two approaches to identify orthologous genes among transcriptomes, both of which assign candidate sequences to predefined orthologous groups based on amino acid similarity. First, we used orthograph v0.5.4. (Petersen et al., 2017), which uses a best reciprocal hit criterion to map transcripts to the globally best matching orthogroup, while tracking best matches for all query sequences to avoid redundancy. For orthograph, we used a custom‐made ortholog set specifically designed for Polyneoptera taxa comprising 3,247 protein‐coding genes for four reference species: *Ephemera danica*, *Ladona fulva*, *Zootermopsis nevadensis*, and *Rhodnius prolixus* (for more detail see: Evangelista et al., 2019). Ortholog prediction of transcripts for each of the 26 taxa in our study was done in orthograph using the following settings: max‐blast‐searches = 50; blast‐max‐hits = 50; extend‐orf = 1; substitute‐u‐with = X. Second, we used the program hamstr v13.2.3 (Ebersberger, Strauss, & von Haeseler, 2009), which also uses a best reciprocal hit criterion, but does not track matches across query sequences. We used the Insecta v3.2 reference dataset of known orthologs (1,668 genes), with *Bombyx mori* as the focal outgroup, and applied default settings in hamstr. The resulting datasets of orthologs from each method (dataset 1, "orthograph," and dataset 2, "hamstr") were examined separately in downstream phylogenetic analyses.

Nucleotide sequences were aligned for each orthologous gene in each dataset using the mafft algorithm (Katoh & Standley, 2013) in the Consensus Multiple Align tool of geneious v9 (Biomatters, Ltd.). These gene alignments were filtered for occurrence among taxa (>10 species) and then screened for outliers using the kdetrees package (Weyenberg, Huggins, Schardl, Howe, & Yoshida, 2014) in r v3.5.3 (R Core Team, 2017). To run kdetrees, neighbor‐joining trees were produced for each alignment using the *bio‐nj* function in phangorn (Schliep, 2010), assuming the K80 substitution model. Outlier tests were conducted based on branch length distributions in the gene sets.

We used both a species tree approach and a concatenated analysis to estimate phylogenetic relationships among the study taxa. In the species tree approach, independent estimates from each gene are used to jointly infer the evolutionary divergence in a multi‐species coalescent framework (Edwards et al., 2016). This approach accounts for the possibility of incomplete lineage sorting leading to statistical inconsistency in the estimated relationships among taxa, but not for hybridization (which we assume does not occur in our dataset). While some authors have criticized this approach for deep phylogenetic reconstruction (Gatesy & Springer, 2014), investigations of the performance of these methods have shown they are robust to recombination, gene tree heterogeneity, and some uncertainty in nucleotide alignments (Liu et al., 2017; Wu, Song, Liu, & Edwards, 2013). However, substantial missing data can introduce bias in phylogenetic reconstruction (Wiens, 2006), so we generated a third dataset by combining unique genes from datasets 1 and 2 (to maximize the total number of retained genes) and pruned the data to a subset of genes where all taxa were represented. Separate species tree analyses were conducted for all three datasets in beast v2.6.0 (Bouckaert et al., 2019) to estimate individual gene trees. We made several assumptions to reduce the number of free parameters in beast. First, we selected the HKY substitution model for each gene partition, allowing for substitution rate variation across nucleotides and nucleotide classes. Additionally, a strict clock model was selected for each gene and the Yule tree prior was chosen. The MCMC chain length was set to 5 × 10^9^ steps to ensure enough search time, and the data were stored every 1 × 10^5^ steps. tracer (Rambaut & Drummond, 2009) was used to assess stationarity of the MCMC chain and to ensure that the effective sample size (ESS) of the parameters was high (>200) after burnin samples were discarded. A maximum clade credibility tree was estimated for each gene in the beast treeannotator module. These gene trees were then used to estimate a species tree in the program astral‐ii v4.10.12 (Mirarab & Warnow, 2015). This method applies the multi‐species coalescent framework to quartets of taxa in order to infer the species phylogeny, given the input gene tree bipartitions found in the gene set. Support for each quartet is measured as a local posterior probability value and branch lengths are measured at internal nodes in terms of coalescent units (small values indicate a large amount of gene tree discordance). Tree images were generated for each dataset using the ggtree package (Yu, Smith, Zhu, Guan, & Lam, 2017) in R. To compare differences among trees generated by each dataset, we used the Kendall–Colijn metric (Kendall & Colijn, 2016) of pairwise differences among tree topologies. Trees can be compared by counting the number of edges or the branch lengths between two tips and their most recent common ancestor. The R package treespace (Jombart, Kendall, Almagro‐Garcia, & Colijn, 2017) was used to compare trees based on topology, as well as branch lengths, using the multidist function.

Phylogenetic analysis of the concatenated amino acid data from dataset 1 (orthograph) was conducted as an alternative approach to tree inference. The largest dataset was used, without filtering for taxonomic representation. Orthologous genes/groups were aligned applying the L‐INS‐i algorithm of mafft v.7.221 (Katoh & Standley, 2013) at the translational (amino acid) level. Each multiple sequence alignment (MSA) was assessed and outliers were removed using the procedure outlined by Misof et al. (2014), but using the ‐addfragments algorithm implemented in mafft. Gap‐only positions were removed, and ambiguously aligned or randomized MSA sections were identified for each amino acid MSA with aliscore v.1.2 and removed with alicut v.2.3 (Kück et al., 2010; Misof & Misof, 2009). aliscore was run with the default sliding window size, the maximal number of pairwise sequence comparisons (option ‐r) and a special scoring for gap‐rich amino acid data (option ‐e). The final MSAs were concatenated into a supermatrix using fasconcat‐g v.1.02 (Kück & Longo, 2014). From this supermatrix, we further removed gene partitions with an information content (IC) of zero as identified by mare v.0.1.2‐rc (Misof et al., 2013). Prior to tree reconstruction, the best scoring amino acid substitution matrix for each gene partition was selected with modelfinder (Kalyaanamoorthy, Minh, Wong, von Haeseler, & Jermiin, 2017) as implemented in iq‐tree v.1.6.12 (Nguyen, Schmidt, Von Haeseler, & Minh, 2015). We restricted the search of the best fitting model to eight amino acid substitution matrices appropriate for nuclear markers: dcmut (Kosiol & Goldman, 2005), jtt (Jones, Taylor, & Thornton, 1992), lg (Le & Gascuel, 2008), poisson, pmb (Veerassamy, Smith, & Tillier, 2003), vt (Müller & Vingron, 2000), and wag (Whelan & Goldman, 2001). We additionally included the protein mixture model lg4x (Le, Dang, & Gascuel, 2012), which assumes no distribution site rates. Furthermore, we tested the default rate heterogeneity types (E, I, G, I + G, and FreeRates: R), with or without empirical rates (‐F, ‐FU), as well as the number of rate categories ranging from 4 to 15 (Gu, Fu, & Li, 1995; Soubrier et al., 2012; Yang, 1993). The best model for each gene partition was selected according to the best second‐order or corrected Akaike information criterion (AICc) score (Hurvich & Tsai, 1989).

Phylogenetic relationships were then inferred under the maximum likelihood (ML) optimality criterion as implemented in iq‐tree, using the best model for each gene partition and allowing partitions to have different evolutionary rates (option ‐ssp). We performed 50 independent tree searches (25 searches with a random start tree and 25 with a parsimony‐inferred start tree). To assess the number of unique topologies present within the 50 inferred trees, we used the software unique tree v.1.9 (T. Wong, unpublished). Node support was estimated via nonparametric bootstrapping of 100 bootstrap replicates in iq‐tree and mapped onto the ML tree with the best log‐likelihood. The resulting tree topologies were visualized using the ggtree package in R. As this analysis resulted in alternative ML tree topologies (see Section 3), we applied Four‐cluster Likelihood Mapping (FcLM; Strimmer & Von Haeseler, 1997) in combination with permutation analyses (Misof et al., 2014) to determine support for two specific phylogenetic relationships in the Grylloblattodea clade: (a) *G. yezoensis* and (b) *G. pravdini* (see Tables S3 and S4 for respective species included in the groups). Alternative topologies might be caused by confounding signal due to among‐lineage heterogeneity (heterogeneous composition across amino acid sequences/nonstationary substitution processes) that violate globally stationary (permutation I), a nonrandom distribution of missing data (permutation II), or a mixture of both (permutation III). A detailed explanation of the procedure can be found in Simon, Blanke, and Meusemann (2018). FcLM analyses were performed for the concatenated supermatrix used for the ML analyses using IQ‐TREE. We used the lg substitution model for each partition in this analysis.

### 2.5 Gene expression analysis

In order to identify gene expression responses to temperature exposure, differential expression (DE) of control versus acute thermal stress treatments was examined for each species/population separately using its own de novo transcriptome as reference. We deemed this necessary, as extensive genetic differences among taxa in the focal samples limit our ability to use a single set of reference sequences. As a consequence, we do not present direct statistical comparisons of DE results among taxa. Instead, we compare the annotation of top differentially expressed genes and the statistically significant tests for enrichment of gene ontology terms among taxa. We focus particularly on species where at least two biological replicates were available for all treatments (*K. biedouwense*, *G. yezoensis*, *G. pravdini*, *Grylloblatta bifratrilecta*, *Grylloblatta* sp. "Lillburn Cave," and *Grylloblatta* sp. "North Cascades" at Whitechuck Mountain), but reference the other species with unbalanced sampling designs or a lack of replicates for generalized patterns.

The following steps were implemented using helper scripts provided in the trinity analysis pipeline. The software *bowtie 2* v2.3.4 (Langmead & Salzberg, 2012) was used to map filtered reads to the reference transcriptome for each species/population. Transcript abundance was estimated using rsem v1.3.1 (Li & Dewey, 2011), which calculates expression levels based on the number of aligned reads per transcript and normalizes the expression data in terms of transcripts per million transcripts (TPM). We estimated the TPM at the gene level using a scaled TPM, which pools results from similar transcripts and adjusts for differences in isoform lengths in calculating expression levels. To compare the relative expression among samples, these values were further adjusted using the weighted trimmed mean of the log expression ratios (TMM) as a method of cross‐sample normalization (Robinson & Oshlack, 2010). We chose to calculate DE in the R package edger (Robinson, McCarthy, & Smyth, 2009). As some species lacked biological replicates, this package allows for expression values to be compared by assuming a dispersion value (here, we chose 0.16, based on default recommendations) for among‐sample variance in background expression. We then extracted highly differentially expressed transcripts based on a false discovery rate of *α* = 0.01 and 4‐fold difference in expression among treatments for enrichment analyses. Gene ontology categories were assessed for enrichment in the thermal stress treatments by examining the number of highly expressed genes in each category relative to their proportional abundance in the annotated transcriptomes, using the goseq package in R (Young, Wakefield, Smyth, & Oshlack, 2010).

### 2.6 Positive selection in protein‐coding genes

In order to detect protein‐coding genes under positive selection, we employed two bioinformatics pipelines: posigene (Sahm, Bens, Platzer, & Szafranski, 2017) and fustr (Cole & Brewer, 2018). Both methods identify and align orthologs, and then conduct a branch‐site test of positive selection on orthologous genes, but the former focuses on evidence of selection along the branches of a species phylogeny for each orthogroup alignment, while the latter method focuses on evidence of selection along the branches of a gene family phylogeny. For both methods, the entire transcriptome of each species was used as input, and subsequent pipelines were used to determine orthology and sequence alignment. Each method relies on either nucleotide sequence (posigene) or protein sequence (fustr) similarity to determine orthology. As a consequence, true orthologs that are highly divergent in their sequence similarity or only partially represented in the transcriptomic data will not be represented in this analysis.

We note that branch‐site tests can have an inflated false‐positive rate when multinucleotide mutations occur in codons (Venkat, Hahn, & Thornton, 2018), as the test statistic is not calibrated for such mutations. While this could be a source of bias in our downstream analysis, we note that there is disagreement about whether sites with multinucleotide are evolving neutrally (false positives) or are in fact evidence of strong positive selection (Belinky, Sela, Rogozin, & Koonin, 2019). Furthermore, Jones, Youssef, Susko, and Bielawski (2018) suggest that it is not yet possible to attribute branch‐specific false positives (type I error rate) to multinucleotide mutation models in real data due to confounding effects, and implementations of multinucleotide models can result in a substantial loss in power (Dunn, Kenney, Gu, & Bielawski, 2019).

posigene requires a phylogenetic tree for the branch‐site test of positive selection in codeml (Yang, 2007; Yang & Nielsen, 2002), and both an anchor species and a target species are designated from the data set. The anchor species serves as the basis for matching isoforms of this species to the best matching isoform of every other species in the data set. The target species denotes the evolutionary branch of the input tree to be tested for positive selection, and multiple species within a clade may be designated as the target species. We used the species tree from dataset 1 as the input phylogeny, and *Grylloblatta bifratrilecta* was chosen as the anchor species as it had the most complete data set in Grylloblattodea. The Grylloblattodea clade and the subclade *Grylloblatta* were chosen as target species in alternative runs of posigene. In the first step of posigene, ortholog groups were assigned for all input species using a bi‐directional blast and then aligned with clustalw (Larkin et al., 2007). Using the anchor species as the reference, multiple sequence alignment in clustalw was used to select a single matching isoform across species. For isoforms found in a minimum of three species, prank (http://wasabiapp.org/software/prank/) was used to align sequences at the codon level. An additional filtering step using gblocks was used to remove unreliable alignment regions and to reduce the likelihood of false positives being identified in branch‐site tests (Castresana, 2000). codeml was then used to conduct branch‐site specific tests for positive selection along the designated branches.

In the alternative approach to detecting positive selection, fustr was chosen as it focuses on tests of selection in gene families, irrespective of their representation among taxa (some taxa could entirely lack members of the gene family in their reconstructed transcriptome). Isoforms were identified among input sequences, and transdecoder (https://github.com/TransDecoder/TransDecoder) was used to extract the best open reading frame of each transcript. These coding sequences were assessed for homology at the peptide level using diamond (Buchfink, Xie, & Huson, 2015), grouped into gene families using silix (Miele, Penel, & Duret, 2011), and then aligned within each family using mafft (Katoh & Standley, 2013). Families with at least 15 sequences were tested for positive selection at the site‐specific amino acid level using the branch‐site test implemented in fubar (Murrell et al., 2013).

Genes identified as under positive selection by either method were cross‐referenced to look for convergence in the two approaches, and also compared to the list of top differentially expressed genes to determine whether positive selection and gene regulatory evolution operated on the same genes.

## 3 RESULTS

### 3.1 Transcriptome assemblies and ortholog datasets

Transcriptome assemblies varied in size, contig number, and completeness (Table S5). For the taxa that were used in downstream differential expression analysis, the average value for the percent of complete BUSCOs score was 74.35% (min: 59.8%, max: 89.6%). From the orthograph analysis (dataset 1), approximately 3,200 orthologs were identified, whereas approximately 1,575 orthologs were identified using hamstr (dataset 2). For the species tree analyses, both datasets were filtered with the requirement that each gene represent >10 of the 26 taxa, resulting in 877 and 820 genes, respectively. A small number of genes (13) from dataset 2 were identified as outliers in kdetrees and removed. The final datasets comprised 877 genes (dataset 1: ~675 kb, 24.7% missing nucleotide data) and 809 genes (dataset 2: ~810 kb, 51.5% missing nucleotide data). We used reciprocal blast and found that roughly ~35% of the genes were shared among datasets, so the datasets were analyzed separately to provide alternative estimates of the phylogeny. A third dataset was generated to reduce missing data, by combining unique genes from datasets 1 and 2 and filtering so that every taxon had a representative sequence per ortholog. This dataset comprised 422 genes (dataset 3: ~292 kb, 18.8% missing nucleotide data). The amino acid dataset for the concatenated ML analyses comprised 3,022 genes, 1,075,332 amino acids, and 24% missing data.

### 3.2 Phylogenetic analysis

**FIGURE 1:**
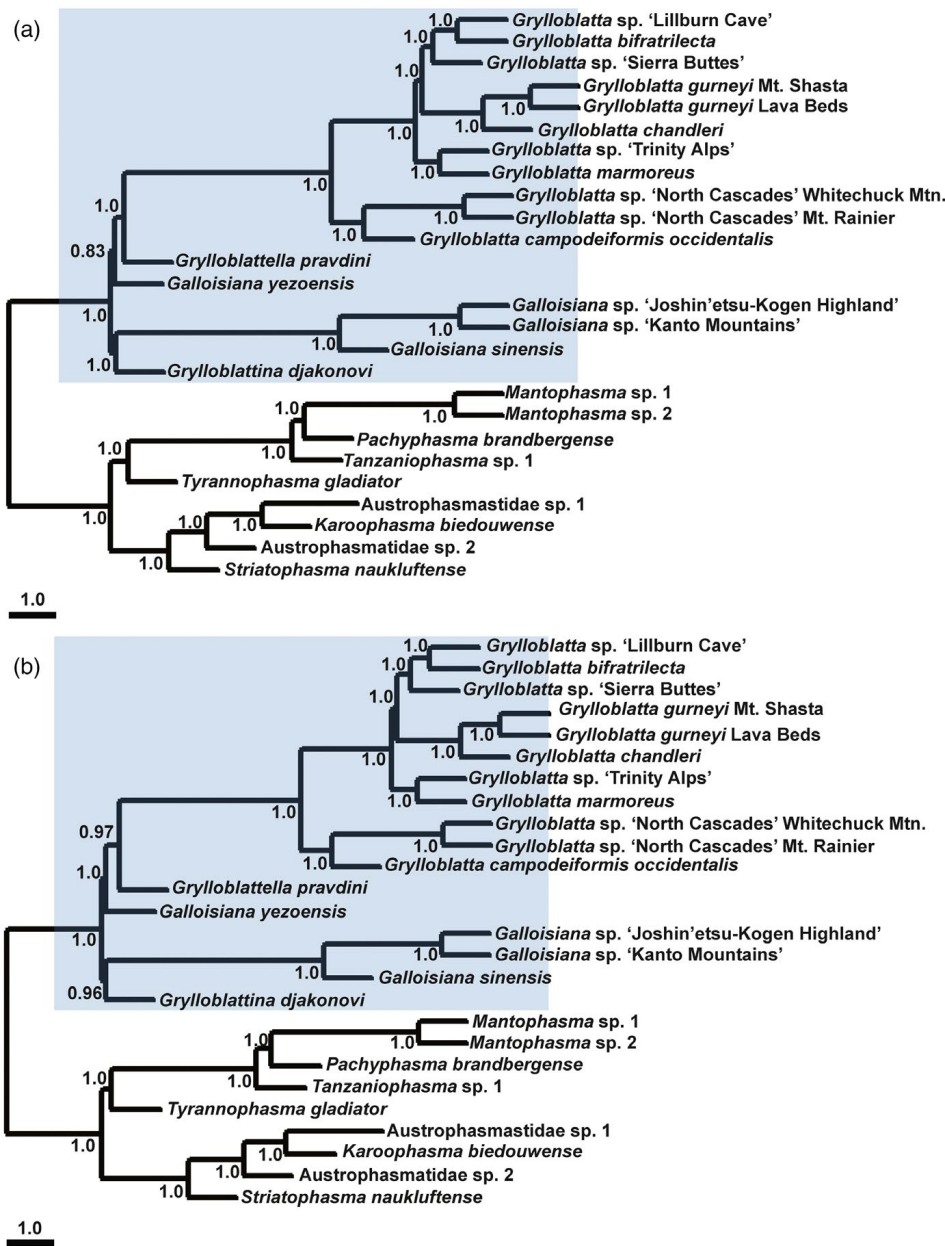
Multispecies coalescent trees inferred from astral‐ii showing the phylogenetic relationships of Mantophasmatodea and Grylloblattodea. Results are shown for (a) dataset 1 from orthograph, with 877 genes, and (b) dataset 3, merging 422 unique genes from dataset 1 and 2 with complete taxon sampling. Numbers at nodes indicate local posterior probability, and branch lengths are represented in coalescent units

Phylogenetic estimates based on species tree analysis (Figure 1) provide strong support for the monophyly of Mantophasmatodea and Grylloblattodea, and consistent relationships among taxa within the Grylloblattodea. The topology inferred from dataset 1 (Figure 1a) matched the topology estimated in dataset 3 (Figure 1b), although branch support increased as missing data was removed. Relationships varied in dataset 2 (see the phylogenetic tree from dataset 2 in Figure S1), where the topology differed in one subclade of the Mantophasmatodea (comprising Austrophasmatidae sp. 1 and sp. 2, *K. biedouwense*, and *S. naukluftense*). These differences are evident in the Kendall Colijn metric, which shows high pairwise topological distances of either dataset 1 or 3 to dataset 2 (10.0995; note, there are no differences between dataset 1 and 3). When branch lengths are considered in the Kendall Colijn metric, small differences are seen in dataset 1 versus 3 (4.78), but differences remain large for either dataset when compared to dataset 2 (42.186 and 38.295, respectively). We examined the amount of missing data per base pair for rearranged taxa and found that amounts were much higher in dataset 2 (80.5%, 82.8%, and 58.8% for Austrophasmatidae sp. 1, Austrophasmatidae sp. 2 and *S. naukluftense*, respectively) than in dataset 1 (38.1%, 29.2%, and 32.0%, respectively) or dataset 3 (21.6%, 14.1%, and 17.7%, respectively).

**FIGURE 2:**
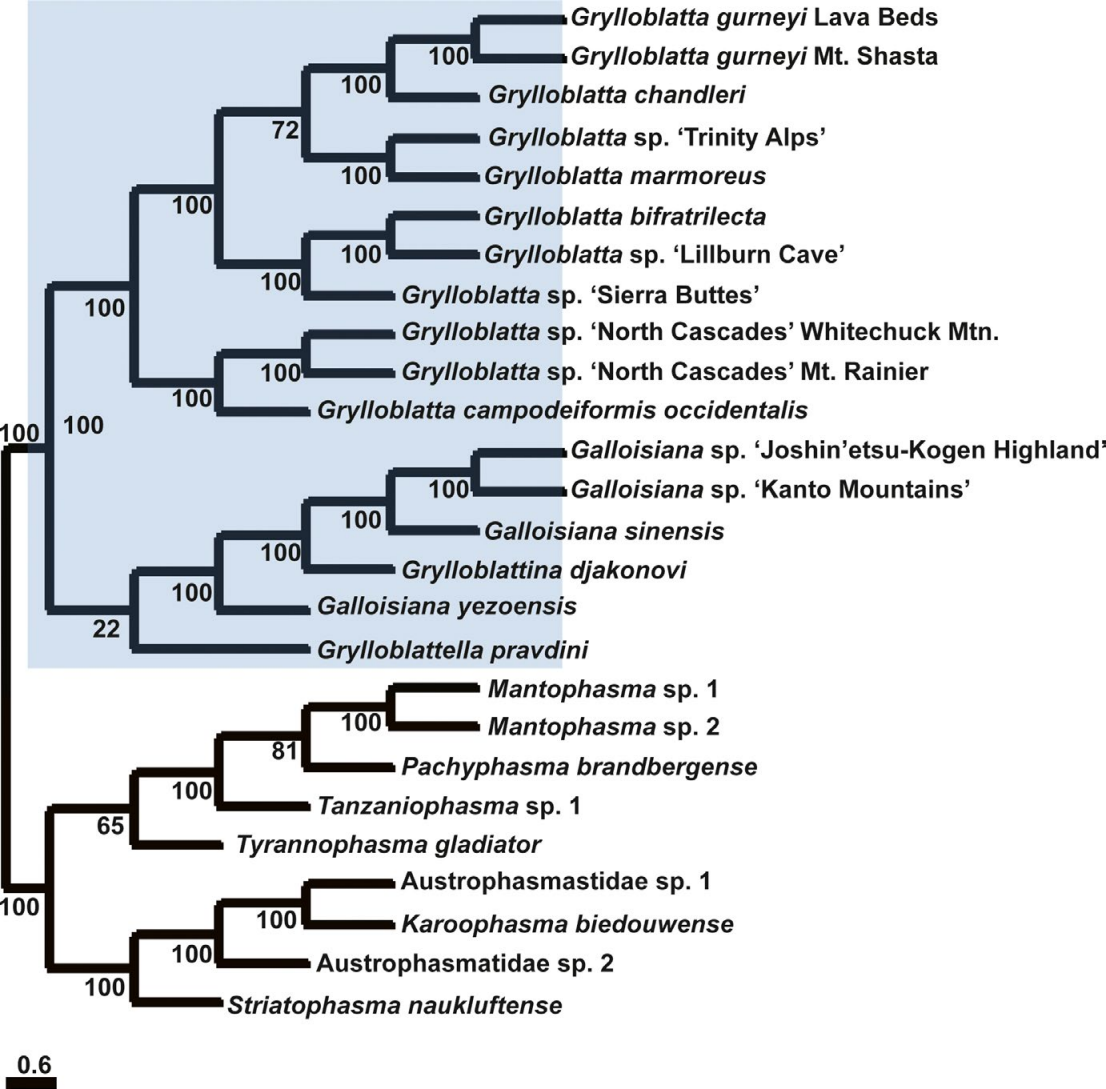
The maximum likelihood tree inferred from the concatenated amino acid dataset of 3,022 genes. This ML tree received the best log‐likelihood score 39 times across 50 independent tree searches. Node support values were estimated based on 100 bootstrap replicates. Branch lengths are unscaled

A maximum likelihood (ML) analysis of the concatenated amino acid data from Dataset 1 was run for 50 independent tree searches. Three alternative tree topologies emerged from these searches, with the best topology (Figure 2) supported 39 times. The other topologies were recovered eight times and three times, respectively (Figure S2). Notably, all three topologies are broadly concordant with the species trees (Figure 1), including relationships within Mantophasmatodea. One difference in the ML trees and the species trees involves a more nested relationship of *Grylloblatta marmoreus* and *G*. sp. "Trinity Alps" within *Grylloblatta* taxa, but bootstrap support for this relationship in the ML tree is modest. A second difference involves the placement of sister taxa to the North American *Grylloblatta*, where the three ML trees differ from the species tree and from one another. The most frequently inferred ML tree (Figure 2) suggests that all Asian Grylloblattodea form a reciprocally monophyletic group sister to North American *Grylloblatta*. However, bootstrap support for a sister‐group relationship of *Grylloblatella pravdini* to other Asian Grylloblattodea is very weak.

Therefore, in addition to the nonparametric bootstrap support, we assessed support for specific phylogenetic relationships with the aid of the Four‐cluster Likelihood Mapping (FcLM) method. All quartets (100%, 44 unique quartets) unambiguously supported a sister‐group relationship of *G. yezoensis* to the other East Asian Grylloblattodea species. Further permutation tests showed that heterogeneous amino acid site composition, nonstationary substitution processes, and missing data do not affect this inferred relationship (Table S6). This is in contrast to the species tree which places *G. yezoensis* at the base of North American *Grylloblatta* + *G. pravdini*. However, it has to be noted that the number of drawn quartets was low (44 unique quartets). Therefore, the FcLM results might be unreliable without further taxon sampling. We also evaluated the support for a sister‐group relationship of *G. pravdini* to the East Asian Grylloblattodea clade as inferred in the ML concatenated analyses (Figure 2). The FcLM analyses revealed that this retrieved relationship might be biased. Around 4/5 of all quartets (86.9%, 495 unique quartets) supported the sister‐group relationship of *G. pravdini* to *Grylloblatta* as inferred in the species tree (Figure 1). Further permutation analyses show that this support—*G. pravdini* sister to the clade *Grylloblatta*—is not biased by confounding signal (Table S7).

### 3.3 Differential expression analysis

**FIGURE 3:**
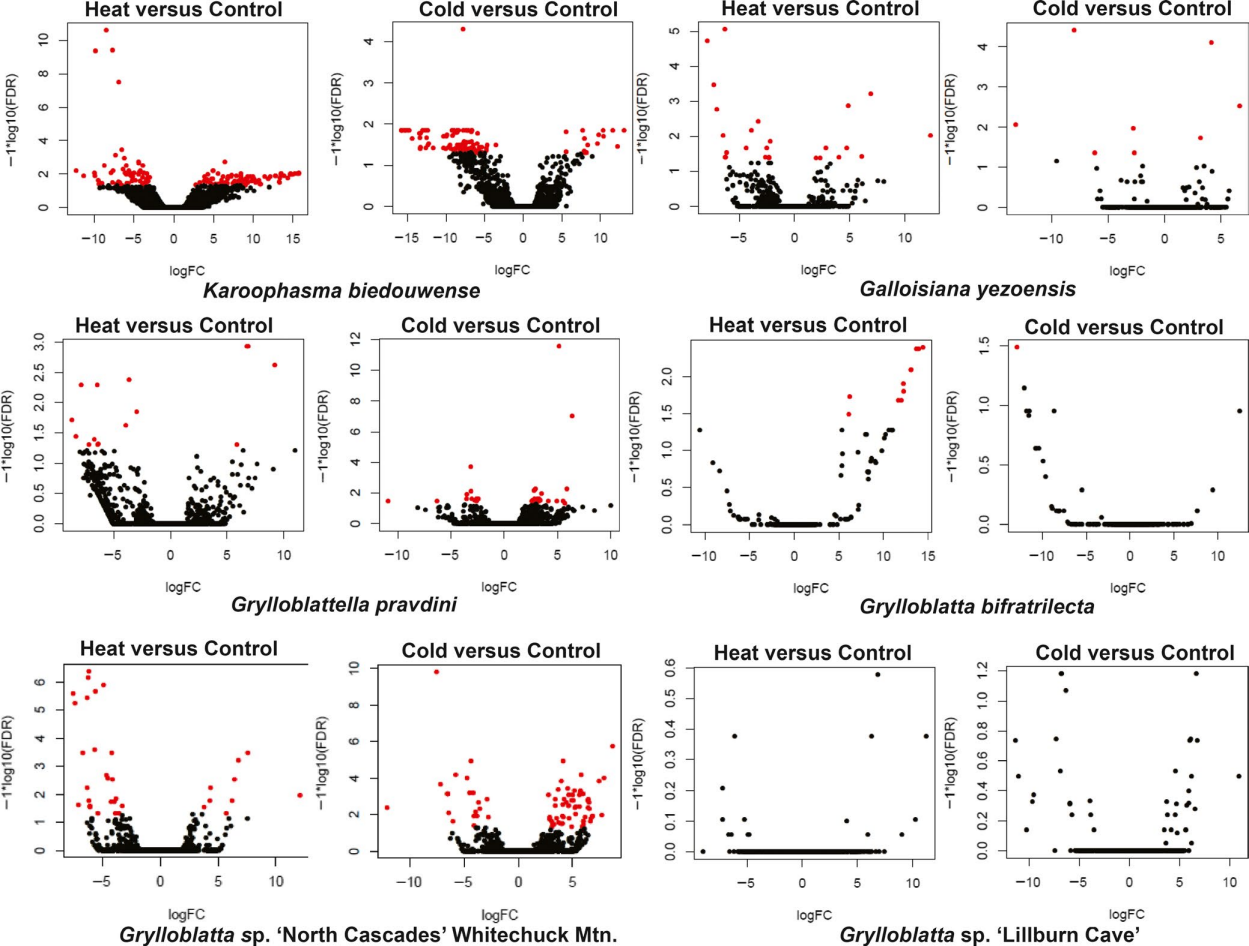
Volcano plots of the differential expression analysis in acute temperature stress experiments. Plots show the statistical significance (*y*‐axis, log FDR value) and relative expression of genes (*x*‐axis, log fold change) under acute heat or acute cold stress relative to control samples. Significantly differentially expressed genes are indicated in red

Strong patterns of differential expression are observed in the acute heat stress response (when compared to control samples) in the mantophasmatodean *K. biedouwense* (Figure 3). The most highly differentially expressed genes include ten canonical genes in the heat shock protein (*hsp*) family related to heat stress (*hsp70*, *hsp68*, *hsp20.5*, *alpha‐crystallin*, and *protein lethal(2) essential for life* in the *hsp20* family; Table S8). The pattern of differential expression was less pronounced in the acute cold stress treatment of *K. biedouwense*, but included several genes associated with managing oxidative stress (TAR1 and ART3) and a lysozyme associated with immune function (Table S9). In contrast, differential expression is weak, with few significantly expressed genes in the grylloblattodean species. This is most pronounced in the North American *Grylloblatta* lineage. Examining the top differentially expressed genes in the acute heat stress treatments, only a single hit in *G. yezoensis* is related to heat stress (*hsp70 B2*; Table S10). Examining the top differentially expressed genes in the acute cold stress treatments reveals no shared pattern across *Grylloblatta* species, except for select cases where proteolytic function is upregulated and the cellular respiration pathway in the mitochondrion is downregulated (Table S11).

Under acute heat stress, tests for enrichment of gene ontology terms resulted in a number of categories directly related to a temperature stress response in *K. biedouwense* (GO:0009408: response to heat, GO:0009266: response to temperature stimulus, GO:0035080: heat shock‐mediated polytene chromosome puffing, GO:0034605: cellular response to heat; Table S12), in addition to a general response to stress (GO:0043558: regulation of translational initiation in response to stress, GO:0043555: regulation of translation in response to stress, GO:0051082: unfolded protein binding, GO:0042026: protein refolding, GO:0006457: protein folding, GO:0061077: chaperone‐mediated protein folding, GO:0009628: response to abiotic stimulus, GO:0033554: BP cellular response to stress, GO:0006950: response to stress, GO:0051716: cellular response to stimulus). No categories related to temperature stress were enriched under the acute cold stress in *K. biedouwense*. Across multiple genera and species of Grylloblattodea, no categories related to temperature stress were consistently enriched following an acute heat stress. These results were consistent whether species with biological replicates, or those lacking biological replicates (Table S13), were examined. Following acute cold stress, however, some *Grylloblatta* species showed responses involving upregulation of oxidative stress and inflammation pathways (GO:0000302: BP response to reactive oxygen species, GO:0006979: BP response to oxidative stress, GO:0050727: BP regulation of inflammatory response). Interestingly, protein degradation (GO:0006508: proteolysis) was both up‐ and downregulated within and across species, suggesting fine tuning of this pathway.

### 3.4 Constitutive expression of heat shock proteins

**FIGURE 4:**
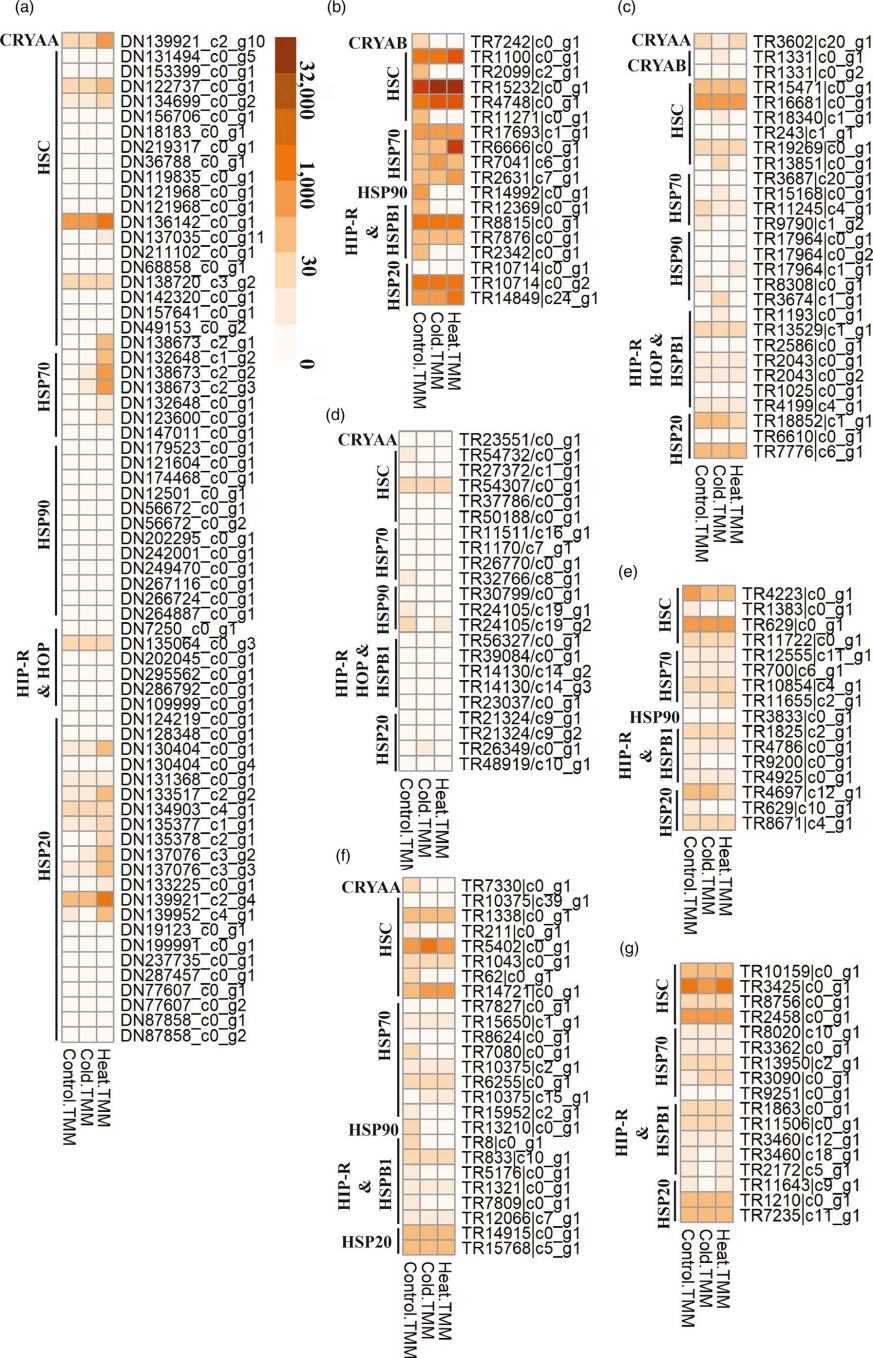
Heat map of gene expression for members of the heat shock protein (*hsp*) gene family. Mean results are shown for control, acute cold, and acute heat treatments for: (a) *Karoophasma biedouwense*, (b) *Galloisiana yezoensis*, (c) *Grylloblattella pravdini*, (d) *Grylloblatta bifratrilecta*, (e) *Grylloblatta marmoreus*, (f) *Grylloblatta* sp. "North Cascades" at Whitechuck Mountain, and (g) *Grylloblatta* sp. "Lillburn Cave." Expression values represent normalized within‐species trimmed mean of M (TMM) values, and are shown on a log_2_‐scale color gradient from off‐white to red. Refer to Table S14 for observed TMM values

To determine whether constitutive levels of *hsp* gene expression were simply higher in Grylloblattodea, we compared mean levels of expression for members of the heat shock protein family across treatments (a heat map is shown in Figure 4; the TMM expression values are provided as Table S14). While *hsp* levels in the control samples were low for genes that were induced during the heat shock response in *K. biedouwense* (generally <100 TMM; *μ*_TMM_ = 3.3, *σ*_TMM_ = 251.4), levels of constitutive expression for some *hsp* genes were high for *G. yezoensis* (»200 TMM, *μ*_TMM_ = 21.8, *σ*_TMM_ = 488.1). These represented *hsp70* genes, as well as heat shock cognate (*hsc*) and *hsp20* genes that are actively expressed during development. In the remaining Grylloblattodea taxa, *hsp* expression values were generally low (<100 TMM) in *G. pravdini* (*μ*_TMM_ = 14.3, *σ*_TMM_ = 851.2) and members of *Grylloblatta* (*μ*_TMM_ = 15.2, *σ*_TMM_ = 873.1), although *hsc* and *hsp*20 genes were exceptions (>100 TMM).

### 3.5 Evidence for positive selection on proteins

We tested for evidence of selection on protein‐coding genes using two methods. The branch‐site test implemented in posigene focused on orthologous genes found in at least three representative species. A total of 53 genes were identified as candidates for positive selection at the node of Grylloblattodea, completely overlapping the set of significant genes identified at the node of North American *Grylloblatta* (Table 2). However, only a subset of these genes (10 of 53, shown in bold type Table 2) show evidence of selection in both Asian and North American lineages of Grylloblattodea, with the majority showing selection only within North American *Grylloblatta*. Based on the number of sites under selection, three genes (*protein lingerer*, *40S ribosomal protein S2*, and *apolipophorins*) were notable outliers with excessive numbers of substitutions found only in Grylloblattodea transcriptomes. Two homologs of *Tret1‐2*, the facilitated trehalose transporter, are also noteworthy as modulators of cellular trehalose levels. Other candidate genes have functions related to cell homeostasis (*nucleobindin‐2*), microtubule assembly (*tubulin alpha‐1*), cell growth (*cyclin‐dependent kinase 11B‐like isoform*, *MAU2 chromatid cohesion factor homolog*, *cleft lip and palate transmembrane protein 1*), metabolism (*sulfotransferase*, *Peptide Transporter Family 1*, *vacuolar ATPase assembly integral membrane protein vma21*), mitochondrial function (*Mitochondrial‐processing peptidase subunit alpha*, *39S ribosomal protein L40*, *FAD‐dependent oxidoreductase domain‐containing protein 1*), muscle activity (*twitchin*), protein synthesis (*60S ribosomal protein L10* and *L7*), and proteasomal degradation of proteins (proteasome subunit alpha type‐4, *hamartin*, *Derlin‐1*, *hepatocyte growth factor‐regulated tyrosine kinase substrate*).

**TABLE 2:** Orthologous genes found to be under selection using the codeml branch‐site test implemented in posigene. Ratios of dn/ds listed as not applicable (“NA”) could not be computed due to a lack of synonymous substitutions. All genes shown were identified as significant within the North American *Grylloblatta* branch, while genes in bold type include taxa across Grylloblattodea

Gene Name	Species^a^	*p*‐value^b^	dn/ds	Annotation	Sites under selection
**GBIF_TR20652_c0_g1_i1**	B, M, R, JK	0	28.84	Protein Lingerer	1,025
**GBIF_TR44978_c0_g1_i1**	B, M, YZ	2.31E‐10	NA	40S ribosomal protein S2	264
GBIF_TR21200_c1_g4_i1	B, C, S, W	2.17E‐02	1.65	apolipophorins	83
GBIF_TR12574_c0_g1_i1	B, L, W	8.56E‐03	2.54	suppressor of cytokine signaling 5	42
GBIF_TR31969_c0_g1_i1	B, G, L, O	1.67E‐05	111.87	dystroglycan	36
GBIF_TR141_c14_g1_i1	B, G, R, S	1.29E‐04	3.01	hamartin	32
GBIF_TR28586_c7_g1_i1	B, M, L, W	2.11E‐04	12.14	negative elongation factor B	29
GBIF_TR39005_c0_g1_i1	B, L, O, R	1.58E‐04	8.09	cyclin‐dependent kinase 11B‐like isoform	28
GBIF_TR36436_c0_g1_i1	B, C, S, W	4.05E‐02	2.43	Mitochondrial‐processing peptidase subunit alpha	26
GBIF_TR48084_c6_g1_i1	B, G, L	3.01E‐02	53.84	Sulfotransferase	24
GBIF_TR25767_c6_g1_i1	B, T, W	2.56E‐05	NA	tubulin alpha‐1 chain	23
GBIF_TR15620_c6_g1_i1	B, M, S, W	3.72E‐03	5.76	uncharacterized protein	20
GBIF_TR1907_c5_g2_i4	B, L, W	4.34E‐04	NA	facilitated trehalose transporter Tret1‐2 homolog	20
GBIF_TR21661_c1_g1_i1	B, M, W	1.95E‐03	9.49	WD40 repeat‐containing protein SMU1	20
GBIF_TR12904_c1_g2_i1	B, L, R	8.32E‐04	3.39	facilitated trehalose transporter Tret1‐2 homolog	19
GBIF_TR26495_c0_g1_i1	B, C, L	1.86E‐02	3.61	Unknown protein	19
GBIF_TR38730_c14_g1_i1	B, S, W	1.45E‐02	20.61	probable ATP‐dependent RNA helicase DDX28	19
GBIF_TR21121_c11_g1_i1	B, M, O	1.36E‐03	NA	MAU2 chromatid cohesion factor homolog	17
GBIF_TR8603_c0_g1_i1	B, G, R	3.41E‐02	2.09	39S ribosomal protein L40, mitochondrial	16
GBIF_TR20756_c12_g1_i3	B, O, T	8.80E‐04	NA	Nucleobindin‐2 isoform	15
GBIF_TR48876_c16_g1_i1	B, C, G, R	1.46E‐04	29.00	Pathogenesis‐related protein 5‐like	15
GBIF_TR52043_c4_g1_i1	B, L, W	1.06E‐02	3.78	FAD‐dependent oxidoreductase domain‐containing protein 1	15
GBIF_TR3939_c1_g1_i1	B, L, S, T	2.09E‐03	3.68	Unknown protein	14
GBIF_TR24210_c0_g1_i2	B, C, G, M, R, T	4.98E‐03	NA	60S ribosomal protein L10	12
GBIF_TR26361_c0_g1_i1	B, L, W	1.67E‐03	NA	acidic fibroblast growth factor intracellular‐binding protein	12
GBIF_TR51146_c13_g1_i1	B, G, L	3.26E‐03	NA	hepatocyte growth factor‐regulated tyrosine kinase substrate	12
GBIF_TR23014_c9_g1_i1	B, L, W	1.30E‐02	0.79	THO complex subunit 6	11
**GBIF_TR12351_c5_g2_i1**	B, G, M, R, YZ	1.64E‐02	0.28	Peptide Transporter Family 1	*10*
GBIF_TR21238_c0_g1_i1	B, M, W	3.27E‐03	NA	protein big brother‐like	10
GBIF_TR13087_c0_g1_i1	B, M, O	7.91E‐03	NA	60S ribosomal protein L7	9
**GBIF_TR35758_c4_g1_i1**	B, R, W, PR	4.39E‐04	40.75	transcription initiation factor TFIID subunit 12	9
**GBIF_TR48750_c6_g1_i1**	B, C, D, S, T, YZ	6.83E‐05	25.54	Chitinase‐3‐like protein 1/chitotriosidase‐1‐like	9
GBIF_TR57016_c0_g2_i1	B, L, S	2.37E‐02	NA	vacuolar ATPase assembly integral membrane protein vma21	9
GBIF_TR7004_c0_g2_i1	B, L, S	3.09E‐02	2.54	venom carboxylesterase‐6‐like	9
GBIF_TR1960_c0_g1_i1	B, M, O	3.71E‐02	0.82	WASH complex subunit 2‐like (low alignment scores)	8
GBIF_TR3384_c2_g1_i1	B, R, S	3.10E‐02	NA	Unknown protein	7
**GBIF_TR24046_c0_g1_i9**	B, L, SN	3.52E‐08	4.12	twitchin isoform	6
GBIF_TR29581_c0_g1_i1	B, L, T	4.30E‐02	NA	Derlin‐1	6
GBIF_TR9220_c2_g2_i1	B, L, S	4.96E‐02	NA	zinc finger protein 76‐like	6
**GBIF_TR42181_c0_g1_i1**	B, G, PR	9.36E‐03	1.04	Unknown protein	5
GBIF_TR6408_c12_g1_i1	B, L, W	2.17E‐03	25.66	proteasome subunit alpha type‐4	4
GBIF_TR9005_c9_g2_i1	B, L, S	4.07E‐02	NA	Unknown protein	**4**
GBIF_TR14725_c0_g1_i1	B, G, O, R, S	7.66E‐03	0.23	cleft lip and palate transmembrane protein 1 homolog	3
GBIF_TR26013_c6_g1_i1	B, C, L	1.60E‐04	8.20	Unknown protein	3
GBIF_TR8977_c1_g2_i1	B, G, L	1.05E‐03	7.07	argininosuccinate lyase isoform	3
GBIF_TR26425_c0_g1_i1	B, L, M, S	1.23E‐03	NA	transforming growth factor‐beta‐induced protein ig‐h3	2
**GBIF_TR31054_c0_g1_i1**	B, L, M, R, W, PR, YZ	5.47E‐03	227.00	Titin‐like isoform	2
GBIF_TR43225_c0_g1_i1	B, L, O	1.31E‐04	4.24	facilitated trehalose transporter tret‐1	2
GBIF_TR16612_c0_g1_i1	B, R, S	4.40E‐04	1.87	general transcription factor 3C polypeptide 1	1
GBIF_TR1865_c0_g1_i1	B, M, R	2.12E‐02	3.51	lysosomal Pro‐X carboxypeptidase	1
**GBIF_TR42441_c23_g1_i1**	B, L, M, O, R, S, W, PR, YZ	1.18E‐03	33.18	*basigin/neuroplastin*	1
GBIF_TR45202_c6_g2_i1	B, L, R	2.35E‐02	5.81	mitochondrial carrier protein Rim2	1
**GBIF_TR5293_c0_g1_i1**	B, G, R, PR, MS2	1.37E‐02	5.92	*Mitotic Checkpoint Protein BUB3*	1

^a^ Species abbreviations: B = *Grylloblatta bifratrilecta*, G = *Grylloblatta gurneyi*, L = Grylloblatta sp. "Lillburn Cave," M = *Grylloblatta marmoreus*, O = *Grylloblatta campodeiformis occidentalis*, R = *Grylloblatta* sp. "North Cascades" Mt. Rainier, S = *Grylloblatta* sp. "Sierra Buttes," T = *Grylloblatta* sp. "Trinity Alps," W = *Grylloblatta* sp. "North Cascades" Whitechuck Mtn., JK = *Galloisiana* sp. "Joshin'etsu‐Kogen Highlands," PR = *Grylloblattella pravdini*, SN = *Galloisiana sinensis*, YZ = *Galloisiana yezoensis*, and MS2 = *Mantophasma* sp.

^b^ Genes with *p* < .05 are shown. The *p*‐value shown is the raw unadjusted *p*‐value for the likelihood ratio test calculated using a *χ* ^2^ distribution with one degree of freedom. The dn/ds ratio compares the rate of nonsynonymous mutations to synonymous mutations to measure the strength of selection. Larger values indicate stronger selection, all values >1 indicate positive selection.

The branch‐site test implemented in fustr focused on gene families under selection, irrespective of representation among taxa. We pruned the results to those families where at least three codon sites were identified as under selection to reduce the false positive rate, resulting in 154 gene families (Table S15). Grouping gene families by biological function shows a large number of families related to stress or immune response (31 gene families), particularly in protein folding and the proteolytic response pathway (including two *hsp*s). Other key biological functions include oxygen reduction and cellular respiration (13 gene families), metabolism and development (31 gene families), DNA replication and protein synthesis (43 gene families), and olfaction and nervous system function (seven gene families). Interestingly, the signature of positive selection on odorant‐binding proteins appears to be restricted to the Grylloblattodea. Candidate selection genes did not directly overlap in test results from posigene and fustr, except for one gene (pathogenesis‐related protein 5‐like) involved in pathogen resistance. Furthermore, there was no overlap in the results from posigene and the differential expression analysis of thermal stress, while only one ortholog group identified in fustr (apolipoprotein D‐like protein) overlapped with the differential expression analysis (as significantly downregulated during cold stress in *G*. sp. "North Cascades" Whitechuck Mountain). However, we did observe broad overlap in gene function among tests, which included proteolytic activity, lipid metabolic processes (lipophorin activity), regulation of mitochondrial respiration, and protein synthesis.

## 4 DISCUSSION

Here, we tested the question of whether the relict insect lineage Grylloblattodea (Vrsansky et al., 2001; Wipfler et al., 2014) provides molecular evidence of evolutionary constraints in the physiological tolerance of temperature. Previous research has shown that thermal breadth and critical thermal limits do not vary in North American species (Schoville et al., 2015), consistent with constraints on thermal niche evolution. In addition, lineages in Asia have shifted into subterranean habitats with subsequent eye loss where they have persisted in warm climates (Schoville & Kim, 2011). First, we examined whether acute thermal stress activates gene expression differentially in members of Grylloblattodea. We found that Grylloblattodea lack an inducible heat shock response typical of most animal species (Kültz, 2005b), including its sister lineage Mantophasmatodea. Although these results should be interpreted with some caution, as we did not assay protein activity in ice‐crawlers and only focused on rapid responses (~30 min) to acute temperature stress (Evans, 2015), additional evidence from selection on protein‐coding genes is consistent with evolutionary constraints due to cold specialization (Logan & Buckley, 2015; Overgaard & MacMillan, 2017; Storey & Storey, 2012). Below we discuss the evidence supporting evolutionary constraints in this relict lineage, as well as insights that transcriptomic analysis provides on the evolutionary diversification of Grylloblattodea and Mantophasmatodea, and conservation of these rare lineages.

### 4.1 Heat shock responses and cold specialization

Heat shock proteins (*hsp*s) are molecular chaperones that are typically highly conserved in diverse organisms (from bacteria to eukaryotes), with strong purifying selection and gene conversion maintaining nucleotide similarity across divergent lineages (Hess et al., 2018). Similarly, the heat shock response is a canonical part of the cellular stress response (Evgen’ev, 2014; Kültz, 2005a), with upregulation of *hsp*s occurring under stressful conditions to prevent damage to cellular proteins. For example, studies in *Drosophila melanogaster* have shown that the inducible heat shock genes, particularly the *hsp70* family, are critical to heat resistance (Takahashi, Okada, & Teramura, 2011). Mutant lines that lack *hsp70* have reduced thermal tolerance and experience high mortality following heat and cold shock (Gong & Golic, 2006; Štětina, Koštál, & Korbelová, 2015). This may be linked to cell cycle arrest and induction of the apoptosis pathways as cellular damage accumulates (Logan & Buckley, 2015). Interestingly, a suite of additional *hsp*s (i.e., *hsp20* and *hsp90*) are upregulated in these *hsp*70‐null lines following acute heat stress (but not acute cold stress), suggesting some redundancy in function among *hsp*s, although these proteins do not fully compensate for the loss of *hsp70* function (Bettencourt, Hogan, Nimali, & Drohan, 2008; Štětina et al., 2015). The induction of the heat shock response is also widely observed following cold stress in insects (Storey & Storey, 2011). In contrast, our results show a complete lack of constitutive or inducible *hsp* expression in two genera within Grylloblattodea (*Grylloblattella* and its sister lineage *Grylloblatta*). Although one *hsp70* is expressed under heat stress in *G. yezoensis*, the upregulation of a single gene differs strongly from the dramatic upregulation of multiple *hsp* genes in the mantophasmatodean *K. biedouwense* (Figure 3), or heat shock response in other insects (King & MacRae, 2015), as evidenced by enrichment tests. There are caveats to these results. First, there may be limitations in the experimental design we have chosen for sampling gene expression patterns. For example, it is possible that heat shock responses might be triggered at other temperatures, or under different durations of stress, than what we experimentally tested. In the cold treatments specifically, it has been shown that rapid exposure to extremes (as was done in our study) might result in little to no gene expression (Teets, Gantz, & Kawarasaki, 2020). While we favor the interpretation that heat shock responses have been lost in Grylloblattodea (given additional data, see below), we note that further experimental validation is needed.

Alteration of the conserved stress response (Kültz, 2005) to reduce induction of *hsp*s could arise if the pathway is energetically expensive and rarely needed, or alternatively if the *hsp*s are already present in high quantities due to constitutive expression (Tomanek, 2008). As mentioned earlier, stenothermal Antarctic fish similarly show the loss of inducible heat shock responses (Logan & Buckley, 2015), possibly as a result of the disruption of cis‐regulatory binding sites (Bogan & Place, 2019). Interestingly, *hsp*s remain active in these fish through constant, constitutive expression (Place & Hofmann, 2005). Similar patterns are found in animals inhabiting xeric environments, which maintain heat shock proteins at physiologically moderate temperatures and thus do not need to rely on a rapid upregulation of *hsp*s during acute thermal stress (Evgen’ev, Garbuz, Shilova, & Zatsepina, 2007). Other species exposed to chronically stressful or fluctuating thermal environments also show constitutive expression of *hsp*s (Tomanek, 2008). For example, fly species from four different families show high constitutive expression, with the level of expression increasing in the most heat‐resistant species (Zatsepina et al., 2016). Cold‐hardy insects often go through seasonal acclimation that includes accumulation of *hsp*s that can mask an acute cold shock response (Storey & Storey, 2012). High constitutive expression of *hsp70* is seen in *G. yezoensis* (compared to levels seen in *K. biedouwense*). Other Grylloblattodea show some high levels of *hsc* and *hsp20* genes, but these genes are known to be constitutively expressed during development. Therefore, it does not appear that constitutive upregulation broadly applies to Grylloblattodea (Figure 4), although we have only examined constitutive levels under a limited set of conditions.

Grylloblattodea species occupy habitats that have constant, cool temperatures throughout the year, such as subterranean, cavernicolous, subnival, and talus environments (Goodrich, 1982; Kamp, 1970; Millar, D. Westfall, & Delany, 2014). This may suggest that constitutive expression of *hsp* genes is not involved in their cold specialization ability and that the inducible heat shock response may be lost. However, more detailed experimentation will be required to understand the heat shock responses of Grylloblattodea. For example, research on the Antarctic midge, *Belgica antarctica* (Rinehart et al., 2006), has shown that the ability to exhibit an inducible heat shock response varies across larval and adult life stages, and in other insect species, it can be initiated at different durations following exposure to thermal stress (Storey & Storey, 2012).

### 4.2 Protein evolution and cold specialization

There is additional evidence that cold specialization has constrained thermal niche evolution in Grylloblattodea. Changes in physiological processes that maximize energy allocation can involve trade‐offs that are challenging to reverse (Angilletta Jr., Wilson, Navas, & James, 2003). Additionally, selection on protein function in cold environments leads to structural changes that impair function at other temperatures (Coquelle, Fioravanti, Weik, Vellieux, & Madern, 2007; Fields, 2001). We found evidence of positive selection on protein‐coding genes involved in trehalose transport, metabolic function, cellular respiration, oxygen reduction, oxidative stress response, and protein synthesis. Previous research has proposed that many of these molecular pathways related to survival in cold climates (Storey & Storey, 2012).

For example, a recent review of molecular adaptation to chill tolerance in insects (Overgaard & MacMillan, 2017) suggests that taxa should undergo structural changes in amino acid composition for genes encoding lipid membrane composition, osmolyte production (e.g., trehalose), and regulation of protein synthesis, in addition to selection on thermal sensitivity of key enzymes involved in cell homeostasis like gated ion channels and ion transporters. Cell membrane function and ion homeostasis, as well as the enhancement of antioxidant defenses and suppression of the apoptosis pathway, are generally viewed as key physiological requirements for cold tolerance in insects (Overgaard & MacMillan, 2017; Storey & Storey, 2012). Selection on protein‐coding genes in the Grylloblattodea trehalose pathway (*Tret1‐2*) is not surprising, given that trehalose production is known to protect cellular macromolecules and stabilize the membrane lipid bilayer in other cold‐specialized insects (Storey & Storey, 2012). A variety of techniques, including genetics and metabolomics, has confirmed that trehalose not only serves as a stress protectant, but also promotes starvation resistance (Hibshman et al., 2017). Furthermore, knock‐out studies in *D. melanogaster* have shown that trehalose is also required for body water homeostasis and desiccation tolerance in some insects (Yoshida, Matsuda, Kubo, & Nishimura, 2016).

We also found results indicating pervasive selection on mitochondrial function, including genes involved in cellular respiration, mitochondrial protein synthesis, protein folding, and autophagy, as well as oxidoreductase activity. This is noteworthy, as many freeze‐avoidant insects show reorganization of mitochondrial metabolism to maintain aerobic respiration at cold temperatures (Storey & Storey, 2012). Selection on these pathways extends beyond insects to other poikilotherms, as the extensive literature on stenothermal fish has shown similar requirements. Adaptive regulatory changes occur in pathways involving protein synthesis, protein folding, and protein degradation (especially via the ubiquitination pathway), as well as in pathways involving lipid metabolism, mitochondrial function, antioxidant response, anti‐apoptosis, and immunity (Logan & Buckley, 2015). In results that parallel ours, a recent study of cold specialization in alpine stick insects found selection on *sulfotransferase* as evidence for metabolic adaptation (Dennis, Dunning, Sinclair, & Buckley, 2015).

Do these molecular changes in protein‐coding genes imply that there are constraints that limit thermal niche evolution? Performance at cold temperature is predicted to require amino acid changes that influence thermal sensitivity and activity of proteins (Overgaard & MacMillan, 2017), and thus, the patterns of positive selection that we observe likely include trade‐offs with respect to protein function in warmer thermal niches. In stenothermal Antarctic fish, cold specialization has led to evolutionary losses of key proteins that likely impair physiological processes at warm temperatures (Beers & Jayasundara, 2015). Comparative analysis of some of our selected proteins in other cold‐specialized species suggests that this might be the case. Several candidate genes in Grylloblattodea have appeared in functional biochemical studies of cold adaptation in other organisms. For example, structural changes in *tubulin alpha‐1* have been linked to the efficiency of microtubule assembly in Antarctic fish and algae (Detrich, Parker, Williams, Nogales, & Downing, 2000; Willem et al., 1999), and their function is impaired at warm temperatures. Similarly, amino acid changes in *Peptide Transporter Family 1* appear to increase protein flexibility at subzero temperatures and enhance nutritional sequestration of animal protein in icefish (Rizzello et al., 2013), but to result instability at higher temperatures. Additional work is needed to understand if the amino acid changes we observe in Grylloblattodea specifically enhance protein function at cold temperatures.

### 4.3 Evolutionary diversification of Mantophasmatodea and Grylloblattodea

Our phylogenetic analyses of protein‐coding genes provide important insight into the evolutionary relationships of these poorly studied insect lineages. For Mantophasmatodea, the phylogenetic analyses suggest an early split into two major clades, corresponding to a range disjunction formed by an arid region separating northern and central Namibia from southern Namibia and South Africa (Eberhard et al., 2018). This biogeographic division is consistent with a previous phylogenetic analysis using peptidomics (Predel et al., 2012), and seen in other vertebrate and invertebrate groups (Griffin, 1998a, 1998b). Furthermore, our inferred relationships (Figures 1 and 2) are consistent with the peptidomics phylogeny, and largely consistent with a phylogeny based on mitochondrial data (Damgaard, Klass, Picker, & Buder, 2008; Dool, Künzel, Haase, Picker, & Eberhard, 2017). The mitochondrial phylogeny differs in the placement of *T. gladiator* as the most basal lineage (perhaps due to different taxon sampling), and placing Austrophasmatidae sp. 1 as sister to Austrophasmatidae sp. 2 rather than *K. biedouwense* in our analysis. Further work will be required to understand the source of the discrepancies with this mitochondrial dataset.

For the Grylloblattodea, the transcriptome data provide the first well‐supported phylogeny of relationships among genera, as earlier reconstructions resulted in considerable uncertainty in deeper nodes of the phylogeny (Jarvis & Whiting, 2006; Schoville et al., 2013). In the species tree analysis of our three datasets (Figure 1), the genus *Grylloblattella* from the Altai‐Sayan Mountains is placed as sister to North American *Grylloblatta*. While two of the three best ML trees of the concatenated amino acid data disagree (Figure 2), FcLM analysis suggests that a sister‐group relationship of *Grylloblattella* and *Grylloblatta* is in fact the preferred relationship. Such a relationship is not unwarranted, as disjunct patterns between the Altai‐Sayan Mountains and western North America are seen in other poorly dispersing species insects (Dudko, 2011), suggesting suitable habitat may have been more continuous between the two regions at an earlier period of time. Previous research has shown that *Grylloblatta* diversify during pronounced dry periods in the climate record (Schoville, Bougie, Dudko, & Medeiros, 2019), and so this disjunct distribution across the Northern Hemisphere may parallel patterns found in other Tertiary relicts whose distribution became highly fragmented with global dry spells (Hampe & Jump, 2011). Our phylogeny also confirms the distant relationship of *G. yezoensis* from other *Galloisiana* species (Schoville et al., 2013), rendering *Galloisiana* paraphyletic and suggesting this may be an undescribed genus (Schoville, 2014). There is, however, disagreement about the placement of this lineage as sister to *Grylloblattella* + *Grylloblatta* (Figure 1), or sister to *Galloisiana* + *Grylloblattina* (Figure 2; Figure S2). FcLM analysis of the concatenated data supports the latter placement. Further taxonomic sampling may improve estimates of these relationships. Finally, our results of relationships within *Grylloblatta* are consistent with a recent 322 gene nuclear phylogeny of 96 *Grylloblatta* samples (Schoville et al., 2019), with the exception that *G*. sp. "Sierra Buttes" is highly nested within California species in the transcriptome species tree (Figure 1).

Analysis of other candidate proteins may shed additional light into the diversification of Mantophasmatodea and Grylloblattodea. While the lineages occupy highly divergent habitat and thermal niche space, they are notable among Polyneopteran insects in sharing complete wing loss. It is unclear if winglessness is a synapomorphy, or an independently derived character state in both lineages (Eberhard et al., 2018). Though fully winged fossils have been assigned to Grylloblattodea (Storozhenko & Aristov, 2014; Yingying, Béthoux, Chungkun, & Dong, 2010), there is some controversy over the placement of these fossils as ancestors to extant Grylloblattodea due to the lack of diagnostic synapomorphies (Wipfler et al., 2014). It is therefore noteworthy then that one of the top candidate selection genes identified in Grylloblattodea, *protein lingerer*, is involved in developing wing tissues in *Drosophila* (Baumgartner, Stocker, & Hafen, 2013). Further work will be needed to understand if this is linked to winglessness in Grylloblattodea and provides evidence of independent wing loss relative to Mantophasmatodea. Another interesting result is the pattern of Grylloblattodea‐specific selection on multiple odorant‐binding proteins, which may signal unique chemosensory specializations (Robertson, 2019). For example, odorant‐binding proteins have been linked to adaptive shifts in food preferences in novel environments (Wada‐Katsumata, Robertson, Silverman, & Schal, 2018). It is possible, however, that some of these candidate genes have strongly divergent coding sequences due to pseudogenization. Pseudogenes can be expressed as RNA for multiple gene regulatory functions, including suppression of molecular pathways (Tutar, 2012). The development of genome resources in Grylloblattodea would aid in more detailed examination of these candidate selection genes.

### 4.4 Implications for conservation of Grylloblattodea

Relict species are often valued in conservation decision making due to their phylogenetic uniqueness, endemism and rarity (Winter et al., 2013). These species are also associated with greater extinction risk, as extinction risk appears to increase with taxon age (Warren et al., 2018). These species should warrant special protected status if they are exceptionally vulnerable to environmental change. Here, we showed that cold specialization in Grylloblattodea has been accompanied by molecular changes to core physiological processes that create evolutionary constraints. Species of Grylloblattodea persist in small patches of suitable habitat (Schoville et al., 2019), and because they are poorly dispersing and have narrow ecological niches, they are unlikely to have the capacity to move or evolve in response to the rapid rate of contemporary climate change (Moritz & Agudo, 2013). As a result, along with other cold‐specialized species that inhabit alpine and cave environments, they should be considered "exceptionally at risk" of decline under climate change projections (La Sorte & Jetz, 2010; Mammola, Goodacre, & Isaia, 2018; Ohlemüller et al., 2008). Much more effort needs to be made to understand their ecology and population dynamics to aid in conservation management (Schoville & Graening, 2013).

## AUTHOR CONTRIBUTIONS

SDS, BW, and SSi designed the project. SDS, BW, MB, RD, MJEB, SCK, PBF, and RM contributed samples. SDS and SCK performed the experiments. SDS, ZB, PCW, MV, and SSi analyzed data. SDS wrote the paper, with all authors providing critical feedback.

## Supporting information

Supplementary Material

## Data Availability

Raw sequence data and transcriptome assemblies have been deposited at the National Center for Biotechnology Information (sample numbers are listed in Table 1).
