# Supplementary material for: Comparative transcriptomics of ice‐crawlers demonstrates cold specialization constrains niche evolution in a relict lineage
Source: Evol Appl. 2020 Sep 11;14(2):360–82. doi: 10.1111/eva.13120 (PMC7896716; doi:10.1111/eva.13120)
Supplement: Supplementary file 1 — Supplementary Material [file EVA-14-360-s001.docx]

**Supplementary Table** **S1**. Accession numbers for samples sequenced by the 1K Insect Transcriptome Evolution TE (1KITE) project.

| **Taxon** | **1KITE lib ID** | **BioProject** | **BioSample Accession** | **Taxonomy ID** | **Experiment accession** | **Run accession** | **TSA accession** | **TSA Version** |
| --- | --- | --- | --- | --- | --- | --- | --- | --- |
| Austrophasmatidae sp. 1 (36) | RINSinlTBVRAAPEI-36 | 286659 | SAMN04005129 | 1661462 | SRX1178838 | SRR2230514 | GDYL00000000 | GDYL01000000 |
| Austrophasmatidae sp. 2 (72) | RINSinlTDBRAAPEI-72 | 286344 | SAMN04005128 | 1661462 | SRX1178837 | SRR2230513 | GDYW00000000 | GDYW01000000 |
| *Galloisiana sinensis* | INSlupTBQRAAPEI-39 | 286378 | SAMN04005163 | 1661829 | SRX1178872 | SRR2230548 | GDTV00000000 | GDTV01000000 |
| *Galloisiana* sp. ‘Joshin'etsu Kogen Highlands’ | INSfrgTAKRAAPEI-21 | 219560 | SAMN02047172 | 378494 | SRX314854 | SRR921600 | GAWN00000000 | GDVU01000000 |
| *Galloisiana* sp. ‘Kanto Mountains’ | INSodkTACRAAPEI-57 | 286377 | SAMN04005162 | 73580 | SRX1178871 | SRR2230547 | GDWI00000000 | GDWI01000000 |
| *Grylloblattella pravdini* | INSlupTAJRAAPEI-56 | 286381 | SAMN04005166 | 1661527 | SRX1178875 | SRR2230551 | GDWD00000000 | GDWD01000000 |
| *Grylloblattina djakonovi* | INSeqtTAJRAAPEI-35 | 286382 | SAMN04005167 | 308011 | SRX1178876 | SRR2230552 | GDUY00000000 | GDUY01000000 |
| *Mantophasma* sp. 1 | INShkeTCFRAAPEI-84 | 273040 | SAMN03339343 | 1603006 | SRX884530 | SRR1811976 | GDBV00000000 | GDBV01000000 |
| *Mantophasma* sp. 2 (109) | RINSinlTBERAAPEI-109 | 286402 | SAMN04005187 | 1661474 | SRX1178896 | SRR2230572 | GDZT00000000 | GDZT01000000 |
| *Pachyphasma brandbergense* | INSinlTAURAAPEI-41 | 286425 | SAMN04005210 | 1041430 | SRX1178919 | SRR2230595 | GDUW00000000 | GDUW01000000 |
| *Striatophasma naukluftense* | RINSinlTBMRAAPEI-19 | 286460 | SAMN04005246 | 1041429 | SRX1178955 | SRR2230631 | GDXW00000000 | GDXW01000000 |
| *Tyrannophasma gladiator* | INSinlTALRAAPEI-22 | 286472 | SAMN04005258 | 270861 | SRX1178967 | SRR2230643 | GDVU00000000 | GDVU01000000 |

**Supplementary Table** **S2**. Details on samples, level of replication, and acute thermal stress exposures for Mantophasmatodea and Grylloblattodea.

| **Taxon** | **Control Treatment** | **Heat Stress Treatment** | **Cold Stress Treatment** |
| --- | --- | --- | --- |
| *Grylloblattella pravdini* | Collected from field prior to RNA isolation:  Adult female  Adult female  Adult female | RNA isolation following 30min exposure to 25-30°C:  Adult female  Adult female  Adult female | RNA isolation following 30min exposure to -5°C:  Adult female  Adult female  Adult female |
| *Galloisiana yezoensis* | Collected from field prior to RNA isolation:  Adult female  Larvae 3^rd^ instar | RNA isolation following 30min exposure to 35°C:  Larvae 6^th^ instar  Larvae 4^th^ instar | RNA isolation following 30min exposure to 2.5°C:  Adult female  Larvae 3^rd^ instar |
| *Grylloblatta bifratrilecta* | Collected from field prior to RNA isolation:  Adult female  Adult female | RNA isolation following 30min exposure to 20°C:  Adult female  Adult female | RNA isolation following 30min exposure to -5°C:  Adult female  Adult female |
| *Grylloblatta* sp. ‘Lillburn Cave’ | Collected from field prior to RNA isolation:  Female  Male | RNA isolation following 30min exposure to 20°C:  Male  4 larvae, 2-3^rd^ instar | RNA isolation following 30min exposure to -5°C:  Male  Larvae 6^th^ instar |
| *Grylloblatta* sp. ‘Sierra Buttes’ | Collected from field prior to RNA isolation:  Larvae 5^th^ instar  Larvae 5^th^ instar | RNA isolation following 30min exposure to 20°C:  Larvae 5^th^ instar  Larvae 5^th^ instar | RNA isolation following 30min exposure to -5°C:  Larvae 5^th^ instar |
| *Grylloblatta gurneyi*: Lava Beds | Collected from field prior to RNA isolation:  Larvae 3rd instar | RNA isolation following 30min exposure to 20°C:  Larvae 3rd instar | n/a |
| *Grylloblatta gurneyi*: Mt. Shasta | Collected from field prior to RNA isolation:  Adult female | RNA isolation following 30min exposure to 20°C:  Larvae 4^th^ instar  Larvae 4^th^ instar | n/a |
| *Grylloblatta marmoreus* | Collected from field prior to RNA isolation:  Adult female  Adult female | RNA isolation following 30min exposure to 20°C:  Adult female | RNA isolation following 30min exposure to -5°C:  Adult female |
| *Grylloblatta* sp. ‘North Cascades’: Mt. Rainier | Collected from field prior to RNA isolation:  Adult female | RNA isolation following 30min exposure to 20°C:  Adult female | RNA isolation following 30min exposure to -5°C:  Adult female  Adult female |
| *Grylloblatta* sp. ‘North Cascades’: Whitechuck Mtn. | Collected from field prior to RNA isolation:  Adult female  Adult female | RNA isolation following 30min exposure to 20°C:  Adult female  Adult female | RNA isolation following 30min exposure to -5°C:  Adult female  Adult female |
| *Karoophasma biedouwense* | Maintained at constant temperature, ~20 °C in lab, >2wks prior to RNA isolation:  Adult female  Adult female  Adult female  Adult female  Adult female | RNA isolation following 30min exposure to 40.4 +/- 0.2 °C:  Adult male  Adult female  Adult female  Adult female  Adult female | RNA isolation following 30min exposure to -0.1 +/- 1.6 °C:  Adult female  Adult female  Adult female  Adult female  Adult female |

**Supplementary Table** **S3**. Selected groups for testing position of *Galloisiana yezoensis* to the other East Asian Grylloblattodea species.

| **Group** | **Taxonomic clade and geographical grouping** | **Species** |
| --- | --- | --- |
| G1 | Grylloblattodea, East Asia | *Galloisiana yezoensis* |
| G2 | Grylloblattodea, East Asia | *Grylloblattina djakanovi* |
| G2 | Grylloblattodea, East Asia | *Galloisiana sinensis* |
| G2 | Grylloblattodea, East Asia | *Galloisiana* sp. “Joshin’etsu-Kogen Highlands” |
| G2 | Grylloblattodea, East Asia | *Galloisiana* sp. “Kanto Mountains” |
| G3 | Grylloblattodea, East Asia | *Grylloblattella pravdini* |
| G4 | Grylloblattodea, North America | *Grylloblatta bifratrilecta* |
| G4 | Grylloblattodea, North America | *Grylloblatta chandleri* |
| G4 | Grylloblattodea, North America | *Grylloblatta gurneyi:* Lava Beds |
| G4 | Grylloblattodea, North America | *Grylloblatta gurneyi:* Mt. Shasta |
| G4 | Grylloblattodea, North America | *Grylloblatta* sp. ‘North Cascades’: Mt. Rainier |
| G4 | Grylloblattodea, North America | *Grylloblatta marmoreus* |
| G4 | Grylloblattodea, North America | *Grylloblatta* sp. ‘North Cascades’: Whitechuck Mtn. |
| G4 | Grylloblattodea, North America | *Grylloblatta* sp. ‘Sierra Buttes’ |
| G4 | Grylloblattodea, North America | *Grylloblatta* sp. ‘Trinity Alps’ |
| G4 | Grylloblattodea, North America | *Grylloblatta occidentalis* |
| G4 | Grylloblattodea, North America | *Grylloblatta* sp. ‘Lillburn Cave’ |

**Supplementary Table** **S4**. Selected groups for testing position of *Grylloblattella pravdini* to the other East Asian Grylloblattodea species.

| **Group** | **Taxonomic clade and geographical grouping** | **Species** |
| --- | --- | --- |
| G1 | Grylloblattodea, East Asia | *Galloisiana yezoensis* |
| G1 | Grylloblattodea, East Asia | *Grylloblattina djakanovi* |
| G1 | Grylloblattodea, East Asia | *Galloisiana sinensis* |
| G1 | Grylloblattodea, East Asia | *Galloisiana* sp. “Joshin’etsu-Kogen Highlands” |
| G1 | Grylloblattodea, East Asia | *Galloisiana* sp. “Kanto Mountains” |
| G2 | Grylloblattodea, East Asia | *Grylloblattella pravdini* |
| G3 | Grylloblattodea, North America | *Grylloblatta bifratrilecta* |
| G3 | Grylloblattodea, North America | *Grylloblatta chandleri* |
| G3 | Grylloblattodea, North America | *Grylloblatta gurneyi:* Lava Beds |
| G3 | Grylloblattodea, North America | *Grylloblatta gurneyi:* Mt. Shasta |
| G3 | Grylloblattodea, North America | *Grylloblatta* sp. ‘North Cascades’: Mt. Rainier |
| G3 | Grylloblattodea, North America | *Grylloblatta marmoreus* |
| G3 | Grylloblattodea, North America | *Grylloblatta* sp. ‘North Cascades’: Whitechuck Mtn. |
| G3 | Grylloblattodea, North America | *Grylloblatta* sp. ‘Sierra Buttes’ |
| G3 | Grylloblattodea, North America | *Grylloblatta* sp. ‘Trinity Alps’ |
| G3 | Grylloblattodea, North America | *Grylloblatta occidentalis* |
| G3 | Grylloblattodea, North America | *Grylloblatta* sp. ‘Lillburn Cave’ |
| G4 | Mantophasmatodea | Austrophasmatidae sp. 1 (36) |
| G4 | Mantophasmatodea | Austrophasmatidae sp. 2 (72) |
| G4 | Mantophasmatodea | *Karoophasma biedouwense* |
| G4 | Mantophasmatodea | *Mantophasma* sp. 1 |
| G4 | Mantophasmatodea | *Mantophasma* sp. 2 (109) |
| G4 | Mantophasmatodea | *Pachyphasma brandbergense* |
| G4 | Mantophasmatodea | *Striatophasma naukluftense* |
| G4 | Mantophasmatodea | *Tanzaniophasma* sp. 1 |
| **G4** | Mantophasmatodea | *Tyrannophasma gladiator* |

**Supplementary Table** **S5**. Transcriptome assembly statistics. BUSCO percentages are based on the insecta v9 reference database, with 1,668 genes.

| **Grylloblattodea** | **Assembly Size (base pairs)** | **# Contigs in Transcriptome** | **Median Contig Length (base pairs)** | **% Complete BUSCOs** | **% Complete and single-copy BUSCOs** | **% Complete and duplicated BUSCOs** | **% Fragmented BUSCOs** | **% Missing BUSCOs** |
| --- | --- | --- | --- | --- | --- | --- | --- | --- |
| *Galloisiana* sp. “Joshin’etsu-Kogen Highlands” | 85,162,438 | 188,251 | 34,203 | 81.60% | 76.20% | 5.40% | 15.10% | 3.30% |
| *Galloisiana* sp. “Kanto Mountains” | 57,944,662 | 148,096 | 35,453 | 58.40% | 54.90% | 3.50% | 29.10% | 12.50% |
| *Galloisiana sinensis* | 60,015,941 | 127,937 | 24,980 | 67.50% | 62.70% | 4.80% | 20.00% | 12.50% |
| *Galloisiana yezoensis* | 37,115,990 | 51,552 | 9,269 | 74.40% | 70.20% | 4.20% | 11.20% | 14.40% |
| *Grylloblatta bifratrilecta* | 98,733,517 | 153,024 | 27,691 | 84.50% | 63.90% | 20.60% | 10.00% | 5.50% |
| *Grylloblatta chandleri* | 19,171,509 | 32,308 | 6,895 | 50.20% | 49.40% | 0.80% | 14.00% | 35.80% |
| *Grylloblatta gurneyi:* Lava Beds | 27,449,803 | 42,965 | 8,671 | 59.80% | 58.80% | 1.00% | 12.60% | 27.60% |
| *Grylloblatta gurneyi:* Mt. Shasta | 33,959,511 | 50,361 | 9,716 | 65.60% | 63.90% | 1.70% | 11.80% | 22.60% |
| *Grylloblatta* sp. ‘Lillburn Cave’ | 34,663,352 | 50,872 | 9,070 | 75.50% | 73.80% | 1.70% | 12.40% | 12.10% |
| *Grylloblatta marmoreus* | 39,617,914 | 57,438 | 10,902 | 71.50% | 68.80% | 2.70% | 12.70% | 15.80% |
| *Grylloblatta* sp. ‘North Cascades’: Mt. Rainier | 41,706,267 | 66,469 | 13,009 | 70.20% | 68.90% | 1.30% | 13.50% | 16.30% |
| *Grylloblatta* sp. ‘North Cascades’: Whitechuck Mtn. | 49,184,589 | 69,880 | 12,812 | 76.90% | 72.10% | 4.80% | 10.90% | 12.20% |
| *Grylloblatta occidentalis* | 21,845,149 | 34,736 | 6,994 | 49.70% | 48.60% | 1.10% | 15.20% | 35.10% |
| *Grylloblatta* sp. ‘Sierra Buttes’ | 43,426,956 | 62,170 | 11,521 | 75.60% | 74.20% | 1.40% | 9.00% | 15.40% |
| *Grylloblatta* sp. ‘Trinity Alps’ | 12,533,572 | 23,242 | 5,255 | 36.20% | 35.50% | 0.70% | 17.30% | 46.50% |
| *Grylloblattella pravdini* | 50,516,662 | 70,697 | 13,240 | 74.20% | 65.20% | 9.00% | 11.30% | 14.50% |
| *Grylloblattina djakanovi* | 50,222,289 | 104,914 | 20,332 | 67.60% | 64.10% | 3.50% | 21.60% | 10.80% |
| **Mantophasmatodea** | | | | | | | | |
| Austrophasmatidae sp. 1 (36) | 22,135,761 | 37,754 | 7,781 | 39.90% | 36.40% | 2.90% | 21.00% | 39.70% |
| Austrophasmatidae sp. 2 (72) | 23,161,290 | 40,849 | 8,292 | 49.60% | 47.60% | 2.00% | 19.70% | 30.70% |
| *Karoophasma biedouwense* | 265,098,655 | 422,106 | 58,164 | 89.60% | 28.40% | 61.20% | 8.00% | 2.40% |
| *Mantophasma* sp. 1 | 49,168,831 | 110,487 | 22,836 | 53.50% | 50.40% | 3.10% | 26.10% | 20.40% |
| *Mantophasma* sp. 2 (109) | 21,535,003 | 26,163 | 5,363 | 56.40% | 53.40% | 3.00% | 15.10% | 28.50% |
| *Pachyphasma brandbergense* | 11,285,518 | 14,124 | 2,899 | 39.10% | 37.70% | 1.40% | 15.00% | 45.90% |
| *Striatophasma naukluftense* | 21,899,905 | 39,446 | 8,108 | 46.10% | 43.40% | 2.70% | 22.10% | 31.80% |
| *Tanzaniophasma* sp. 1 | 34,304,137 | 36,638 | 6,317 | 74.00% | 67.70% | 6.30% | 18.90% | 7.40% |
| *Tyrannophasma gladiator* | 27,868,721 | 44,807 | 8,294 | 58.30% | 54.70% | 3.60% | 17.90% | 23.80% |

**Supplementary Table** **S6**. Results of Four-cluster Likelihood Mapping (FcLM) and permutation tests to determine the support for the phylogenetic relationship of *Galloisiana yezoensis* to the other East Asian Grylloblattodea. T1 preferred in ML tree: 668,495 aa, 1,942 genes. G1: *Galloisiana yezoensis* (1), G2: Other East Asian Grylloblattodea spp. (4), G3: *Grylloblattella pravdini* (1), G4: North American *Grylloblatta* spp. (121). Unique quartets: 44.

| Topology | Original  (support in %) | Permutation I  (support in %) | Permutation II  (support in %) | Permutation III  (support in %) |
| --- | --- | --- | --- | --- |
| T1: G1G2\|G3G4 | 100 | 54.5 | 22.7 | 34.1 |
| T2: G1G3\|G2G4 | 0 | 22.7 | 50.0 | 25.0 |
| T3: G1G4\|G2G3 | 0 | 15.9 | 18.2 | 36.4 |
| T1T2: G1G2\|G3G4 or G1G3\|G2G4 | 0 | 2.3 | 4.5 | 2.3 |
| T1T3: G1G2\|G3G4 or G1G4\|G2G3 | 0 | 2.3 | 4.5 | 0 |
| T2T3: G1G3\|G2G4 or G1G4\|G2G3 | 0 | 2.3 | 0 | 2.3 |
| T*: G1G2\|G3G4 or G1G3\|G2G4 or G1G4\|G2G3 | 0 | 0 | 0 | 0 |

**Supplementary Table** **S7**. Results of Four-cluster Likelihood Mapping (FcLM) and permutation tests to determine the support for the phylogenetic relationship of *Grylloblattella pravdini* to the other East Asian Grylloblattodea. T1 preferred in ML tree: 705,274 aa, 2,041 genes. G1: *Galloisiana yezoensis* (5), G2: *Grylloblattella pravdini* (1), G3: North American *Grylloblatta* spp. (11), G4: Mantophasmatodea (12). Unique quartets: 495.

| Topology | Original  (support in %) | Permutation I  (support in %) | Permutation II  (support in %) | Permutation III  (support in %) |
| --- | --- | --- | --- | --- |
| T1: G1G2\|G3G4 | 5.3 | 24.0 | 26.5 | 30.9 |
| T2: G1G3\|G2G4 | 7.9 | 36.6 | 34.1 | 32.1 |
| T3: G1G4\|G2G3 | 86.9 | 34.1 | 34.1 | 30.5 |
| T1T2: G1G2\|G3G4 or G1G3\|G2G4 | 0 | 1.4 | 1.8 | 2.0 |
| T1T3: G1G2\|G3G4 or G1G4\|G2G3 | 0 | 1.4 | 2.0 | 2.2 |
| T2T3: G1G3\|G2G4 or G1G4\|G2G3 | 0 | 2.4 | 1.4 | 2.2 |
| T*: G1G2\|G3G4 or G1G3\|G2G4 or G1G4\|G2G3 | 0 | 0 | 0 | 0 |

**Supplementary Table** **S8**. Top differentially expressed genes (FDR <0.01) in the acute heat stress treatment of *Karoophasma biedouwense*. Log fold change is relative to the control samples.

| **Annotation** | **Transcript ID** | **log Fold Change in Heat Stress Samples** | ***p-value*** | **FDR** |
| --- | --- | --- | --- | --- |
| Heat shock protein 70 A2 | DN138673_c2_g3 | 8.499659923 | 7.91E-16 | 2.36E-11 |
| Heat shock protein 70 A1 | DN138673_c2_g2 | 7.724252719 | 2.57E-14 | 3.82E-10 |
| Heat shock protein 68 | DN132648_c1_g2 | 9.896158941 | 4.40E-14 | 4.37E-10 |
| Heat shock cognate 71 kDa protein | DN138673_c2_g1 | 6.949327997 | 4.36E-12 | 3.25E-08 |
| Unknown | DN137333_c1_g6 | 6.572554563 | 5.96E-08 | 0.000355087 |
| Unknown | DN133225_c0_g3 | 7.336749899 | 1.47E-07 | 0.000727507 |
| Heat shock-related 70 kDa protein 2 | DN132648_c0_g1 | 6.251236823 | 2.65E-07 | 0.001126694 |
| 16S ribosomal RNA gene | DN138258_c0_g1 | -6.441750809 | 4.84E-07 | 0.001778082 |
| Protein lethal(2)essential for life (hsp20) | DN135378_c2_g1 | 4.434071616 | 5.38E-07 | 0.001778082 |
| Unknown | DN139921_c2_g8 | 4.414323689 | 7.43E-07 | 0.002212384 |
| Unknown | DN136811_c0_g1 | 5.994828455 | 1.18E-06 | 0.00318119 |
| Protein lethal(2)essential for life (hsp20) | DN133517_c2_g2 | 3.903421125 | 1.31E-06 | 0.00319004 |
| Unknown | DN137526_c3_g4 | 8.753921991 | 1.39E-06 | 0.00319004 |
| heat shock protein 20.5 | DN133225_c0_g1 | 6.401996113 | 2.51E-06 | 0.005346955 |
| Unknown | DN129870_c0_g1 | 12.30791127 | 3.41E-06 | 0.006497514 |
| Unknown | DN110072_c0_g3 | 7.650623396 | 3.74E-06 | 0.006497514 |
| Alpha-crystallin A chain (hsp) | DN139921_c2_g10 | 4.21922635 | 3.87E-06 | 0.006497514 |
| Unknown | DN137333_c1_g2 | 5.685814829 | 4.13E-06 | 0.006497514 |
| 18S ribosomal RNA gene | DN119115_c0_g1 | -4.932471224 | 4.15E-06 | 0.006497514 |
| 18S ribosomal RNA gene | DN128879_c6_g5 | -4.95412807 | 4.41E-06 | 0.006570375 |
| Acyl-CoA Delta(11) desaturase | DN123028_c0_g1 | 4.338687703 | 5.52E-06 | 0.007825596 |
| Unknown | DN129465_c0_g3 | 6.151150154 | 6.63E-06 | 0.008445739 |
| Major heat shock 70 kDa protein Ab | DN123600_c0_g1 | 4.984200255 | 6.68E-06 | 0.008445739 |
| Unknown | DN141675_c13_g4 | 3.788316484 | 6.96E-06 | 0.008445739 |
| Protein TAR1 | DN133965_c12_g4 | -15.74751688 | 7.10E-06 | 0.008445739 |
| Unknown | DN129579_c0_g2 | 10.02671661 | 7.38E-06 | 0.008445739 |
| Unknown | DN138689_c0_g5 | 7.900893317 | 8.13E-06 | 0.008963621 |
| Unknown | DN133517_c2_g1 | 3.845174057 | 8.60E-06 | 0.009145279 |
| Unknown | DN133927_c3_g3 | -8.46360494 | 9.28E-06 | 0.009378184 |
| 16S ribosomal RNA gene | DN130822_c2_g6 | -13.49068136 | 9.45E-06 | 0.009378184 |
| 18S ribosomal RNA gene | DN138584_c1_g2 | -5.575782183 | 9.97E-06 | 0.009450546 |
| Protein TAR1 | DN136136_c2_g4 | -15.58501551 | 1.02E-05 | 0.009450546 |
| Unknown | DN137076_c3_g2 | 5.289315918 | 1.12E-05 | 0.009639849 |
| Putative uncharacterized protein ART2 | DN133770_c1_g7 | -13.56106693 | 1.14E-05 | 0.009639849 |
| 28S ribosomal RNA gene | DN137254_c68_g1 | -15.75010291 | 1.17E-05 | 0.009639849 |
| 28S ribosomal RNA gene | DN132605_c6_g8 | -14.72843895 | 1.18E-05 | 0.009639849 |
| 28S ribosomal RNA gene | DN126745_c0_g4 | -15.24870118 | 1.20E-05 | 0.009639849 |
| Unknown | DN140008_c2_g1 | 5.381563935 | 1.27E-05 | 0.009963861 |
| Unknown | DN131046_c0_g1 | 5.127455031 | 1.34E-05 | 0.010079859 |
| Unknown | DN130404_c0_g1 | 3.390331566 | 1.35E-05 | 0.010079859 |

**Supplementary Table** **S9**. Top differentially expressed genes (FDR <0.015) in the acute cold stress treatment of *Karoophasma biedouwense*. Log fold change is relative to the control samples.

| **Annotation** | **Transcript ID** | **log Fold Change in Cold Stress Samples** | ***p-value*** | **FDR** |
| --- | --- | --- | --- | --- |
| Unknown | DN129957_c6_g1 | 7.794913515 | 1.92E-09 | 4.90E-05 |
| 28S ribosomal RNA gene | DN137254_c74_g36 | 12.66436779 | 1.45E-06 | 0.01403407 |
| Unknown | DN135129_c0_g10 | -13.0607653 | 1.68E-06 | 0.01403407 |
| 16S RNA gene | DN132439_c6_g5 | -7.712778352 | 2.85E-06 | 0.01403407 |
| 28S ribosomal RNA gene | DN133798_c0_g1 | 15.30397532 | 3.57E-06 | 0.01403407 |
| 28S ribosomal RNA gene | DN137254_c74_g9 | 7.751387051 | 4.79E-06 | 0.01403407 |
| 28S ribosomal RNA gene | DN137254_c74_g13 | 7.236521198 | 5.32E-06 | 0.01403407 |
| Protein TAR1 | DN133965_c12_g4 | 15.78596039 | 6.12E-06 | 0.01403407 |
| 28S ribosomal RNA gene | DN137254_c74_g16 | 7.09843986 | 6.23E-06 | 0.01403407 |
| Unknown | DN138918_c3_g3 | 9.242196943 | 7.66E-06 | 0.01403407 |
| 18S ribosomal RNA gene | DN128675_c1_g6 | 13.00313212 | 7.87E-06 | 0.01403407 |
| Unknown | DN138918_c3_g2 | 8.967619647 | 8.70E-06 | 0.01403407 |
| Protein TAR1 | DN136136_c2_g4 | 15.62346164 | 8.78E-06 | 0.01403407 |
| Unknown | DN99719_c1_g1 | 8.361177771 | 9.64E-06 | 0.01403407 |
| 18S ribosomal RNA gene | DN130822_c2_g6 | 12.37633583 | 1.02E-05 | 0.01403407 |
| 28S ribosomal RNA gene | DN137254_c68_g1 | 15.78855262 | 1.02E-05 | 0.01403407 |
| 28S ribosomal RNA gene | DN132605_c6_g8 | 14.76687365 | 1.03E-05 | 0.01403407 |
| 28S ribosomal RNA gene | DN126745_c0_g4 | 15.2871454 | 1.04E-05 | 0.01403407 |
| Putative uncharacterized protein ART3 | DN129603_c1_g1 | 6.70091593 | 1.04E-05 | 0.01403407 |
| 16S RNA gene | DN109203_c0_g1 | 7.403067635 | 1.21E-05 | 0.014431139 |
| 28S ribosomal RNA gene | DN137254_c74_g7 | 8.556940561 | 1.24E-05 | 0.014431139 |
| 18S ribosomal RNA gene | DN131381_c0_g3 | 14.99864377 | 1.31E-05 | 0.014431139 |
| 28S ribosomal RNA gene | DN137254_c74_g2 | 6.657285369 | 1.35E-05 | 0.014431139 |
| Lysozyme | DN126765_c0_g2 | -10.34653309 | 1.36E-05 | 0.014431139 |
| 16S RNA gene | DN135129_c0_g11 | -11.61263876 | 1.44E-05 | 0.014431139 |
| Protein TAR1 | DN133965_c12_g1 | 13.04894153 | 1.47E-05 | 0.014431139 |
| Allergen Cr-PI | DN137867_c0_g3 | 6.718907005 | 1.61E-05 | 0.015155182 |

**Supplementary Table** **S10**. Top differentially expressed genes (FDR <0.02 or top five genes) in the acute heat stress treatment of Grylloblattodea species. Log fold change is relative to the control samples.

| **Annotation** | **Transcript ID** | **log Fold Change in Heat Stress Samples** | ***p-value*** | **FDR** |
| --- | --- | --- | --- | --- |
| *Galloisiana yezoensis* |  |  |  |  |
| Unknown | TR9011\|c12_g1 | 6.333877547 | 2.17E-10 | 8.76E-06 |
| Heat shock protein 70 B2 | TR6666\|c0_g1 | 7.917294124 | 9.35E-10 | 1.89E-05 |
| Unknown | TR6666\|c1_g1 | 7.312677175 | 2.58E-08 | 0.000347656 |
| Unknown | TR3006\|c42_g1 | -6.930202075 | 6.18E-08 | 0.000623018 |
| Unknown | TR2876\|c0_g1 | -4.86599271 | 1.64E-07 | 0.00132613 |
| Unknown | TR7828\|c27_g1 | 7.062867056 | 2.55E-07 | 0.001712264 |
| Unknown | TR3596\|c5_g1 | 3.294538275 | 6.71E-07 | 0.003866061 |
| Unknown | TR3237\|c0_g1 | 3.910074435 | 1.34E-06 | 0.006779554 |
| Unknown | TR16494\|c0_g1 | -12.32276744 | 2.36E-06 | 0.009761784 |
| Unknown | TR14165\|c3_g1 | 6.474798536 | 2.42E-06 | 0.009761784 |
| Unknown | TR13748\|c39_g1 | 2.205757508 | 3.92E-06 | 0.014369483 |
|  |  |  |  |  |
| *Grylloblattella pravdini* |  |  |  |  |
| Unknown | TR10340\|c0_g1 | -6.849999125 | 3.36E-08 | 0.001179018 |
| Eukaryotic translation initiation factor 4E-binding protein 2 | TR18949\|c2_g2 | -6.739101199 | 4.37E-08 | 0.001179018 |
| Unknown | TR17265\|c28_g1 | -9.200881531 | 1.32E-07 | 0.002373794 |
| Unknown | TR10427\|c36_g1 | 3.652082032 | 3.16E-07 | 0.004253839 |
| Unknown | TR105\|c0_g1 | 7.884591619 | 5.62E-07 | 0.005079122 |
| Unknown | TR15542\|c17_g10 | 6.451557879 | 5.65E-07 | 0.005079122 |
| Unknown | TR10427\|c9_g1 | 2.980792578 | 1.88E-06 | 0.014455144 |
| Unknown | TR19364\|c0_g1 | 8.702245886 | 2.90E-06 | 0.019532162 |
|  |  |  |  |  |
| *Grylloblatta bifratrilecta* |  |  |  |  |
| Unknown | TR8410/c0_g1 | -14.41997532 | 1.20E-05 | 0.004064069 |
| Unknown | TR24099/c13_g1 | -13.93820613 | 2.73E-05 | 0.004295781 |
| Unknown | TR24099/c14_g1 | -13.70539861 | 3.81E-05 | 0.004295781 |
| Unknown | TR2106/c0_g1 | -13.10512396 | 0.000111128 | 0.008168881 |
| Unknown | TR24099/c13_g2 | -13.08231328 | 0.000120841 | 0.008168881 |
| Unknown | TR21377/c0_g1 | -12.25654994 | 0.000223946 | 0.012615641 |
| Unknown | TR52697/c4_g1 | -12.2770242 | 0.000332403 | 0.016050296 |
| Unknown | TR31750/c5_g1 | -6.240219877 | 0.000447485 | 0.01890624 |
| Unknown | TR24099/c12_g2 | -12.02933843 | 0.000572632 | 0.020880002 |
| Unknown | TR52697/c4_g11 | -11.69887999 | 0.000617752 | 0.020880002 |
| Unknown | TR52697/c2_g1 | -6.129672992 | 0.001064596 | 0.032712128 |
|  |  |  |  |  |
| *Grylloblatta* sp. "Lillburn Cave" |  |  |  |  |
| A disintegrin and metalloproteinase with thrombospondin motifs 16 | TR11115\|c0_g1 | -6.82226079 | 5.51E-06 | 0.264433683 |
| Unknown | TR2599\|c25_g1 | -6.270867885 | 2.08E-05 | 0.420176877 |
| Vitellogenin-2 | TR12175\|c0_g1 | -11.20467315 | 3.48E-05 | 0.420176877 |
| Unknown | TR14359\|c17_g1 | 6.105891165 | 3.50E-05 | 0.420176877 |
| Unknown | TR10791\|c8_g1 | 7.212113725 | 6.48E-05 | 0.621654902 |
|  |  |  |  |  |
| *Grylloblatta* sp. "North Cascades" Whitechuck Mountain | |  |  |  |
| Unknown | TR2325\|c0_g1 | 6.187502455 | 9.35E-12 | 4.22E-07 |
| Epidermal growth factor-like protein 7 | TR10457\|c13_g1 | 6.260546358 | 3.11E-11 | 7.02E-07 |
| Unknown | TR6895\|c2_g1 | 4.918002456 | 8.51E-11 | 1.28E-06 |
| Epidermal growth factor-like protein 8 | TR10457\|c3_g1 | 5.623882535 | 1.89E-10 | 2.13E-06 |
| Unknown | TR15940\|c0_g1 | 7.534325935 | 2.89E-10 | 2.61E-06 |
| Unknown | TR4197\|c9_g1 | 6.33355856 | 4.64E-10 | 3.50E-06 |
| Unknown | TR15940\|c1_g1 | 7.384876999 | 8.66E-10 | 5.59E-06 |
| Full=Protocadherin Fat 1 | TR10457\|c3_g2 | 5.675844993 | 4.61E-08 | 0.000260184 |
| Stearoyl-CoA desaturase 5 | TR11715\|c11_g1 | 4.190481065 | 6.60E-08 | 0.000330994 |
| Unknown | TR10870\|c10_g1 | 6.710896832 | 8.12E-08 | 0.000334088 |
| RNA-directed RNA polymerase L | TR8329\|c3_g1 | -7.567591628 | 8.14E-08 | 0.000334088 |
| Unknown | TR8329\|c1_g1 | -6.772050356 | 1.64E-07 | 0.00061757 |
| Serine protease nudel | TR1296\|c8_g1 | 4.660356816 | 6.19E-07 | 0.002150116 |
| Nose resistant to fluoxetine protein 6 | TR10725\|c19_g1 | 4.543728297 | 8.56E-07 | 0.002761054 |
| Unknown | TR13835\|c7_g1 | 4.154692829 | 9.43E-07 | 0.002838982 |
| Unknown | TR8329\|c2_g1 | -6.41257951 | 1.03E-06 | 0.002920786 |
| Unknown | TR3887\|c3_g4 | -4.34192974 | 2.26E-06 | 0.005808841 |
| Unknown | TR12118\|c5_g1 | 6.336872296 | 2.32E-06 | 0.005808841 |
| Unknown | TR7077\|c0_g1 | -12.089879 | 4.76E-06 | 0.011309299 |
| Unknown | TR594\|c5_g1 | 3.836637696 | 6.47E-06 | 0.014605224 |

**Supplementary Table** **S11**. Top differentially expressed genes (FDR <0.015 or top five genes) in the acute cold stress treatment of Grylloblattodea species. Log fold change is relative to the control samples.

| **Annotation** | **Transcript ID** | **log Fold Change in Cold Stress Samples** | ***p-value*** | **FDR** |
| --- | --- | --- | --- | --- |
| *Galloisiana yezoensis* |  |  |  |  |
| Unknown | TR2876\|c0_g1 | 8.035462848 | 8.62E-10 | 3.83E-05 |
| Unknown | TR5554\|c14_g1 | -4.147364632 | 3.60E-09 | 8.00E-05 |
| Unknown | TR73\|c1_g1 | -6.650518216 | 1.99E-07 | 0.00295495 |
| Unknown | TR16494\|c0_g1 | 13.23113297 | 7.96E-07 | 0.008846229 |
| Transferrin | TR16209\|c0_g1 | 2.784207859 | 1.23E-06 | 0.010908649 |
| Unknown | TR10679\|c1_g1 | -3.165845374 | 2.57E-06 | 0.019035522 |
| Unknown | TR64\|c3_g1 | 2.685562244 | 7.01E-06 | 0.0435455 |
| Unknown | TR16970\|c9_g1 | 6.197468059 | 7.84E-06 | 0.0435455 |
|  |  |  |  |  |
| *Grylloblattella pravdini* |  |  |  |  |
| Unknown | TR6609\|c1_g1 | -5.148603543 | 6.04E-17 | 2.68E-12 |
| Lysozyme | TR18786\|c0_g1 | -6.390516691 | 4.25E-12 | 9.43E-08 |
| Unknown | TR7592\|c0_g1 | 3.153323177 | 1.35E-08 | 0.00019931 |
| Unknown | TR4040\|c0_g1 | -5.91472525 | 6.03E-07 | 0.005685327 |
| Unknown | TR37\|c17_g1 | -2.96213142 | 6.41E-07 | 0.005685327 |
| Alpha-N-acetylgalactosaminidase | TR1913\|c7_g1 | -2.820581599 | 9.57E-07 | 0.007077649 |
| Unknown | TR13161\|c8_g1 | 3.143446817 | 1.24E-06 | 0.007882798 |
| Lipase 3 | TR6661\|c1_g1 | -3.515626612 | 1.99E-06 | 0.01103464 |
| Unknown | TR7561\|c0_g1 | 3.545298848 | 2.57E-06 | 0.012668811 |
|  |  |  |  |  |
| *Grylloblatta bifratrilecta* |  |  |  |  |
| Unknown | TR2106/c0_g1 | 12.93885158 | 9.40E-05 | 0.032241926 |
| Unknown | TR52697/c4_g1 | 12.11076809 | 0.000414982 | 0.071169399 |
| Unknown | TR24099/c13_g2 | 8.687750913 | 0.001459846 | 0.112207841 |
| Unknown | TR24099/c12_g2 | 11.8630939 | 0.00151426 | 0.112207841 |
| Unknown | TR49583/c0_g1 | -12.49687669 | 0.001758856 | 0.112207841 |
|  |  |  |  |  |
| *Grylloblatta* sp. "Lillburn Cave" | |  |  |  |
| Probable RNA-directed DNA polymerase from transposon BS | TR9263\|c2_g2 | 6.764661433 | 2.36E-06 | 0.065728533 |
| A disintegrin and metalloproteinase with thrombospondin motifs 16 | TR11115\|c0_g1 | 6.815971271 | 2.81E-06 | 0.065728533 |
| Unknown | TR5753\|c44_g1 | -6.662734538 | 4.11E-06 | 0.065728533 |
| Unknown | TR11115\|c1_g1 | 6.336271432 | 7.13E-06 | 0.085472365 |
| Ras-related protein Rab-14 | TR213\|c10_g3 | -6.111946316 | 2.01E-05 | 0.17929993 |
|  |  |  |  |  |
| *Grylloblatta* sp. "North Cascades" Whitechuck Mountain | |  |  |  |
| Unknown | TR10032\|c0_g2 | 7.565617854 | 3.08E-15 | 1.70E-10 |
| Unknown | TR13193\|c0_g1 | -8.705185688 | 6.66E-11 | 1.83E-06 |
| Unknown | TR7403\|c0_g1 | -4.149074707 | 8.32E-10 | 1.16E-05 |
| Unknown | TR13467\|c6_g1 | 4.365391811 | 8.45E-10 | 1.16E-05 |
| Cytochrome c oxidase subunit 3 | TR8581\|c0_g1 | -5.803433461 | 6.77E-09 | 6.48E-05 |
| Apolipoprotein D | TR9159\|c0_g1 | 5.787864351 | 7.06E-09 | 6.48E-05 |
| Elongation of very long chain fatty acids protein 4 | TR13467\|c5_g1 | 4.749122634 | 1.24E-08 | 9.73E-05 |
| Unknown | TR13193\|c1_g1 | -7.928822227 | 1.53E-08 | 0.000105504 |
| Unknown | TR14318\|c22_g1 | -7.467211766 | 2.51E-08 | 0.000147298 |
| Peroxidase | TR1234\|c0_g1 | -3.978476126 | 2.68E-08 | 0.000147298 |
| Unknown | TR2981\|c1_g2 | 7.194745021 | 4.20E-08 | 0.000209826 |
| Unknown | TR10725\|c27_g1 | -4.561548464 | 8.62E-08 | 0.00039515 |
| Endocuticle structural glycoprotein SgAbd-8 | TR15268\|c14_g1 | -3.692662088 | 1.03E-07 | 0.000434474 |
| Epidermal growth factor-like protein 7 | TR10457\|c13_g1 | -5.74953131 | 1.21E-07 | 0.000474245 |
| Cytochrome c oxidase subunit 1 | TR2750\|c1_g1 | -5.191323041 | 1.38E-07 | 0.000507091 |
| 40S ribosomal protein S3 | TR14492\|c3_g1 | 4.189666976 | 1.94E-07 | 0.000661625 |
| Cytochrome c oxidase subunit 2 | TR9320\|c0_g1 | -5.717534486 | 2.04E-07 | 0.000661625 |
| Unknown | TR13726\|c0_g1 | 4.500760147 | 2.19E-07 | 0.000668976 |
| Unknown | TR54\|c14_g1 | 6.553372484 | 2.57E-07 | 0.0007079 |
| Probable RNA-directed DNA polymerase from transposon BS | TR6317\|c5_g2 | 6.553372484 | 2.57E-07 | 0.0007079 |
| NADH-ubiquinone oxidoreductase chain 1 | TR9994\|c0_g1 | -5.984419658 | 2.92E-07 | 0.000763755 |
| Cytochrome b | TR13327\|c0_g1 | -5.646927814 | 3.49E-07 | 0.000839373 |
| Unknown | TR5623\|c24_g1 | -5.027516929 | 3.51E-07 | 0.000839373 |
| Unknown | TR15732\|c2_g1 | -4.661137735 | 3.67E-07 | 0.000840507 |
| Troponin C | TR6010\|c0_g1 | -2.788817417 | 3.90E-07 | 0.000858371 |
| Unknown | TR12274\|c2_g2 | -6.460709014 | 6.64E-07 | 0.001384501 |
| Unknown | TR14958\|c24_g1 | 2.887271162 | 6.98E-07 | 0.001384501 |
| Unknown | TR6895\|c2_g1 | -4.254013235 | 7.05E-07 | 0.001384501 |
| Unknown | TR7578\|c4_g1 | -3.415579311 | 8.07E-07 | 0.001530821 |
| Nose resistant to fluoxetine protein 6 | TR10725\|c19_g1 | -4.920073097 | 8.79E-07 | 0.001612515 |
| Biogenesis of lysosome-related organelles complex 1 subunit 2 | TR7755\|c5_g2 | -6.395346113 | 1.02E-06 | 0.001752766 |
| Epidermal growth factor-like protein 8 | TR10457\|c3_g1 | -5.089708322 | 1.02E-06 | 0.001752766 |
| Unknown | TR175\|c0_g1 | -4.423393181 | 1.26E-06 | 0.002106411 |
| Unknown | TR12118\|c5_g1 | -6.532032874 | 1.40E-06 | 0.002272165 |
| Unknown | TR8676\|c32_g1 | 3.696003832 | 1.46E-06 | 0.002288209 |
| Unknown | TR6698\|c10_g1 | -4.122896927 | 1.92E-06 | 0.002934191 |
| Unknown | TR15268\|c30_g1 | -4.651167587 | 2.08E-06 | 0.003074984 |
| Unknown | TR4657\|c0_g1 | -4.62598956 | 2.12E-06 | 0.003074984 |
| Unknown | TR15268\|c3_g1 | -3.428715471 | 2.37E-06 | 0.003343241 |
| Unknown | TR2325\|c0_g1 | -5.32132189 | 2.61E-06 | 0.003588808 |
| ATP synthase subunit a | TR6299\|c0_g1 | -5.665391398 | 2.70E-06 | 0.003627621 |
| Cytochrome c oxidase subunit 1 | TR2750\|c2_g1 | -5.217357837 | 3.30E-06 | 0.004328643 |
| Unknown | TR9456\|c5_g1 | -6.45259067 | 3.50E-06 | 0.004476579 |
| Unknown | TR7077\|c0_g1 | 12.11285665 | 3.59E-06 | 0.004492083 |
| Unknown | TR10546\|c0_g1 | 3.556382178 | 4.10E-06 | 0.004965616 |
| Unknown | TR5431\|c0_g1 | -6.572512064 | 4.15E-06 | 0.004965616 |
| Unknown | TR10546\|c18_g1 | 3.955841931 | 6.21E-06 | 0.007265414 |
| Unknown | TR273\|c12_g1 | 6.425799041 | 7.11E-06 | 0.008144546 |
| Protocadherin Fat 1 | TR10457\|c3_g2 | -5.28850778 | 8.20E-06 | 0.009209697 |
| NADH-ubiquinone oxidoreductase chain 3 | TR6332\|c0_g1 | -7.720931387 | 1.03E-05 | 0.011318323 |
| NADH-ubiquinone oxidoreductase chain 4 | TR14617\|c0_g1 | -6.839085487 | 1.08E-05 | 0.011634185 |
| Cytochrome P450 4g15 | TR16118\|c0_g1 | 3.823944871 | 1.15E-05 | 0.011836313 |
| Acyl-CoA synthetase short-chain family member 3, mitochondrial | TR10546\|c4_g1 | 4.112958381 | 1.16E-05 | 0.011836313 |
| Probable RNA-directed DNA polymerase from transposon BS | TR6317\|c5_g1 | -4.061931138 | 1.16E-05 | 0.011836313 |
| Unknown | TR1161\|c0_g1 | -6.870146675 | 1.21E-05 | 0.012149242 |
| Unknown | TR9895\|c0_g1 | -2.888379745 | 1.40E-05 | 0.013595368 |
| Unknown | TR9730\|c1_g1 | 2.750764384 | 1.42E-05 | 0.013595368 |
| Unknown | TR1717\|c0_g1 | -4.184894225 | 1.43E-05 | 0.013595368 |
| Unknown | TR9456\|c21_g1 | -6.171533549 | 1.54E-05 | 0.014371405 |
| Unknown | TR1161\|c1_g1 | -6.723525958 | 1.64E-05 | 0.014993936 |

**Supplementary Table** **S12**. Results of the test for enrichment of gene ontology categories following the acute temperature stress exposure. Only samples with biological replicates for each treatment are included. Annotation terms with a notable relationship to temperature stress responses or cold specialization are in bold.

| **Species** | **GO Term** | **Enriched *p-value*** | **Direction in Control Sample** | **# DE genes** | **Total # genes** | **Annotation** | **Genes** |
| --- | --- | --- | --- | --- | --- | --- | --- |
| *Karoophasma biedouwense* | | |  |  |  |  |  |
| Control vs Heat | |  |  |  |  |  |  |
|  | GO:0005739 | 0.035991 | Up | 3 | 757 | CC mitochondrion |  |
|  | GO:0009408 | 1.98E-08 | Down | 5 | 84 | **BP response to heat** | DN123600_c0_g1: HSP71 Major heat shock 70 kDa protein Ab; DN133517_c2_g2: L2EFL Protein lethal(2)essential for life; DN135378_c2_g1: L2EFL Protein lethal(2)essential for life; DN137076_c3_g2: L2EFL Protein lethal(2)essential for life; DN138673_c2_g3: HSP71 Heat shock protein 70 A1 |
|  | GO:0009266 | 1.74E-07 | Down | 5 | 132 | **BP response to temperature stimulus** | DN123600_c0_g1: HSP71 Major heat shock 70 kDa protein Ab; DN133517_c2_g2: L2EFL Protein lethal(2)essential for life; DN135378_c2_g1: L2EFL Protein lethal(2)essential for life; DN137076_c3_g2: L2EFL Protein lethal(2)essential for life; DN138673_c2_g3: HSP71 Heat shock protein 70 A1 |
|  | GO:0010998 | 7.97E-07 | Down | 3 | 14 | BP regulation of translational initiation by eIF2 alpha phosphorylation | |
|  | GO:0043558 | 7.97E-07 | Down | 3 | 14 | **BP regulation of translational initiation in response to stress** | DN133517_c2_g2: L2EFL Protein lethal(2)essential for life; DN135378_c2_g1: L2EFL Protein lethal(2)essential for life; DN137076_c3_g2: L2EFL Protein lethal(2)essential for life |
|  | GO:0043555 | 1.09E-06 | Down | 3 | 17 | **BP regulation of translation in response to stress** | DN133517_c2_g2: L2EFL Protein lethal(2)essential for life; DN135378_c2_g1: L2EFL Protein lethal(2)essential for life; DN137076_c3_g2: L2EFL Protein lethal(2)essential for life |
|  | GO:0051082 | 1.09E-06 | Down | 4 | 77 | **MF unfolded protein binding** | DN133517_c2_g2: L2EFL Protein lethal(2)essential for life; DN135378_c2_g1: L2EFL Protein lethal(2)essential for life; DN137076_c3_g2: L2EFL Protein lethal(2)essential for life; DN138673_c2_g2: HSP68 Heat shock protein 68 |
|  | GO:0042026 | 2.35E-06 | Down | 3 | 22 | **BP protein refolding** | DN133517_c2_g2: L2EFL Protein lethal(2)essential for life; DN135378_c2_g1: L2EFL Protein lethal(2)essential for life; DN137076_c3_g2: L2EFL Protein lethal(2)essential for life |
|  | GO:0035079 | 7.37E-06 | Down | 2 | 4 | BP polytene chromosome puffing |  |
|  | GO:0035080 | 7.37E-06 | Down | 2 | 4 | **BP heat shock-mediated polytene chromosome puffing** | DN123600_c0_g1: HSP71 Major heat shock 70 kDa protein Ab; DN138673_c2_g3: HSP71 Heat shock protein 70 A1 |
|  | GO:0006457 | 7.38E-06 | Down | 4 | 135 | **BP protein folding** | DN133517_c2_g2: L2EFL Protein lethal(2)essential for life; DN135378_c2_g1: L2EFL Protein lethal(2)essential for life; DN137076_c3_g2: L2EFL Protein lethal(2)essential for life; DN138673_c2_g2: HSP68 Heat shock protein 68 |
|  | GO:0061077 | 1.36E-05 | Down | 3 | 42 | **BP chaperone-mediated protein folding** | DN133517_c2_g2: L2EFL Protein lethal(2)essential for life; DN135378_c2_g1: L2EFL Protein lethal(2)essential for life; DN137076_c3_g2: L2EFL Protein lethal(2)essential for life |
|  | GO:0006446 | 2.11E-05 | Down | 3 | 48 | BP regulation of translational initiation |  |
|  | GO:0009628 | 9.99E-05 | Down | 5 | 516 | **BP response to abiotic stimulus** | DN123600_c0_g1: HSP71 Major heat shock 70 kDa protein Ab; DN133517_c2_g2: L2EFL Protein lethal(2)essential for life; DN135378_c2_g1: L2EFL Protein lethal(2)essential for life; DN137076_c3_g2: L2EFL Protein lethal(2)essential for life; DN138673_c2_g3: HSP71 Heat shock protein 70 A1 |
|  | GO:0045214 | 0.0001183 | Down | 3 | 86 | BP sarcomere organization |  |
|  | GO:0030018 | 0.0001461 | Down | 3 | 96 | CC Z disc |  |
|  | GO:0006986 | 0.0001985 | Down | 2 | 28 | BP response to unfolded protein |  |
|  | GO:0035966 | 0.0002679 | Down | 2 | 32 | BP response to topologically incorrect protein |  |
|  | GO:0034605 | 0.0002742 | Down | 2 | 27 | **BP cellular response to heat** | DN123600_c0_g1: HSP71 Major heat shock 70 kDa protein Ab; DN138673_c2_g3: HSP71 Heat shock protein 70 A1 |
|  | GO:0031032 | 0.0003031 | Down | 3 | 125 | BP actomyosin structure organization |  |
|  | GO:0005524 | 0.0003495 | Down | 6 | 1062 | MF ATP binding |  |
|  | GO:0032559 | 0.0003706 | Down | 6 | 1075 | MF adenyl ribonucleotide binding |  |
|  | GO:0030554 | 0.0003742 | Down | 6 | 1078 | MF adenyl nucleotide binding |  |
|  | GO:0006417 | 0.0005781 | Down | 3 | 171 | BP regulation of translation |  |
|  | GO:0033554 | 0.0006379 | Down | 5 | 774 | **BP cellular response to stress** | DN123600_c0_g1: HSP71 Major heat shock 70 kDa protein Ab; DN133517_c2_g2: L2EFL Protein lethal(2)essential for life; DN135378_c2_g1: L2EFL Protein lethal(2)essential for life; DN137076_c3_g2: L2EFL Protein lethal(2)essential for life; DN138673_c2_g3: HSP71 Heat shock protein 70 A1 |
|  | GO:0044449 | 0.0007227 | Down | 3 | 164 | CC contractile fiber part |  |
|  | GO:0035639 | 0.0009046 | Down | 6 | 1259 | MF purine ribonucleoside triphosphate binding |  |
|  | GO:0032550 | 0.0009219 | Down | 6 | 1263 | MF purine ribonucleoside binding |  |
|  | GO:0001883 | 0.000928 | Down | 6 | 1264 | MF purine nucleoside binding |  |
|  | GO:0032549 | 0.0009488 | Down | 6 | 1269 | MF ribonucleoside binding |  |
|  | GO:0032555 | 0.0009591 | Down | 6 | 1274 | MF purine ribonucleotide binding |  |
|  | GO:0001882 | 0.0009675 | Down | 6 | 1273 | MF nucleoside binding |  |
|  | GO:0017076 | 0.0009693 | Down | 6 | 1278 | MF purine nucleotide binding |  |
|  | GO:0032553 | 0.0009985 | Down | 6 | 1286 | MF ribonucleotide binding |  |
|  | GO:0006950 | 0.0012697 | Down | 6 | 1343 | **BP response to stress** | DN123600_c0_g1: HSP71 Major heat shock 70 kDa protein Ab; DN133517_c2_g2: L2EFL Protein lethal(2)essential for life; DN135378_c2_g1: L2EFL Protein lethal(2)essential for life; DN137076_c3_g2: L2EFL Protein lethal(2)essential for life; DN138673_c2_g3: HSP71 Heat shock protein 70 A1 |
|  | GO:0030036 | 0.0014411 | Down | 3 | 233 | BP actin cytoskeleton organization |  |
|  | GO:0036128 | 0.0015242 | Down | 1 | 1 | CC CatSper complex |  |
|  | GO:0030029 | 0.0018223 | Down | 3 | 256 | BP actin filament-based process |  |
|  | GO:0097367 | 0.0020099 | Down | 6 | 1468 | MF carbohydrate derivative binding |  |
|  | GO:0006468 | 0.0021114 | Down | 3 | 292 | BP protein phosphorylation |  |
|  | GO:0000166 | 0.0022877 | Down | 6 | 1500 | MF nucleotide binding |  |
|  | GO:1901265 | 0.0022877 | Down | 6 | 1500 | MF nucleoside phosphate binding |  |
|  | GO:0048471 | 0.0024604 | Down | 3 | 297 | CC perinuclear region of cytoplasm |  |
|  | GO:1902589 | 0.0025377 | Down | 5 | 1061 | BP single-organism organelle organization |  |
|  | GO:0051716 | 0.0025976 | Down | 5 | 1058 | **BP cellular response to stimulus** | DN123600_c0_g1: HSP71 Major heat shock 70 kDa protein Ab; DN133517_c2_g2: L2EFL Protein lethal(2)essential for life; DN135378_c2_g1: L2EFL Protein lethal(2)essential for life; DN137076_c3_g2: L2EFL Protein lethal(2)essential for life; DN138673_c2_g3: HSP71 Heat shock protein 70 A1 |
|  | GO:0042810 | 0.0035345 | Down | 1 | 3 | BP pheromone metabolic process |  |
|  | GO:0042811 | 0.0035345 | Down | 1 | 3 | BP pheromone biosynthetic process |  |
|  | GO:0036094 | 0.0036886 | Down | 6 | 1640 | MF small molecule binding |  |
|  | GO:0072687 | 0.0037392 | Down | 1 | 4 | CC meiotic spindle |  |
|  | GO:0001666 | 0.0038481 | Down | 2 | 103 | BP response to hypoxia |  |
|  | GO:0033142 | 0.003858 | Down | 1 | 3 | MF progesterone receptor binding |  |
|  | GO:0010608 | 0.0041666 | Down | 3 | 354 | BP posttranscriptional regulation of gene expression |  |
|  | GO:0036293 | 0.0041842 | Down | 2 | 108 | BP response to decreased oxygen levels |  |
|  | GO:0043168 | 0.0043539 | Down | 6 | 1724 | MF anion binding |  |
|  | GO:0016310 | 0.0044403 | Down | 3 | 378 | BP phosphorylation |  |
|  | GO:0070482 | 0.0048313 | Down | 2 | 115 | BP response to oxygen levels |  |
|  | GO:0007275 | 0.0050685 | Down | 3 | 347 | BP multicellular organism development |  |
|  | GO:0005891 | 0.0062205 | Down | 1 | 7 | CC voltage-gated calcium channel complex |  |
|  | GO:0017105 | 0.0064673 | Down | 1 | 6 | MF acyl-CoA delta11-desaturase activity |  |
|  | GO:0032570 | 0.0077319 | Down | 1 | 7 | BP response to progesterone |  |
|  | GO:0044767 | 0.0080078 | Down | 7 | 2551 | BP single-organism developmental process |  |
|  | GO:0000902 | 0.0080623 | Down | 2 | 152 | BP cell morphogenesis |  |
|  | GO:0005212 | 0.0093846 | Down | 1 | 7 | MF structural constituent of eye lens |  |
|  | GO:0006996 | 0.0095882 | Down | 5 | 1460 | BP organelle organization |  |
|  | GO:0032502 | 0.0101269 | Down | 7 | 2666 | BP developmental process |  |
|  | GO:0050896 | 0.0103076 | Down | 6 | 2040 | BP response to stimulus |  |
|  | GO:0034704 | 0.0117979 | Down | 1 | 13 | CC calcium channel complex |  |
|  | GO:0007010 | 0.0118702 | Down | 3 | 509 | BP cytoskeleton organization |  |
|  | GO:0016215 | 0.0128932 | Down | 1 | 11 | MF acyl-CoA desaturase activity |  |
|  | GO:0016717 | 0.0128932 | Down | 1 | 11 | MF oxidoreductase activity, acting on paired donors, with oxidation of a pair of donors resulting in the reduction of molecular oxygen to two molecules of water | |
|  | GO:0051276 | 0.0168636 | Down | 2 | 228 | BP chromosome organization |  |
|  | GO:0043167 | 0.0238522 | Down | 8 | 3757 | MF ion binding |  |
|  | GO:0051219 | 0.0246738 | Down | 1 | 30 | MF phosphoprotein binding |  |
|  | GO:0007369 | 0.0256512 | Down | 1 | 31 | BP gastrulation |  |
|  | GO:0034703 | 0.0281033 | Down | 1 | 33 | CC cation channel complex |  |
|  | GO:0044550 | 0.0287578 | Down | 1 | 29 | BP secondary metabolite biosynthetic process |  |
|  | GO:0048546 | 0.0292423 | Down | 1 | 36 | BP digestive tract morphogenesis |  |
|  | GO:0016043 | 0.0301927 | Down | 6 | 2637 | BP cellular component organization |  |
|  | GO:0035258 | 0.0323924 | Down | 1 | 34 | MF steroid hormone receptor binding |  |
|  | GO:0071840 | 0.0324385 | Down | 6 | 2679 | BP cellular component organization or biogenesis |  |
|  | GO:0009409 | 0.0324622 | Down | 1 | 31 | BP response to cold |  |
|  | GO:0032268 | 0.0333137 | Down | 3 | 797 | BP regulation of cellular protein metabolic process |  |
|  | GO:0005623 | 0.038201 | Down | 1 | 43 | CC cell |  |
|  | GO:0051246 | 0.0454968 | Down | 3 | 902 | BP regulation of protein metabolic process |  |
|  | GO:0006796 | 0.0456884 | Down | 3 | 887 | BP phosphate-containing compound metabolic process |  |
|  | GO:0042446 | 0.0462826 | Down | 1 | 44 | BP hormone biosynthetic process |  |
|  | GO:0048568 | 0.0469412 | Down | 1 | 48 | BP embryonic organ development |  |
|  | GO:0005488 | 0.0480177 | Down | 11 | 6775 | MF binding |  |
|  | GO:0035257 | 0.0483409 | Down | 1 | 52 | MF nuclear hormone receptor binding |  |
|  | GO:0051427 | 0.0489966 | Down | 1 | 53 | MF hormone receptor binding |  |
| Control vs. Cold | |  |  |  |  |  |  |
|  | Nothing |  |  |  |  |  |  |
|  |  |  |  |  |  |  |  |
| *Galloisiana yezoensis* | |  |  |  |  |  |  |
| Control vs. Heat | |  |  |  |  |  |  |
|  | GO:0042302 | 0.0168455 | Down | 1 | 64 | MF structural constituent of cuticle |  |
| Control vs. Cold | |  |  |  |  |  |  |
|  | Nothing |  |  |  |  |  |  |
|  |  |  |  |  |  |  |  |
| *Grylloblattella pravdini* | |  |  |  |  |  |  |
| Control vs Heat | |  |  |  |  |  |  |
|  | GO:0008190 | 0.0001968 | Up | 1 | 2 | MF eukaryotic initiation factor 4E binding |  |
|  | GO:0019933 | 0.0007086 | Up | 1 | 11 | BP cAMP-mediated signaling |  |
|  | GO:0019935 | 0.0007086 | Up | 1 | 11 | BP cyclic-nucleotide-mediated signaling |  |
|  | GO:0031369 | 0.0007356 | Up | 1 | 10 | MF translation initiation factor binding |  |
|  | GO:0045947 | 0.0007905 | Up | 1 | 11 | BP negative regulation of translational initiation |  |
|  | GO:0008286 | 0.0015697 | Up | 1 | 25 | BP insulin receptor signaling pathway |  |
|  | GO:0019932 | 0.0020246 | Up | 1 | 32 | BP second-messenger-mediated signaling |  |
|  | GO:0017148 | 0.0020802 | Up | 1 | 31 | BP negative regulation of translation |  |
|  | GO:0006446 | 0.0031283 | Up | 1 | 50 | BP regulation of translational initiation |  |
|  | GO:0007169 | 0.0079486 | Up | 1 | 131 | BP transmembrane receptor protein tyrosine kinase signaling pathway | |
|  | GO:0006417 | 0.0099888 | Up | 1 | 159 | BP regulation of translation |  |
|  | GO:0007167 | 0.0102802 | Up | 1 | 172 | BP enzyme linked receptor protein signaling pathway |  |
|  | GO:0032269 | 0.0116042 | Up | 1 | 193 | BP negative regulation of cellular protein metabolic process |  |
|  | GO:0051248 | 0.0128476 | Up | 1 | 214 | BP negative regulation of protein metabolic process |  |
|  | GO:0010608 | 0.014901 | Up | 1 | 240 | BP posttranscriptional regulation of gene expression |  |
|  | GO:0006412 | 0.0188291 | Up | 1 | 341 | BP translation |  |
|  | GO:2000113 | 0.0209461 | Up | 1 | 355 | BP negative regulation of cellular macromolecule biosynthetic process | |
|  | GO:0010558 | 0.0220981 | Up | 1 | 374 | BP negative regulation of macromolecule biosynthetic process |  |
|  | GO:0031327 | 0.0225664 | Up | 1 | 380 | BP negative regulation of cellular biosynthetic process |  |
|  | GO:0009890 | 0.0227827 | Up | 1 | 384 | BP negative regulation of biosynthetic process |  |
|  | GO:0035556 | 0.0257722 | Up | 1 | 441 | BP intracellular signal transduction |  |
|  | GO:0010629 | 0.0258129 | Up | 1 | 438 | BP negative regulation of gene expression |  |
|  | GO:0007166 | 0.0331529 | Up | 1 | 562 | BP cell surface receptor signaling pathway |  |
|  | GO:0032268 | 0.0353371 | Up | 1 | 591 | BP regulation of cellular protein metabolic process |  |
|  | GO:0031324 | 0.0362598 | Up | 1 | 619 | BP negative regulation of cellular metabolic process |  |
|  | GO:0010605 | 0.0373986 | Up | 1 | 639 | BP negative regulation of macromolecule metabolic process |  |
|  | GO:0051246 | 0.039786 | Up | 1 | 668 | BP regulation of protein metabolic process |  |
|  | GO:0009892 | 0.0427687 | Up | 1 | 731 | BP negative regulation of metabolic process |  |
| Control vs. Cold | |  |  |  |  |  |  |
|  | GO:0004553 | 4.48E-05 | Down | 2 | 89 | MF hydrolase activity, hydrolyzing O-glycosyl compounds |  |
|  | GO:0016798 | 5.79E-05 | Down | 2 | 101 | MF hydrolase activity, acting on glycosyl bonds |  |
|  | GO:0008456 | 0.0002498 | Down | 1 | 2 | MF alpha-N-acetylgalactosaminidase activity |  |
|  | GO:0016998 | 0.0005814 | Down | 1 | 5 | BP cell wall macromolecule catabolic process |  |
|  | GO:0044036 | 0.0006922 | Down | 1 | 6 | BP cell wall macromolecule metabolic process |  |
|  | GO:0015929 | 0.0010801 | Down | 1 | 9 | MF hexosaminidase activity |  |
|  | GO:0003796 | 0.0011796 | Down | 1 | 10 | MF lysozyme activity |  |
|  | GO:0019835 | 0.0014055 | Down | 1 | 12 | BP cytolysis |  |
|  | GO:0042742 | 0.0073189 | Down | 1 | 62 | BP defense response to bacterium |  |
|  | GO:0009617 | 0.0078953 | Down | 1 | 67 | BP response to bacterium |  |
|  | GO:0005764 | 0.0117228 | Down | 1 | 99 | CC lysosome |  |
|  | GO:0098542 | 0.0117482 | Down | 1 | 100 | BP defense response to other organism |  |
|  | GO:0000323 | 0.0118467 | Down | 1 | 100 | CC lytic vacuole |  |
|  | GO:0016787 | 0.01428 | Down | 2 | 1663 | MF hydrolase activity |  |
|  | GO:0005773 | 0.0142937 | Down | 1 | 121 | CC vacuole |  |
|  | GO:0051707 | 0.0162917 | Down | 1 | 139 | BP response to other organism |  |
|  | GO:0043207 | 0.0207525 | Down | 1 | 177 | BP response to external biotic stimulus |  |
|  | GO:0009607 | 0.0212364 | Down | 1 | 181 | BP response to biotic stimulus |  |
|  | GO:0006952 | 0.0251179 | Down | 1 | 214 | BP defense response |  |
|  | GO:0009605 | 0.0395195 | Down | 1 | 339 | BP response to external stimulus |  |
|  | GO:0051704 | 0.0403703 | Down | 1 | 347 | BP multi-organism process |  |
|  | GO:0005975 | 0.0455638 | Down | 1 | 389 | BP carbohydrate metabolic process |  |
|  |  |  |  |  |  |  |  |
| *Grylloblatta bifratrilecta* | |  |  |  |  |  |  |
| Control vs Heat | |  |  |  |  |  |  |
|  | GO:0004308 | 0.0030253 | Up | 2 | 2 | MF exo-alpha-sialidase activity |  |
|  | GO:0009405 | 0.0030253 | Up | 2 | 2 | BP pathogenesis |  |
|  | GO:0016997 | 0.0030253 | Up | 2 | 2 | MF alpha-sialidase activity |  |
|  | GO:0052794 | 0.0030253 | Up | 2 | 2 | MF exo-alpha-(2->3)-sialidase activity |  |
|  | GO:0052795 | 0.0030253 | Up | 2 | 2 | MF exo-alpha-(2->6)-sialidase activity |  |
|  | GO:0052796 | 0.0030253 | Up | 2 | 2 | MF exo-alpha-(2->8)-sialidase activity |  |
|  | GO:0031225 | 0.0040569 | Up | 2 | 3 | CC anchored component of membrane |  |
|  | GO:0004553 | 0.0049742 | Up | 2 | 5 | MF hydrolase activity, hydrolyzing O-glycosyl compounds |  |
|  | GO:0016798 | 0.0052563 | Up | 2 | 6 | MF hydrolase activity, acting on glycosyl bonds |  |
|  | GO:0005886 | 0.0082113 | Up | 2 | 18 | CC plasma membrane |  |
|  | GO:0031224 | 0.0107358 | Up | 2 | 23 | CC intrinsic component of membrane |  |
|  | GO:0044425 | 0.0162272 | Up | 2 | 37 | CC membrane part |  |
|  | GO:0016787 | 0.0173337 | Up | 2 | 34 | MF hydrolase activity |  |
|  | GO:0051704 | 0.0175519 | Up | 2 | 27 | BP multi-organism process |  |
|  | GO:0016020 | 0.0203889 | Up | 2 | 50 | CC membrane |  |
| Control vs. Cold | |  |  |  |  |  |  |
|  | Nothing |  |  |  |  |  |  |
|  |  |  |  |  |  |  |  |
| *Grylloblatta* sp. “Lillburn Cave” | | |  |  |  |  |  |
| Control vs Heat | |  |  |  |  |  |  |
|  | Nothing |  |  |  |  |  |  |
| Control vs. Cold | |  |  |  |  |  |  |
|  | GO:0004222 | 0.0039286 | Up | 1 | 56 | MF metalloendopeptidase activity |  |
|  | GO:0005578 | 0.0045338 | Up | 1 | 68 | CC proteinaceous extracellular matrix |  |
|  | GO:0031012 | 0.006055 | Up | 1 | 90 | CC extracellular matrix |  |
|  | GO:0006278 | 0.0084255 | Up | 1 | 130 | BP RNA-dependent DNA biosynthetic process |  |
|  | GO:0006313 | 0.0086903 | Up | 1 | 135 | BP transposition, DNA-mediated |  |
|  | GO:0008237 | 0.0093392 | Up | 1 | 133 | MF metallopeptidase activity |  |
|  | GO:0032196 | 0.0094113 | Up | 1 | 146 | BP transposition |  |
|  | GO:0006260 | 0.0162375 | Up | 1 | 254 | BP DNA replication |  |
|  | GO:0003964 | 0.0174039 | Up | 1 | 270 | MF RNA-directed DNA polymerase activity |  |
|  | GO:0034061 | 0.0193303 | Up | 1 | 301 | MF DNA polymerase activity |  |
|  | GO:0006310 | 0.0199227 | Up | 1 | 310 | BP DNA recombination |  |
|  | GO:0004175 | 0.0241354 | Up | 1 | 367 | MF endopeptidase activity |  |
|  | GO:0016779 | 0.0258115 | Up | 1 | 400 | MF nucleotidyltransferase activity |  |
|  | GO:0043170 | 0.0271075 | Up | 2 | 4453 | BP macromolecule metabolic process |  |
|  | GO:0003824 | 0.0327359 | Up | 2 | 4847 | MF catalytic activity |  |
|  | GO:0006508 | 0.0337021 | Up | 1 | 501 | **BP proteolysis** |  |
|  | GO:0070011 | 0.0362105 | Up | 1 | 546 | MF peptidase activity, acting on L-amino acid peptides |  |
|  | GO:0008233 | 0.0374866 | Up | 1 | 566 | MF peptidase activity |  |
|  | GO:0044238 | 0.0412954 | Up | 2 | 5496 | BP primary metabolic process |  |
|  | GO:0071704 | 0.0452191 | Up | 2 | 5761 | BP organic substance metabolic process |  |
|  |  |  |  |  |  |  |  |
| *Grylloblatta* sp. ‘North Cascades’ at Whitechuck Mountain. | | | | |  |  |  |
| Control vs Heat | |  |  |  |  |  |  |
|  | GO:0003968 | 0.0020358 | Up | 1 | 16 | MF RNA-directed RNA polymerase activity |  |
|  | GO:0004482 | 0.0003615 | Up | 1 | 2 | MF mRNA (guanine-N7-)-methyltransferase activity |  |
|  | GO:0006370 | 0.0015033 | Up | 1 | 10 | BP 7-methylguanosine mRNA capping |  |
|  | GO:0006396 | 0.0349091 | Up | 1 | 304 | BP RNA processing |  |
|  | GO:0006397 | 0.0293544 | Up | 1 | 255 | BP mRNA processing |  |
|  | GO:0008168 | 0.0159931 | Up | 1 | 148 | MF methyltransferase activity |  |
|  | GO:0008170 | 0.0043721 | Up | 1 | 38 | MF N-methyltransferase activity |  |
|  | GO:0008173 | 0.0036615 | Up | 1 | 30 | MF RNA methyltransferase activity |  |
|  | GO:0008174 | 0.0006541 | Up | 1 | 4 | MF mRNA methyltransferase activity |  |
|  | GO:0008757 | 0.0070105 | Up | 1 | 63 | MF S-adenosylmethionine-dependent methyltransferase activity |  |
|  | GO:0009452 | 0.0017389 | Up | 1 | 12 | BP 7-methylguanosine RNA capping |  |
|  | GO:0016071 | 0.0344681 | Up | 1 | 297 | BP mRNA metabolic process |  |
|  | GO:0016741 | 0.0166072 | Up | 1 | 153 | MF transferase activity, transferring one-carbon groups |  |
|  | GO:0019012 | 0.0006596 | Up | 1 | 6 | CC virion |  |
|  | GO:0030430 | 0.0009849 | Up | 1 | 8 | CC host cell cytoplasm |  |
|  | GO:0032259 | 0.0092693 | Up | 1 | 81 | BP methylation |  |
|  | GO:0033643 | 0.0015431 | Up | 1 | 12 | CC host cell part |  |
|  | GO:0033646 | 0.0012152 | Up | 1 | 10 | CC host intracellular part |  |
|  | GO:0034062 | 0.0061949 | Up | 1 | 54 | MF RNA polymerase activity |  |
|  | GO:0036260 | 0.0017389 | Up | 1 | 12 | BP RNA capping |  |
|  | GO:0044217 | 0.0015431 | Up | 1 | 12 | CC other organism part |  |
|  | GO:0001525 | 0.0325072 | Down | 1 | 55 | BP angiogenesis |  |
|  | GO:0001568 | 0.0073375 | Down | 1 | 11 | BP blood vessel development |  |
|  | GO:0001570 | 0.0072417 | Down | 1 | 11 | BP vasculogenesis |  |
|  | GO:0001775 | 0.0334561 | Down | 1 | 54 | BP cell activation |  |
|  | GO:0001936 | 0.0065206 | Down | 1 | 12 | BP regulation of endothelial cell proliferation |  |
|  | GO:0001938 | 0.0053199 | Down | 1 | 10 | BP positive regulation of endothelial cell proliferation |  |
|  | GO:0004252 | 0.0457112 | Down | 1 | 91 | MF serine-type endopeptidase activity |  |
|  | GO:0004768 | 0.0058399 | Down | 1 | 10 | MF stearoyl-CoA 9-desaturase activity |  |
|  | GO:0005488 | 0.026705 | Down | 7 | 5658 | MF binding |  |
|  | GO:0005509 | 0.0002238 | Down | 3 | 204 | MF calcium ion binding |  |
|  | GO:0005515 | 0.0173461 | Down | 4 | 1712 | MF protein binding |  |
|  | GO:0005576 | 0.0208828 | Down | 2 | 410 | CC extracellular region |  |
|  | GO:0005578 | 0.0261378 | Down | 1 | 42 | CC proteinaceous extracellular matrix |  |
|  | GO:0005924 | 0.0382927 | Down | 1 | 64 | CC cell-substrate adherens junction |  |
|  | GO:0005925 | 0.0358429 | Down | 1 | 60 | CC focal adhesion |  |
|  | GO:0006633 | 0.0366336 | Down | 1 | 64 | BP fatty acid biosynthetic process |  |
|  | GO:0007015 | 0.0453595 | Down | 1 | 76 | BP actin filament organization |  |
|  | GO:0007155 | 0.0081975 | Down | 2 | 216 | BP cell adhesion |  |
|  | GO:0007156 | 0.0116147 | Down | 1 | 22 | BP homophilic cell adhesion via plasma membrane adhesion molecules | |
|  | GO:0007163 | 0.0477536 | Down | 1 | 77 | BP establishment or maintenance of cell polarity |  |
|  | GO:0007306 | 0.004058 | Down | 1 | 6 | BP eggshell chorion assembly |  |
|  | GO:0007309 | 0.0222651 | Down | 1 | 35 | BP oocyte axis specification |  |
|  | GO:0007310 | 0.0138772 | Down | 1 | 25 | BP oocyte dorsal/ventral axis specification |  |
|  | GO:0007313 | 0.0009863 | Down | 1 | 1 | BP maternal specification of dorsal/ventral axis, oocyte, soma encoded | |
|  | GO:0007343 | 0.0013651 | Down | 1 | 2 | BP egg activation |  |
|  | GO:0008063 | 0.0133341 | Down | 1 | 21 | BP Toll signaling pathway |  |
|  | GO:0008284 | 0.0481589 | Down | 1 | 91 | BP positive regulation of cell proliferation |  |
|  | GO:0008593 | 0.0241425 | Down | 1 | 45 | BP regulation of Notch signaling pathway |  |
|  | GO:0009798 | 0.0385277 | Down | 1 | 60 | BP axis specification |  |
|  | GO:0009950 | 0.02219 | Down | 1 | 35 | BP dorsal/ventral axis specification |  |
|  | GO:0010927 | 0.04877 | Down | 1 | 81 | BP cellular component assembly involved in morphogenesis |  |
|  | GO:0016215 | 0.0058399 | Down | 1 | 10 | MF acyl-CoA desaturase activity |  |
|  | GO:0016337 | 0.0274121 | Down | 1 | 51 | BP single organismal cell-cell adhesion |  |
|  | GO:0016485 | 0.0360136 | Down | 1 | 66 | BP protein processing |  |
|  | GO:0016717 | 0.006825 | Down | 1 | 13 | MF oxidoreductase activity, acting on paired donors, with oxidation of a pair of donors resulting in the reduction of molecular oxygen to two molecules of water | |
|  | GO:0022610 | 0.0085942 | Down | 2 | 220 | BP biological adhesion |  |
|  | GO:0030027 | 0.0225112 | Down | 1 | 40 | CC lamellipodium |  |
|  | GO:0030055 | 0.0382927 | Down | 1 | 64 | CC cell-substrate junction |  |
|  | GO:0030175 | 0.0069913 | Down | 1 | 12 | CC filopodium |  |
|  | GO:0030334 | 0.0494425 | Down | 1 | 92 | BP regulation of cell migration |  |
|  | GO:0030336 | 0.0218587 | Down | 1 | 40 | BP negative regulation of cell migration |  |
|  | GO:0031012 | 0.000563 | Down | 2 | 55 | CC extracellular matrix |  |
|  | GO:0032502 | 0.0143942 | Down | 4 | 1669 | BP developmental process |  |
|  | GO:0040013 | 0.0240503 | Down | 1 | 44 | BP negative regulation of locomotion |  |
|  | GO:0043167 | 0.0309783 | Down | 5 | 3306 | MF ion binding |  |
|  | GO:0043169 | 0.0341412 | Down | 4 | 2372 | MF cation binding |  |
|  | GO:0044421 | 0.0008391 | Down | 4 | 750 | CC extracellular region part |  |
|  | GO:0045746 | 0.0071383 | Down | 1 | 13 | BP negative regulation of Notch signaling pathway |  |
|  | GO:0046872 | 0.0333455 | Down | 4 | 2358 | MF metal ion binding |  |
|  | GO:0048646 | 0.0124564 | Down | 2 | 284 | BP anatomical structure formation involved in morphogenesis |  |
|  | GO:0050678 | 0.0187648 | Down | 1 | 37 | BP regulation of epithelial cell proliferation |  |
|  | GO:0050679 | 0.0086835 | Down | 1 | 16 | BP positive regulation of epithelial cell proliferation |  |
|  | GO:0051271 | 0.0231577 | Down | 1 | 42 | BP negative regulation of cellular component movement |  |
|  | GO:0072330 | 0.0422625 | Down | 1 | 74 | BP monocarboxylic acid biosynthetic process |  |
|  | GO:0098602 | 0.0274121 | Down | 1 | 51 | BP single organism cell adhesion |  |
|  | GO:0098609 | 0.0208574 | Down | 1 | 35 | BP cell-cell adhesion |  |
|  | GO:0098742 | 0.0208574 | Down | 1 | 35 | BP cell-cell adhesion via plasma-membrane adhesion molecules |  |
|  | GO:2000146 | 0.0218587 | Down | 1 | 40 | BP negative regulation of cell motility |  |
| Control vs. Cold | |  |  |  |  |  |  |
|  | category | over_represented_pvalue | under_represented_pvalue | numDEInCat | numInCat | term |  |
|  | GO:0000038 | 0.0033682 | Up | 1 | 11 | BP very long-chain fatty acid metabolic process |  |
|  | GO:0000302 | 0.0133918 | Up | 1 | 45 | **BP response to reactive oxygen species** |  |
|  | GO:0000323 | 0.0322307 | Up | 1 | 104 | CC lytic vacuole |  |
|  | GO:0001817 | 0.0242006 | Up | 1 | 82 | BP regulation of cytokine production |  |
|  | GO:0001818 | 0.0096712 | Up | 1 | 32 | BP negative regulation of cytokine production |  |
|  | GO:0001952 | 0.0098826 | Up | 1 | 33 | BP regulation of cell-matrix adhesion |  |
|  | GO:0001953 | 0.0053111 | Up | 1 | 18 | BP negative regulation of cell-matrix adhesion |  |
|  | GO:0002683 | 0.0236767 | Up | 1 | 80 | BP negative regulation of immune system process |  |
|  | GO:0002685 | 0.0050547 | Up | 1 | 17 | BP regulation of leukocyte migration |  |
|  | GO:0002686 | 0.0039212 | Up | 1 | 13 | BP negative regulation of leukocyte migration |  |
|  | GO:0003735 | 0.0425052 | Up | 1 | 147 | MF structural constituent of ribosome |  |
|  | GO:0003906 | 0.0022728 | Up | 1 | 7 | MF DNA-(apurinic or apyrimidinic site) lyase activity |  |
|  | GO:0004197 | 0.0123869 | Up | 1 | 41 | MF cysteine-type endopeptidase activity |  |
|  | GO:0004520 | 0.0101279 | Up | 1 | 34 | MF endodeoxyribonuclease activity |  |
|  | GO:0004536 | 0.0125694 | Up | 1 | 42 | MF deoxyribonuclease activity |  |
|  | GO:0005496 | 0.009702 | Up | 1 | 31 | MF steroid binding |  |
|  | GO:0005764 | 0.0322307 | Up | 1 | 104 | CC lysosome |  |
|  | GO:0005773 | 0.0373231 | Up | 1 | 120 | CC vacuole |  |
|  | GO:0005783 | 0.004191 | Up | 2 | 313 | CC endoplasmic reticulum |  |
|  | GO:0005819 | 0.0233111 | Up | 1 | 79 | CC spindle |  |
|  | GO:0005840 | 0.0010264 | Up | 2 | 159 | CC ribosome |  |
|  | GO:0005996 | 0.0287943 | Up | 1 | 96 | BP monosaccharide metabolic process |  |
|  | GO:0006006 | 0.0154714 | Up | 1 | 50 | BP glucose metabolic process |  |
|  | GO:0006259 | 0.0355137 | Up | 2 | 996 | BP DNA metabolic process |  |
|  | GO:0006412 | 0.0470979 | Up | 1 | 161 | BP translation |  |
|  | GO:0006417 | 0.0431545 | Up | 1 | 139 | BP regulation of translation |  |
|  | GO:0006629 | 0.0104175 | Up | 2 | 507 | BP lipid metabolic process |  |
|  | GO:0006631 | 0.0409038 | Up | 1 | 134 | BP fatty acid metabolic process |  |
|  | GO:0006633 | 0.0206415 | Up | 1 | 67 | BP fatty acid biosynthetic process |  |
|  | GO:0006950 | 0.0342283 | Up | 2 | 961 | **BP response to stress** | TR14492\|c3_g1: rps3 40S Ribosomal protein; TR9159\|c0_g1: Apolipoprotein D |
|  | GO:0006979 | 0.0311635 | Up | 1 | 107 | **BP response to oxidative stress** |  |
|  | GO:0007067 | 0.0456614 | Up | 1 | 152 | BP mitotic nuclear division |  |
|  | GO:0007162 | 0.0157147 | Up | 1 | 52 | BP negative regulation of cell adhesion |  |
|  | GO:0007420 | 0.0210635 | Up | 1 | 70 | BP brain development |  |
|  | GO:0007568 | 0.0202546 | Up | 1 | 65 | BP aging |  |
|  | GO:0008234 | 0.0264991 | Up | 1 | 86 | MF cysteine-type peptidase activity |  |
|  | GO:0008285 | 0.0483822 | Up | 1 | 162 | BP negative regulation of cell proliferation |  |
|  | GO:0009058 | 0.0378504 | Up | 3 | 2559 | BP biosynthetic process |  |
|  | GO:0009611 | 0.0166697 | Up | 1 | 51 | BP response to wounding |  |
|  | GO:0010640 | 0.0045366 | Up | 1 | 15 | BP regulation of platelet-derived growth factor receptor signaling pathway | |
|  | GO:0010642 | 0.0045366 | Up | 1 | 15 | BP negative regulation of platelet-derived growth factor receptor signaling pathway | |
|  | GO:0010810 | 0.0162817 | Up | 1 | 54 | BP regulation of cell-substrate adhesion |  |
|  | GO:0010812 | 0.0070259 | Up | 1 | 23 | BP negative regulation of cell-substrate adhesion |  |
|  | GO:0014012 | 0.0036208 | Up | 1 | 12 | BP peripheral nervous system axon regeneration |  |
|  | GO:0015485 | 0.0083236 | Up | 1 | 26 | MF cholesterol binding |  |
|  | GO:0015935 | 0.0094853 | Up | 1 | 32 | CC small ribosomal subunit |  |
|  | GO:0016053 | 0.0411443 | Up | 1 | 136 | BP organic acid biosynthetic process |  |
|  | GO:0016829 | 0.0442815 | Up | 1 | 146 | MF lyase activity |  |
|  | GO:0016835 | 0.0138526 | Up | 1 | 44 | MF carbon-oxygen lyase activity |  |
|  | GO:0019216 | 0.0245851 | Up | 1 | 81 | BP regulation of lipid metabolic process |  |
|  | GO:0019318 | 0.0241301 | Up | 1 | 80 | BP hexose metabolic process |  |
|  | GO:0019367 | 0.0015304 | Up | 1 | 5 | BP fatty acid elongation, saturated fatty acid |  |
|  | GO:0022626 | 0.0041688 | Up | 1 | 14 | CC cytosolic ribosome |  |
|  | GO:0030155 | 0.0373561 | Up | 1 | 124 | BP regulation of cell adhesion |  |
|  | GO:0030176 | 0.0123856 | Up | 1 | 41 | CC integral component of endoplasmic reticulum membrane |  |
|  | GO:0030334 | 0.0376617 | Up | 1 | 128 | BP regulation of cell migration |  |
|  | GO:0030336 | 0.0161032 | Up | 1 | 54 | BP negative regulation of cell migration |  |
|  | GO:0030425 | 0.0284249 | Up | 1 | 95 | CC dendrite |  |
|  | GO:0030497 | 0.002438 | Up | 1 | 8 | BP fatty acid elongation |  |
|  | GO:0030529 | 0.0102746 | Up | 2 | 511 | CC intracellular ribonucleoprotein complex |  |
|  | GO:0031099 | 0.0107277 | Up | 1 | 35 | BP regeneration |  |
|  | GO:0031102 | 0.0041697 | Up | 1 | 14 | BP neuron projection regeneration |  |
|  | GO:0031103 | 0.0036208 | Up | 1 | 12 | BP axon regeneration |  |
|  | GO:0031175 | 0.0136214 | Up | 1 | 44 | BP neuron projection development |  |
|  | GO:0031227 | 0.0147835 | Up | 1 | 47 | CC intrinsic component of endoplasmic reticulum membrane |  |
|  | GO:0031300 | 0.0272898 | Up | 1 | 90 | CC intrinsic component of organelle membrane |  |
|  | GO:0031301 | 0.024072 | Up | 1 | 81 | CC integral component of organelle membrane |  |
|  | GO:0031347 | 0.0375307 | Up | 1 | 125 | BP regulation of defense response |  |
|  | GO:0031348 | 0.0118946 | Up | 1 | 39 | BP negative regulation of defense response |  |
|  | GO:0032101 | 0.0421954 | Up | 1 | 145 | BP regulation of response to external stimulus |  |
|  | GO:0032102 | 0.0167991 | Up | 1 | 57 | BP negative regulation of response to external stimulus |  |
|  | GO:0032386 | 0.0416573 | Up | 1 | 139 | BP regulation of intracellular transport |  |
|  | GO:0032387 | 0.0126172 | Up | 1 | 41 | BP negative regulation of intracellular transport |  |
|  | GO:0032642 | 0.0047407 | Up | 1 | 16 | BP regulation of chemokine production |  |
|  | GO:0032682 | 0.0033259 | Up | 1 | 11 | BP negative regulation of chemokine production |  |
|  | GO:0032934 | 0.0083236 | Up | 1 | 26 | MF sterol binding |  |
|  | GO:0033157 | 0.0316098 | Up | 1 | 105 | BP regulation of intracellular protein transport |  |
|  | GO:0033554 | 0.0119216 | Up | 2 | 547 | **BP cellular response to stress** | TR14492\|c3_g1: rps3 40S Ribosomal protein; TR9159\|c0_g1: Apolipoprotein D |
|  | GO:0034442 | 0.0033259 | Up | 1 | 11 | BP regulation of lipoprotein oxidation |  |
|  | GO:0034443 | 0.0033259 | Up | 1 | 11 | BP negative regulation of lipoprotein oxidation |  |
|  | GO:0040012 | 0.0478706 | Up | 1 | 163 | BP regulation of locomotion |  |
|  | GO:0040013 | 0.017595 | Up | 1 | 59 | BP negative regulation of locomotion |  |
|  | GO:0042246 | 0.0053676 | Up | 1 | 18 | BP tissue regeneration |  |
|  | GO:0042306 | 0.0189066 | Up | 1 | 64 | BP regulation of protein import into nucleus |  |
|  | GO:0042308 | 0.0074237 | Up | 1 | 24 | BP negative regulation of protein import into nucleus |  |
|  | GO:0042493 | 0.0300184 | Up | 1 | 96 | BP response to drug |  |
|  | GO:0042761 | 0.0014832 | Up | 1 | 5 | BP very long-chain fatty acid biosynthetic process |  |
|  | GO:0043025 | 0.0279513 | Up | 1 | 93 | CC neuronal cell body |  |
|  | GO:0043178 | 0.0098541 | Up | 1 | 31 | MF alcohol binding |  |
|  | GO:0044249 | 0.0336405 | Up | 3 | 2449 | BP cellular biosynthetic process |  |
|  | GO:0044297 | 0.0314465 | Up | 1 | 104 | CC cell body |  |
|  | GO:0044391 | 0.019763 | Up | 1 | 67 | CC ribosomal subunit |  |
|  | GO:0044445 | 0.0200478 | Up | 1 | 67 | CC cytosolic part |  |
|  | GO:0044710 | 0.0070859 | Up | 4 | 2642 | BP single-organism metabolic process |  |
|  | GO:0044763 | 0.046339 | Up | 4 | 4633 | BP single-organism cellular process |  |
|  | GO:0045833 | 0.0057644 | Up | 1 | 19 | BP negative regulation of lipid metabolic process |  |
|  | GO:0046394 | 0.0411443 | Up | 1 | 136 | BP carboxylic acid biosynthetic process |  |
|  | GO:0046822 | 0.0249748 | Up | 1 | 84 | BP regulation of nucleocytoplasmic transport |  |
|  | GO:0046823 | 0.0088981 | Up | 1 | 29 | BP negative regulation of nucleocytoplasmic transport |  |
|  | GO:0048589 | 0.0382247 | Up | 1 | 125 | BP developmental growth |  |
|  | GO:0048660 | 0.0067573 | Up | 1 | 23 | BP regulation of smooth muscle cell proliferation |  |
|  | GO:0048662 | 0.0040883 | Up | 1 | 14 | BP negative regulation of smooth muscle cell proliferation |  |
|  | GO:0048678 | 0.0055906 | Up | 1 | 18 | BP response to axon injury |  |
|  | GO:0050727 | 0.013958 | Up | 1 | 47 | **BP regulation of inflammatory response** |  |
|  | GO:0050728 | 0.0075453 | Up | 1 | 25 | BP negative regulation of inflammatory response |  |
|  | GO:0050746 | 0.0033259 | Up | 1 | 11 | BP regulation of lipoprotein metabolic process |  |
|  | GO:0050748 | 0.0033259 | Up | 1 | 11 | BP negative regulation of lipoprotein metabolic process |  |
|  | GO:0051051 | 0.0261232 | Up | 1 | 87 | BP negative regulation of transport |  |
|  | GO:0051223 | 0.0478966 | Up | 1 | 159 | BP regulation of protein transport |  |
|  | GO:0051224 | 0.0149937 | Up | 1 | 49 | BP negative regulation of protein transport |  |
|  | GO:0051246 | 0.0153815 | Up | 2 | 619 | BP regulation of protein metabolic process |  |
|  | GO:0051270 | 0.0464128 | Up | 1 | 158 | BP regulation of cellular component movement |  |
|  | GO:0051271 | 0.0169971 | Up | 1 | 57 | BP negative regulation of cellular component movement |  |
|  | GO:0051716 | 0.0203621 | Up | 2 | 730 | BP cellular response to stimulus |  |
|  | GO:0051893 | 0.0068536 | Up | 1 | 22 | BP regulation of focal adhesion assembly |  |
|  | GO:0051895 | 0.0037187 | Up | 1 | 12 | BP negative regulation of focal adhesion assembly |  |
|  | GO:0060587 | 0.0033259 | Up | 1 | 11 | BP regulation of lipoprotein lipid oxidation |  |
|  | GO:0060588 | 0.0033259 | Up | 1 | 11 | BP negative regulation of lipoprotein lipid oxidation |  |
|  | GO:0061564 | 0.0041971 | Up | 1 | 14 | BP axon development |  |
|  | GO:0071637 | 0.0033259 | Up | 1 | 11 | BP regulation of monocyte chemotactic protein-1 production |  |
|  | GO:0071638 | 0.0033259 | Up | 1 | 11 | BP negative regulation of monocyte chemotactic protein-1 production | |
|  | GO:0072330 | 0.0236633 | Up | 1 | 77 | BP monocarboxylic acid biosynthetic process |  |
|  | GO:0090109 | 0.0068536 | Up | 1 | 22 | BP regulation of cell-substrate junction assembly |  |
|  | GO:0090317 | 0.0099037 | Up | 1 | 32 | BP negative regulation of intracellular protein transport |  |
|  | GO:1900015 | 0.0033259 | Up | 1 | 11 | BP regulation of cytokine production involved in inflammatory response | |
|  | GO:1900016 | 0.0033259 | Up | 1 | 11 | BP negative regulation of cytokine production involved in inflammatory response | |
|  | GO:1900180 | 0.0217284 | Up | 1 | 74 | BP regulation of protein localization to nucleus |  |
|  | GO:1900181 | 0.0097155 | Up | 1 | 32 | BP negative regulation of protein localization to nucleus |  |
|  | GO:1901576 | 0.0357919 | Up | 3 | 2504 | BP organic substance biosynthetic process |  |
|  | GO:1901888 | 0.0070986 | Up | 1 | 23 | BP regulation of cell junction assembly |  |
|  | GO:1901889 | 0.0037187 | Up | 1 | 12 | BP negative regulation of cell junction assembly |  |
|  | GO:1903034 | 0.0223367 | Up | 1 | 74 | BP regulation of response to wounding |  |
|  | GO:1903035 | 0.0104793 | Up | 1 | 34 | BP negative regulation of response to wounding |  |
|  | GO:1903391 | 0.0068536 | Up | 1 | 22 | BP regulation of adherens junction organization |  |
|  | GO:1903392 | 0.0037187 | Up | 1 | 12 | BP negative regulation of adherens junction organization |  |
|  | GO:1903533 | 0.0225327 | Up | 1 | 75 | BP regulation of protein targeting |  |
|  | GO:1903649 | 0.0312407 | Up | 1 | 105 | BP regulation of cytoplasmic transport |  |
|  | GO:1903650 | 0.0101887 | Up | 1 | 33 | BP negative regulation of cytoplasmic transport |  |
|  | GO:1903827 | 0.0417668 | Up | 1 | 139 | BP regulation of cellular protein localization |  |
|  | GO:1903828 | 0.0160657 | Up | 1 | 52 | BP negative regulation of cellular protein localization |  |
|  | GO:2000097 | 0.0033259 | Up | 1 | 11 | BP regulation of smooth muscle cell-matrix adhesion |  |
|  | GO:2000098 | 0.0033259 | Up | 1 | 11 | BP negative regulation of smooth muscle cell-matrix adhesion |  |
|  | GO:2000145 | 0.0399003 | Up | 1 | 136 | BP regulation of cell motility |  |
|  | GO:2000146 | 0.0161032 | Up | 1 | 54 | BP negative regulation of cell motility |  |
|  | GO:2000401 | 0.0038254 | Up | 1 | 13 | BP regulation of lymphocyte migration |  |
|  | GO:2000402 | 0.0033259 | Up | 1 | 11 | BP negative regulation of lymphocyte migration |  |
|  | GO:2000404 | 0.0035797 | Up | 1 | 12 | BP regulation of T cell migration |  |
|  | GO:2000405 | 0.0033259 | Up | 1 | 11 | BP negative regulation of T cell migration |  |
|  | GO:0001568 | 0.0154291 | Down | 1 | 14 | BP blood vessel development |  |
|  | GO:0001570 | 0.015572 | Down | 1 | 15 | BP vasculogenesis |  |
|  | GO:0001936 | 0.0133749 | Down | 1 | 12 | BP regulation of endothelial cell proliferation |  |
|  | GO:0001938 | 0.0109567 | Down | 1 | 10 | BP positive regulation of endothelial cell proliferation |  |
|  | GO:0003674 | 0.0020036 | Down | 19 | 9174 | MF molecular_function |  |
|  | GO:0003824 | 0.0079764 | Down | 11 | 4517 | MF catalytic activity |  |
|  | GO:0003954 | 0.040674 | Down | 1 | 40 | MF NADH dehydrogenase activity |  |
|  | GO:0004129 | 3.56E-08 | Down | 4 | 34 | MF cytochrome-c oxidase activity |  |
|  | GO:0004601 | 0.0412542 | Down | 1 | 41 | MF peroxidase activity |  |
|  | GO:0005215 | 9.69E-05 | Down | 6 | 695 | MF transporter activity |  |
|  | GO:0005506 | 0.0143155 | Down | 2 | 173 | MF iron ion binding |  |
|  | GO:0005507 | 0.0263117 | Down | 1 | 25 | MF copper ion binding |  |
|  | GO:0005509 | 0.0002239 | Down | 4 | 281 | MF calcium ion binding |  |
|  | GO:0005575 | 0.0086498 | Down | 17 | 8829 | CC cellular_component |  |
|  | GO:0005576 | 0.0018028 | Down | 4 | 471 | CC extracellular region |  |
|  | GO:0005615 | 0.030503 | Down | 2 | 249 | CC extracellular space |  |
|  | GO:0005743 | 2.48E-10 | Down | 8 | 242 | CC mitochondrial inner membrane |  |
|  | GO:0006091 | 5.16E-07 | Down | 5 | 143 | BP generation of precursor metabolites and energy |  |
|  | GO:0006119 | 7.00E-05 | Down | 2 | 14 | BP oxidative phosphorylation |  |
|  | GO:0006163 | 0.0040849 | Down | 3 | 301 | BP purine nucleotide metabolic process |  |
|  | GO:0006753 | 0.0096659 | Down | 3 | 407 | BP nucleoside phosphate metabolic process |  |
|  | GO:0006754 | 0.0278719 | Down | 1 | 28 | BP ATP biosynthetic process |  |
|  | GO:0007155 | 0.0384022 | Down | 2 | 289 | BP cell adhesion |  |
|  | GO:0007156 | 0.0310253 | Down | 1 | 31 | BP homophilic cell adhesion via plasma membrane adhesion molecules | |
|  | GO:0007306 | 0.0102516 | Down | 1 | 10 | BP eggshell chorion assembly |  |
|  | GO:0008088 | 0.0333536 | Down | 1 | 30 | BP axo-dendritic transport |  |
|  | GO:0008089 | 0.011486 | Down | 1 | 10 | BP anterograde axonal transport |  |
|  | GO:0008137 | 0.03946 | Down | 1 | 39 | MF NADH dehydrogenase (ubiquinone) activity |  |
|  | GO:0008150 | 0.0338136 | Down | 15 | 8609 | BP biological_process |  |
|  | GO:0008152 | 0.0443475 | Down | 11 | 5854 | **BP metabolic process** |  |
|  | GO:0008289 | 0.025798 | Down | 2 | 227 | MF lipid binding |  |
|  | GO:0008324 | 1.84E-05 | Down | 5 | 308 | MF cation transmembrane transporter activity |  |
|  | GO:0009055 | 1.73E-08 | Down | 5 | 76 | MF electron carrier activity |  |
|  | GO:0009060 | 1.09E-06 | Down | 3 | 21 | BP aerobic respiration |  |
|  | GO:0009116 | 0.0047063 | Down | 3 | 315 | BP nucleoside metabolic process |  |
|  | GO:0009117 | 0.0094027 | Down | 3 | 403 | BP nucleotide metabolic process |  |
|  | GO:0009119 | 0.0040202 | Down | 3 | 297 | BP ribonucleoside metabolic process |  |
|  | GO:0009123 | 0.0014933 | Down | 3 | 216 | BP nucleoside monophosphate metabolic process |  |
|  | GO:0009126 | 0.0011791 | Down | 3 | 199 | BP purine nucleoside monophosphate metabolic process |  |
|  | GO:0009141 | 0.0021392 | Down | 3 | 240 | BP nucleoside triphosphate metabolic process |  |
|  | GO:0009142 | 0.0370462 | Down | 1 | 37 | BP nucleoside triphosphate biosynthetic process |  |
|  | GO:0009144 | 0.0019895 | Down | 3 | 234 | BP purine nucleoside triphosphate metabolic process |  |
|  | GO:0009145 | 0.0319495 | Down | 1 | 32 | BP purine nucleoside triphosphate biosynthetic process |  |
|  | GO:0009150 | 0.0037933 | Down | 3 | 293 | BP purine ribonucleotide metabolic process |  |
|  | GO:0009161 | 0.0013776 | Down | 3 | 210 | BP ribonucleoside monophosphate metabolic process |  |
|  | GO:0009167 | 0.0011791 | Down | 3 | 199 | BP purine ribonucleoside monophosphate metabolic process |  |
|  | GO:0009199 | 0.0019704 | Down | 3 | 233 | BP ribonucleoside triphosphate metabolic process |  |
|  | GO:0009201 | 0.0319495 | Down | 1 | 32 | BP ribonucleoside triphosphate biosynthetic process |  |
|  | GO:0009205 | 0.0019704 | Down | 3 | 233 | BP purine ribonucleoside triphosphate metabolic process |  |
|  | GO:0009206 | 0.0319495 | Down | 1 | 32 | BP purine ribonucleoside triphosphate biosynthetic process |  |
|  | GO:0009259 | 0.0041525 | Down | 3 | 303 | BP ribonucleotide metabolic process |  |
|  | GO:0009968 | 0.0390158 | Down | 2 | 294 | BP negative regulation of signal transduction |  |
|  | GO:0009987 | 0.0110641 | Down | 14 | 6856 | BP cellular process |  |
|  | GO:0010648 | 0.0456224 | Down | 2 | 320 | BP negative regulation of cell communication |  |
|  | GO:0015002 | 3.56E-08 | Down | 4 | 34 | MF heme-copper terminal oxidase activity |  |
|  | GO:0015075 | 7.27E-05 | Down | 5 | 412 | MF ion transmembrane transporter activity |  |
|  | GO:0015077 | 6.22E-07 | Down | 5 | 155 | MF monovalent inorganic cation transmembrane transporter activity | |
|  | GO:0015078 | 3.54E-08 | Down | 5 | 87 | MF hydrogen ion transmembrane transporter activity |  |
|  | GO:0015980 | 3.97E-05 | Down | 3 | 61 | BP energy derivation by oxidation of organic compounds |  |
|  | GO:0015985 | 0.0192529 | Down | 1 | 19 | BP energy coupled proton transport, down electrochemical gradient |  |
|  | GO:0015986 | 0.0192529 | Down | 1 | 19 | BP ATP synthesis coupled proton transport |  |
|  | GO:0016020 | 0.0019328 | Down | 9 | 2641 | CC membrane |  |
|  | GO:0016021 | 3.23E-05 | Down | 10 | 1922 | CC integral component of membrane |  |
|  | GO:0016310 | 0.0472478 | Down | 2 | 323 | BP phosphorylation |  |
|  | GO:0016491 | 7.25E-06 | Down | 7 | 660 | **MF oxidoreductase activity** |  |
|  | GO:0016655 | 0.0417889 | Down | 1 | 41 | MF oxidoreductase activity, acting on NAD(P)H, quinone or similar compound as acceptor | |
|  | GO:0016675 | 3.56E-08 | Down | 4 | 34 | MF oxidoreductase activity, acting on a heme group of donors |  |
|  | GO:0016676 | 3.56E-08 | Down | 4 | 34 | MF oxidoreductase activity, acting on a heme group of donors, oxygen as acceptor | |
|  | GO:0016684 | 0.0412542 | Down | 1 | 41 | MF oxidoreductase activity, acting on peroxide as acceptor |  |
|  | GO:0019637 | 0.025774 | Down | 3 | 590 | BP organophosphate metabolic process |  |
|  | GO:0019646 | 0.0037626 | Down | 1 | 4 | BP aerobic electron transport chain |  |
|  | GO:0019693 | 0.0049324 | Down | 3 | 322 | BP ribose phosphate metabolic process |  |
|  | GO:0019866 | 3.68E-10 | Down | 8 | 254 | CC organelle inner membrane |  |
|  | GO:0020037 | 0.0006072 | Down | 3 | 162 | MF heme binding |  |
|  | GO:0022610 | 0.0397284 | Down | 2 | 294 | BP biological adhesion |  |
|  | GO:0022857 | 0.0002687 | Down | 5 | 541 | MF transmembrane transporter activity |  |
|  | GO:0022890 | 6.52E-06 | Down | 5 | 249 | MF inorganic cation transmembrane transporter activity |  |
|  | GO:0022891 | 0.0001334 | Down | 5 | 466 | MF substrate-specific transmembrane transporter activity |  |
|  | GO:0022892 | 0.000284 | Down | 5 | 546 | MF substrate-specific transporter activity |  |
|  | GO:0022900 | 4.07E-05 | Down | 3 | 63 | BP electron transport chain |  |
|  | GO:0022904 | 0.0014928 | Down | 2 | 54 | BP respiratory electron transport chain |  |
|  | GO:0023057 | 0.0450294 | Down | 2 | 318 | BP negative regulation of signaling |  |
|  | GO:0030175 | 0.0153262 | Down | 1 | 14 | CC filopodium |  |
|  | GO:0030312 | 0.0067172 | Down | 1 | 7 | CC external encapsulating structure |  |
|  | GO:0031082 | 0.0103912 | Down | 1 | 9 | CC BLOC complex |  |
|  | GO:0031083 | 0.0103912 | Down | 1 | 9 | CC BLOC-1 complex |  |
|  | GO:0031090 | 0.0001009 | Down | 8 | 1363 | CC organelle membrane |  |
|  | GO:0031224 | 3.96E-05 | Down | 10 | 1971 | CC intrinsic component of membrane |  |
|  | GO:0031966 | 3.15E-09 | Down | 8 | 333 | CC mitochondrial membrane |  |
|  | GO:0033177 | 0.0256021 | Down | 1 | 24 | CC proton-transporting two-sector ATPase complex, proton-transporting domain | |
|  | GO:0042127 | 0.0450764 | Down | 2 | 317 | BP regulation of cell proliferation |  |
|  | GO:0042278 | 0.0039727 | Down | 3 | 296 | BP purine nucleoside metabolic process |  |
|  | GO:0042302 | 0.049881 | Down | 1 | 49 | MF structural constituent of cuticle |  |
|  | GO:0042600 | 0.004831 | Down | 1 | 5 | CC chorion |  |
|  | GO:0042743 | 0.0328824 | Down | 1 | 33 | BP hydrogen peroxide metabolic process |  |
|  | GO:0042744 | 0.0293955 | Down | 1 | 30 | BP hydrogen peroxide catabolic process |  |
|  | GO:0043167 | 0.0288803 | Down | 9 | 4178 | MF ion binding |  |
|  | GO:0043169 | 0.0035789 | Down | 9 | 3020 | MF cation binding |  |
|  | GO:0044237 | 0.0442019 | Down | 10 | 5146 | BP cellular metabolic process |  |
|  | GO:0044281 | 0.0379019 | Down | 4 | 1148 | BP small molecule metabolic process |  |
|  | GO:0044422 | 0.00471 | Down | 10 | 3570 | CC organelle part |  |
|  | GO:0044425 | 0.0003429 | Down | 10 | 2568 | CC membrane part |  |
|  | GO:0044429 | 2.31E-07 | Down | 8 | 573 | CC mitochondrial part |  |
|  | GO:0044444 | 0.0003499 | Down | 12 | 3537 | CC cytoplasmic part |  |
|  | GO:0044446 | 0.0040663 | Down | 10 | 3496 | CC intracellular organelle part |  |
|  | GO:0044699 | 0.0007612 | Down | 15 | 5719 | BP single-organism process |  |
|  | GO:0045263 | 0.0131507 | Down | 1 | 14 | CC proton-transporting ATP synthase complex, coupling factor F(o) |  |
|  | GO:0045333 | 3.98E-06 | Down | 3 | 30 | **BP cellular respiration** |  |
|  | GO:0045746 | 0.0160857 | Down | 1 | 16 | BP negative regulation of Notch signaling pathway |  |
|  | GO:0046034 | 0.0008019 | Down | 3 | 175 | BP ATP metabolic process |  |
|  | GO:0046128 | 0.0035878 | Down | 3 | 285 | BP purine ribonucleoside metabolic process |  |
|  | GO:0046872 | 0.0034345 | Down | 9 | 3003 | MF metal ion binding |  |
|  | GO:0046906 | 0.0006411 | Down | 3 | 165 | MF tetrapyrrole binding |  |
|  | GO:0048471 | 0.0211417 | Down | 2 | 206 | CC perinuclear region of cytoplasm |  |
|  | GO:0048490 | 0.0102297 | Down | 1 | 9 | BP anterograde synaptic vesicle transport |  |
|  | GO:0050136 | 0.03946 | Down | 1 | 39 | MF NADH dehydrogenase (quinone) activity |  |
|  | GO:0050678 | 0.0488201 | Down | 1 | 46 | BP regulation of epithelial cell proliferation |  |
|  | GO:0050679 | 0.0185844 | Down | 1 | 17 | BP positive regulation of epithelial cell proliferation |  |
|  | GO:0055086 | 0.0129862 | Down | 3 | 452 | BP nucleobase-containing small molecule metabolic process |  |
|  | GO:0055114 | 0.0002358 | Down | 5 | 492 | **BP oxidation-reduction process** |  |
|  | GO:0070469 | 1.10E-08 | Down | 5 | 73 | CC respiratory chain |  |
|  | GO:0072521 | 0.005914 | Down | 3 | 342 | BP purine-containing compound metabolic process |  |
|  | GO:0098609 | 0.049399 | Down | 1 | 48 | BP cell-cell adhesion |  |
|  | GO:0098742 | 0.049399 | Down | 1 | 48 | BP cell-cell adhesion via plasma-membrane adhesion molecules |  |
|  | GO:1901135 | 0.027869 | Down | 3 | 607 | BP carbohydrate derivative metabolic process |  |
|  | GO:1901657 | 0.0047734 | Down | 3 | 317 | BP glycosyl compound metabolic process |  |
|  | GO:1902600 | 0.049272 | Down | 1 | 46 | BP hydrogen ion transmembrane transport |  |

**Supplementary Table** **S13**. Results of the test for enrichment of gene ontology categories following the acute temperature stress exposure. Samples without biological replicates are shown here, where significance tests are based on a dispersion parameter of 0.1.

| **Species** | **GO Term** | **Enriched *p-value*** | **Diretion in Control Sample** | **# DE genes** | **Total # genes** | **Annotation** | **Genes** |
| --- | --- | --- | --- | --- | --- | --- | --- |
| *Grylloblatta marmoreus* | |  |  |  |  |  |  |
| Control vs Heat | |  |  |  |  |  |  |
|  | GO:0005576 | 3.57E-06 | Up | 8 | 377 | CC extracellular region |  |
|  | GO:0045735 | 0.0011396 | Up | 2 | 10 | MF nutrient reservoir activity |  |
|  | GO:0005319 | 0.008533 | Up | 2 | 45 | MF lipid transporter activity |  |
|  | GO:0006869 | 0.0412242 | Up | 2 | 114 | BP lipid transport |  |
|  | GO:0022892 | 0.0208042 | Up | 2 | 447 | MF substrate-specific transporter activity |  |
|  | GO:0005215 | 0.0334454 | Up | 2 | 576 | MF transporter activity |  |
|  | GO:0004252 | 0 | Down | 8 | 64 | MF serine-type endopeptidase activity |  |
|  | GO:0008236 | 4.78E-12 | Down | 8 | 74 | MF serine-type peptidase activity |  |
|  | GO:0017171 | 7.46E-12 | Down | 8 | 76 | MF serine hydrolase activity |  |
|  | GO:0030574 | 3.54E-09 | Down | 4 | 9 | BP collagen catabolic process |  |
|  | GO:0044243 | 3.54E-09 | Down | 4 | 9 | BP multicellular organism catabolic process |  |
|  | GO:0006508 | 4.73E-09 | Down | 10 | 298 | BP proteolysis |  |
|  | GO:0070011 | 6.95E-09 | Down | 10 | 319 | MF peptidase activity, acting on L-amino acid peptides |  |
|  | GO:0032963 | 7.88E-09 | Down | 4 | 11 | BP collagen metabolic process |  |
|  | GO:0044236 | 7.88E-09 | Down | 4 | 11 | BP multicellular organism metabolic process |  |
|  | GO:0044259 | 7.88E-09 | Down | 4 | 11 | BP multicellular organismal macromolecule metabolic process |  |
|  | GO:0008233 | 9.87E-09 | Down | 10 | 332 | MF peptidase activity |  |
|  | GO:0004175 | 2.87E-08 | Down | 8 | 199 | MF endopeptidase activity |  |
|  | GO:0016787 | 1.60E-07 | Down | 16 | 1337 | MF hydrolase activity |  |
|  | GO:0005615 | 7.23E-07 | Down | 7 | 187 | CC extracellular space |  |
|  | GO:0016798 | 5.72E-06 | Down | 5 | 97 | MF hydrolase activity, acting on glycosyl bonds |  |
|  | GO:0004553 | 6.76E-05 | Down | 4 | 79 | MF hydrolase activity, hydrolyzing O-glycosyl compounds |  |
|  | GO:0007586 | 0.0001418 | Down | 2 | 8 | BP digestion |  |
|  | GO:0009056 | 0.0001672 | Down | 9 | 765 | BP catabolic process |  |
|  | GO:0044712 | 0.0001711 | Down | 7 | 444 | BP single-organism catabolic process |  |
|  | GO:0005975 | 0.0002471 | Down | 6 | 343 | BP carbohydrate metabolic process |  |
|  | GO:0015929 | 0.0002941 | Down | 2 | 8 | MF hexosaminidase activity |  |
|  | GO:0030149 | 0.0003616 | Down | 2 | 10 | BP sphingolipid catabolic process |  |
|  | GO:0044421 | 0.0003935 | Down | 8 | 675 | CC extracellular region part |  |
|  | GO:0046466 | 0.0005089 | Down | 2 | 12 | BP membrane lipid catabolic process |  |
|  | GO:0006032 | 0.0005304 | Down | 2 | 13 | BP chitin catabolic process |  |
|  | GO:0032501 | 0.0005644 | Down | 9 | 940 | BP multicellular organismal process |  |
|  | GO:1901072 | 0.0006131 | Down | 2 | 14 | BP glucosamine-containing compound catabolic process |  |
|  | GO:0004180 | 0.0006504 | Down | 2 | 16 | MF carboxypeptidase activity |  |
|  | GO:0003824 | 0.0007347 | Down | 18 | 3370 | MF catalytic activity |  |
|  | GO:0046348 | 0.0007466 | Down | 2 | 16 | BP amino sugar catabolic process |  |
|  | GO:0006672 | 0.0009502 | Down | 2 | 18 | BP ceramide metabolic process |  |
|  | GO:0000272 | 0.0011508 | Down | 2 | 20 | BP polysaccharide catabolic process |  |
|  | GO:0019538 | 0.0012699 | Down | 10 | 1313 | BP protein metabolic process |  |
|  | GO:0006026 | 0.0013129 | Down | 2 | 23 | BP aminoglycan catabolic process |  |
|  | GO:0016042 | 0.0014212 | Down | 3 | 96 | BP lipid catabolic process |  |
|  | GO:0006030 | 0.0020232 | Down | 2 | 27 | BP chitin metabolic process |  |
|  | GO:0044707 | 0.0021718 | Down | 8 | 917 | BP single-multicellular organism process |  |
|  | GO:0035187 | 0.0028325 | Down | 1 | 1 | BP hatching behavior |  |
|  | GO:1901071 | 0.0030683 | Down | 2 | 33 | BP glucosamine-containing compound metabolic process |  |
|  | GO:0006665 | 0.0033273 | Down | 2 | 35 | BP sphingolipid metabolic process |  |
|  | GO:0044238 | 0.0036009 | Down | 18 | 3953 | BP primary metabolic process |  |
|  | GO:0006040 | 0.0038543 | Down | 2 | 37 | BP amino sugar metabolic process |  |
|  | GO:0017040 | 0.0048047 | Down | 1 | 2 | MF ceramidase activity |  |
|  | GO:0006006 | 0.0056033 | Down | 2 | 44 | BP glucose metabolic process |  |
|  | GO:0071704 | 0.005781 | Down | 18 | 4141 | BP organic substance metabolic process |  |
|  | GO:0008152 | 0.0059966 | Down | 19 | 4493 | BP metabolic process |  |
|  | GO:0005976 | 0.0064691 | Down | 2 | 46 | BP polysaccharide metabolic process |  |
|  | GO:0044706 | 0.0068052 | Down | 2 | 54 | BP multi-multicellular organism process |  |
|  | GO:1901565 | 0.0074702 | Down | 4 | 280 | BP organonitrogen compound catabolic process |  |
|  | GO:0044703 | 0.008316 | Down | 2 | 59 | BP multi-organism reproductive process |  |
|  | GO:0006022 | 0.0085897 | Down | 2 | 58 | BP aminoglycan metabolic process |  |
|  | GO:0044242 | 0.0087834 | Down | 2 | 58 | BP cellular lipid catabolic process |  |
|  | GO:0008456 | 0.0097569 | Down | 1 | 2 | MF alpha-N-acetylgalactosaminidase activity |  |
|  | GO:0008241 | 0.0103262 | Down | 1 | 5 | MF peptidyl-dipeptidase activity |  |
|  | GO:0004767 | 0.0116315 | Down | 1 | 4 | MF sphingomyelin phosphodiesterase activity |  |
|  | GO:0006685 | 0.0116315 | Down | 1 | 4 | BP sphingomyelin catabolic process |  |
|  | GO:0032682 | 0.0121729 | Down | 1 | 6 | BP negative regulation of chemokine production |  |
|  | GO:0034442 | 0.0121729 | Down | 1 | 6 | BP regulation of lipoprotein oxidation |  |
|  | GO:0034443 | 0.0121729 | Down | 1 | 6 | BP negative regulation of lipoprotein oxidation |  |
|  | GO:0050746 | 0.0121729 | Down | 1 | 6 | BP regulation of lipoprotein metabolic process |  |
|  | GO:0050748 | 0.0121729 | Down | 1 | 6 | BP negative regulation of lipoprotein metabolic process |  |
|  | GO:0060587 | 0.0121729 | Down | 1 | 6 | BP regulation of lipoprotein lipid oxidation |  |
|  | GO:0060588 | 0.0121729 | Down | 1 | 6 | BP negative regulation of lipoprotein lipid oxidation |  |
|  | GO:0071637 | 0.0121729 | Down | 1 | 6 | BP regulation of monocyte chemotactic protein-1 production |  |
|  | GO:0071638 | 0.0121729 | Down | 1 | 6 | BP negative regulation of monocyte chemotactic protein-1 production |  |
|  | GO:1900015 | 0.0121729 | Down | 1 | 6 | BP regulation of cytokine production involved in inflammatory response | |
|  | GO:1900016 | 0.0121729 | Down | 1 | 6 | BP negative regulation of cytokine production involved in inflammatory response | |
|  | GO:2000097 | 0.0121729 | Down | 1 | 6 | BP regulation of smooth muscle cell-matrix adhesion |  |
|  | GO:2000098 | 0.0121729 | Down | 1 | 6 | BP negative regulation of smooth muscle cell-matrix adhesion |  |
|  | GO:2000401 | 0.0121729 | Down | 1 | 6 | BP regulation of lymphocyte migration |  |
|  | GO:2000402 | 0.0121729 | Down | 1 | 6 | BP negative regulation of lymphocyte migration |  |
|  | GO:2000404 | 0.0121729 | Down | 1 | 6 | BP regulation of T cell migration |  |
|  | GO:2000405 | 0.0121729 | Down | 1 | 6 | BP negative regulation of T cell migration |  |
|  | GO:0004344 | 0.0122433 | Down | 1 | 6 | MF glucose dehydrogenase activity |  |
|  | GO:0008364 | 0.0122433 | Down | 1 | 6 | BP pupal chitin-based cuticle development |  |
|  | GO:0046692 | 0.0122433 | Down | 1 | 6 | BP sperm competition |  |
|  | GO:0046693 | 0.0122433 | Down | 1 | 6 | BP sperm storage |  |
|  | GO:0006643 | 0.0122897 | Down | 2 | 71 | BP membrane lipid metabolic process |  |
|  | GO:0032450 | 0.0131507 | Down | 1 | 4 | MF maltose alpha-glucosidase activity |  |
|  | GO:0019318 | 0.0131586 | Down | 2 | 72 | BP hexose metabolic process |  |
|  | GO:0008238 | 0.0134421 | Down | 2 | 69 | MF exopeptidase activity |  |
|  | GO:0046514 | 0.0141254 | Down | 1 | 5 | BP ceramide catabolic process |  |
|  | GO:0014012 | 0.0141384 | Down | 1 | 7 | BP peripheral nervous system axon regeneration |  |
|  | GO:0031103 | 0.0141384 | Down | 1 | 7 | BP axon regeneration |  |
|  | GO:0004563 | 0.0141507 | Down | 1 | 5 | MF beta-N-acetylhexosaminidase activity |  |
|  | GO:0051895 | 0.0142312 | Down | 1 | 7 | BP negative regulation of focal adhesion assembly |  |
|  | GO:1901889 | 0.0142312 | Down | 1 | 7 | BP negative regulation of cell junction assembly |  |
|  | GO:1903392 | 0.0142312 | Down | 1 | 7 | BP negative regulation of adherens junction organization |  |
|  | GO:0022626 | 0.0160422 | Down | 1 | 8 | CC cytosolic ribosome |  |
|  | GO:0004558 | 0.016261 | Down | 1 | 5 | MF alpha-1,4-glucosidase activity |  |
|  | GO:0048662 | 0.0162743 | Down | 1 | 8 | BP negative regulation of smooth muscle cell proliferation |  |
|  | GO:0042337 | 0.0165017 | Down | 1 | 8 | BP cuticle development involved in chitin-based cuticle molting cycle |  |
|  | GO:0016052 | 0.0167856 | Down | 2 | 70 | BP carbohydrate catabolic process |  |
|  | GO:0042246 | 0.0167922 | Down | 1 | 8 | BP tissue regeneration |  |
|  | GO:0001953 | 0.0174752 | Down | 1 | 9 | BP negative regulation of cell-matrix adhesion |  |
|  | GO:0002686 | 0.0176349 | Down | 1 | 7 | BP negative regulation of leukocyte migration |  |
|  | GO:0006684 | 0.0178332 | Down | 1 | 7 | BP sphingomyelin metabolic process |  |
|  | GO:0031629 | 0.0180445 | Down | 1 | 4 | BP synaptic vesicle fusion to presynaptic active zone membrane |  |
|  | GO:0005996 | 0.0181441 | Down | 2 | 86 | BP monosaccharide metabolic process |  |
|  | GO:0006629 | 0.0182481 | Down | 4 | 410 | BP lipid metabolic process |  |
|  | GO:0016079 | 0.0187675 | Down | 1 | 7 | BP synaptic vesicle exocytosis |  |
|  | GO:0004181 | 0.0195219 | Down | 1 | 8 | MF metallocarboxypeptidase activity |  |
|  | GO:0010640 | 0.0204514 | Down | 1 | 10 | BP regulation of platelet-derived growth factor receptor signaling pathway | |
|  | GO:0010642 | 0.0204514 | Down | 1 | 10 | BP negative regulation of platelet-derived growth factor receptor signaling pathway | |
|  | GO:0031102 | 0.0217539 | Down | 1 | 9 | BP neuron projection regeneration |  |
|  | GO:0032642 | 0.0222875 | Down | 1 | 11 | BP regulation of chemokine production |  |
|  | GO:0090599 | 0.0223494 | Down | 1 | 7 | MF alpha-glucosidase activity |  |
|  | GO:0009395 | 0.0227486 | Down | 1 | 9 | BP phospholipid catabolic process |  |
|  | GO:0002685 | 0.0233138 | Down | 1 | 9 | BP regulation of leukocyte migration |  |
|  | GO:0008237 | 0.0242455 | Down | 2 | 97 | MF metallopeptidase activity |  |
|  | GO:0048678 | 0.0243411 | Down | 1 | 11 | BP response to axon injury |  |
|  | GO:0035307 | 0.0244118 | Down | 1 | 8 | BP positive regulation of protein dephosphorylation |  |
|  | GO:0046513 | 0.0262166 | Down | 1 | 11 | BP ceramide biosynthetic process |  |
|  | GO:0035304 | 0.0268408 | Down | 1 | 9 | BP regulation of protein dephosphorylation |  |
|  | GO:0045833 | 0.0271783 | Down | 1 | 11 | BP negative regulation of lipid metabolic process |  |
|  | GO:0004568 | 0.0278196 | Down | 1 | 11 | MF chitinase activity |  |
|  | GO:0035306 | 0.0280085 | Down | 1 | 10 | BP positive regulation of dephosphorylation |  |
|  | GO:0015926 | 0.0282337 | Down | 1 | 9 | MF glucosidase activity |  |
|  | GO:0045494 | 0.0282379 | Down | 1 | 14 | BP photoreceptor cell maintenance |  |
|  | GO:0048660 | 0.029096 | Down | 1 | 15 | BP regulation of smooth muscle cell proliferation |  |
|  | GO:0023021 | 0.0316501 | Down | 1 | 11 | BP termination of signal transduction |  |
|  | GO:0010812 | 0.0317122 | Down | 1 | 15 | BP negative regulation of cell-substrate adhesion |  |
|  | GO:0061564 | 0.0330392 | Down | 1 | 11 | BP axon development |  |
|  | GO:0008812 | 0.0339588 | Down | 1 | 18 | MF choline dehydrogenase activity |  |
|  | GO:1901575 | 0.0350189 | Down | 5 | 686 | BP organic substance catabolic process |  |
|  | GO:0042308 | 0.037665 | Down | 1 | 17 | BP negative regulation of protein import into nucleus |  |
|  | GO:0004620 | 0.0382645 | Down | 1 | 18 | MF phospholipase activity |  |
|  | GO:0051893 | 0.0393967 | Down | 1 | 12 | BP regulation of focal adhesion assembly |  |
|  | GO:0090109 | 0.0393967 | Down | 1 | 12 | BP regulation of cell-substrate junction assembly |  |
|  | GO:1901888 | 0.0393967 | Down | 1 | 12 | BP regulation of cell junction assembly |  |
|  | GO:1903391 | 0.0393967 | Down | 1 | 12 | BP regulation of adherens junction organization |  |
|  | GO:0008235 | 0.0410959 | Down | 1 | 18 | MF metalloexopeptidase activity |  |
|  | GO:0008343 | 0.0417077 | Down | 1 | 15 | BP adult feeding behavior |  |
|  | GO:0015485 | 0.0424546 | Down | 1 | 19 | MF cholesterol binding |  |
|  | GO:0042595 | 0.042556 | Down | 1 | 16 | BP behavioral response to starvation |  |
|  | GO:0043407 | 0.0425626 | Down | 1 | 16 | BP negative regulation of MAP kinase activity |  |
|  | GO:0006906 | 0.0429626 | Down | 1 | 15 | BP vesicle fusion |  |
|  | GO:0042439 | 0.0433386 | Down | 1 | 19 | BP ethanolamine-containing compound metabolic process |  |
|  | GO:0032934 | 0.0436052 | Down | 1 | 20 | MF sterol binding |  |
|  | GO:1900181 | 0.0443031 | Down | 1 | 20 | BP negative regulation of protein localization to nucleus |  |
|  | GO:0046823 | 0.0458903 | Down | 1 | 21 | BP negative regulation of nucleocytoplasmic transport |  |
|  | GO:0006066 | 0.0465206 | Down | 2 | 140 | BP alcohol metabolic process |  |
|  | GO:0050728 | 0.0467902 | Down | 1 | 23 | BP negative regulation of inflammatory response |  |
|  | GO:0046164 | 0.0475286 | Down | 1 | 21 | BP alcohol catabolic process |  |
|  | GO:1901616 | 0.0475286 | Down | 1 | 21 | BP organic hydroxy compound catabolic process |  |
|  | GO:0030148 | 0.0476921 | Down | 1 | 22 | BP sphingolipid biosynthetic process |  |
|  | GO:0001952 | 0.0490656 | Down | 1 | 18 | BP regulation of cell-matrix adhesion |  |
|  | GO:0008150 | 0.0492449 | Down | 22 | 6618 | BP biological_process |  |
| Control vs. Cold | |  |  |  |  |  |  |
|  | GO:0045735 | 0.0001148 | Up | 2 | 10 | MF nutrient reservoir activity |  |
|  | GO:0005319 | 0.0011129 | Up | 2 | 43 | MF lipid transporter activity |  |
|  | GO:0006869 | 0.0059608 | Up | 2 | 105 | BP lipid transport |  |
|  | GO:0005576 | 0.000528 | Up | 4 | 382 | CC extracellular region |  |
|  | GO:0022892 | 0.0093865 | Up | 2 | 438 | MF substrate-specific transporter activity |  |
|  | GO:0005215 | 0.017078 | Up | 3 | 562 | MF transporter activity |  |
|  | GO:0071702 | 0.0244447 | Up | 2 | 713 | BP organic substance transport |  |
|  | GO:0004252 | 5.16E-09 | Down | 5 | 62 | MF serine-type endopeptidase activity |  |
|  | GO:0008236 | 1.23E-08 | Down | 5 | 74 | MF serine-type peptidase activity |  |
|  | GO:0017171 | 1.41E-08 | Down | 5 | 76 | MF serine hydrolase activity |  |
|  | GO:0005615 | 4.50E-08 | Down | 6 | 191 | CC extracellular space |  |
|  | GO:0030574 | 7.44E-08 | Down | 3 | 9 | BP collagen catabolic process |  |
|  | GO:0044243 | 7.44E-08 | Down | 3 | 9 | BP multicellular organism catabolic process |  |
|  | GO:0032963 | 1.40E-07 | Down | 3 | 11 | BP collagen metabolic process |  |
|  | GO:0044236 | 1.40E-07 | Down | 3 | 11 | BP multicellular organism metabolic process |  |
|  | GO:0044259 | 1.40E-07 | Down | 3 | 11 | BP multicellular organismal macromolecule metabolic process |  |
|  | GO:0004175 | 1.54E-06 | Down | 5 | 201 | MF endopeptidase activity |  |
|  | GO:0006508 | 1.45E-05 | Down | 5 | 298 | **BP proteolysis** |  |
|  | GO:0070011 | 1.89E-05 | Down | 5 | 325 | MF peptidase activity, acting on L-amino acid peptides |  |
|  | GO:0008233 | 2.28E-05 | Down | 5 | 339 | MF peptidase activity |  |
|  | GO:0044421 | 6.67E-05 | Down | 6 | 680 | CC extracellular region part |  |
|  | GO:0006006 | 0.0008787 | Down | 2 | 41 | BP glucose metabolic process |  |
|  | GO:0044712 | 0.0011775 | Down | 4 | 437 | BP single-organism catabolic process |  |
|  | GO:0016787 | 0.0022251 | Down | 6 | 1342 | MF hydrolase activity |  |
|  | GO:0019318 | 0.002236 | Down | 2 | 69 | BP hexose metabolic process |  |
|  | GO:0032501 | 0.0024029 | Down | 5 | 935 | BP multicellular organismal process |  |
|  | GO:0005996 | 0.0031314 | Down | 2 | 82 | BP monosaccharide metabolic process |  |
|  | GO:0044710 | 0.0033334 | Down | 7 | 2013 | BP single-organism metabolic process |  |
|  | GO:0030682 | 0.0040909 | Down | 1 | 5 | BP evasion or tolerance of host defense response |  |
|  | GO:0044413 | 0.0040909 | Down | 1 | 5 | BP avoidance of host defenses |  |
|  | GO:0044415 | 0.0040909 | Down | 1 | 5 | BP evasion or tolerance of host defenses |  |
|  | GO:0051807 | 0.0040909 | Down | 1 | 5 | BP evasion or tolerance of defense response of other organism involved in symbiotic interaction | |
|  | GO:0051832 | 0.0040909 | Down | 1 | 5 | BP avoidance of defenses of other organism involved in symbiotic interaction | |
|  | GO:0051834 | 0.0040909 | Down | 1 | 5 | BP evasion or tolerance of defenses of other organism involved in symbiotic interaction | |
|  | GO:0052173 | 0.0049754 | Down | 1 | 6 | BP response to defenses of other organism involved in symbiotic interaction | |
|  | GO:0052200 | 0.0049754 | Down | 1 | 6 | BP response to host defenses |  |
|  | GO:0075136 | 0.0049754 | Down | 1 | 6 | BP response to host |  |
|  | GO:0032682 | 0.0050357 | Down | 1 | 6 | BP negative regulation of chemokine production |  |
|  | GO:0034442 | 0.0050357 | Down | 1 | 6 | BP regulation of lipoprotein oxidation |  |
|  | GO:0034443 | 0.0050357 | Down | 1 | 6 | BP negative regulation of lipoprotein oxidation |  |
|  | GO:0050746 | 0.0050357 | Down | 1 | 6 | BP regulation of lipoprotein metabolic process |  |
|  | GO:0050748 | 0.0050357 | Down | 1 | 6 | BP negative regulation of lipoprotein metabolic process |  |
|  | GO:0060587 | 0.0050357 | Down | 1 | 6 | BP regulation of lipoprotein lipid oxidation |  |
|  | GO:0060588 | 0.0050357 | Down | 1 | 6 | BP negative regulation of lipoprotein lipid oxidation |  |
|  | GO:0071637 | 0.0050357 | Down | 1 | 6 | BP regulation of monocyte chemotactic protein-1 production |  |
|  | GO:0071638 | 0.0050357 | Down | 1 | 6 | BP negative regulation of monocyte chemotactic protein-1 production |  |
|  | GO:1900015 | 0.0050357 | Down | 1 | 6 | BP regulation of cytokine production involved in inflammatory response | |
|  | GO:1900016 | 0.0050357 | Down | 1 | 6 | BP negative regulation of cytokine production involved in inflammatory response | |
|  | GO:2000097 | 0.0050357 | Down | 1 | 6 | BP regulation of smooth muscle cell-matrix adhesion |  |
|  | GO:2000098 | 0.0050357 | Down | 1 | 6 | BP negative regulation of smooth muscle cell-matrix adhesion |  |
|  | GO:2000401 | 0.0050357 | Down | 1 | 6 | BP regulation of lymphocyte migration |  |
|  | GO:2000402 | 0.0050357 | Down | 1 | 6 | BP negative regulation of lymphocyte migration |  |
|  | GO:2000404 | 0.0050357 | Down | 1 | 6 | BP regulation of T cell migration |  |
|  | GO:2000405 | 0.0050357 | Down | 1 | 6 | BP negative regulation of T cell migration |  |
|  | GO:0014012 | 0.0058562 | Down | 1 | 7 | BP peripheral nervous system axon regeneration |  |
|  | GO:0031103 | 0.0058562 | Down | 1 | 7 | BP axon regeneration |  |
|  | GO:0051895 | 0.0058776 | Down | 1 | 7 | BP negative regulation of focal adhesion assembly |  |
|  | GO:1901889 | 0.0058776 | Down | 1 | 7 | BP negative regulation of cell junction assembly |  |
|  | GO:1903392 | 0.0058776 | Down | 1 | 7 | BP negative regulation of adherens junction organization |  |
|  | GO:0008150 | 0.0060403 | Down | 13 | 6564 | BP biological_process |  |
|  | GO:0004344 | 0.0060927 | Down | 1 | 7 | MF glucose dehydrogenase activity |  |
|  | GO:0008364 | 0.0060927 | Down | 1 | 7 | BP pupal chitin-based cuticle development |  |
|  | GO:0046692 | 0.0060927 | Down | 1 | 7 | BP sperm competition |  |
|  | GO:0046693 | 0.0060927 | Down | 1 | 7 | BP sperm storage |  |
|  | GO:0002686 | 0.0069177 | Down | 1 | 7 | BP negative regulation of leukocyte migration |  |
|  | GO:0022626 | 0.0069364 | Down | 1 | 8 | CC cytosolic ribosome |  |
|  | GO:0007586 | 0.0069506 | Down | 1 | 7 | BP digestion |  |
|  | GO:0042246 | 0.0071341 | Down | 1 | 8 | BP tissue regeneration |  |
|  | GO:0042337 | 0.0071734 | Down | 1 | 8 | BP cuticle development involved in chitin-based cuticle molting cycle |  |
|  | GO:0001953 | 0.0073532 | Down | 1 | 9 | BP negative regulation of cell-matrix adhesion |  |
|  | GO:0048662 | 0.0075103 | Down | 1 | 9 | BP negative regulation of smooth muscle cell proliferation |  |
|  | GO:0044699 | 0.0078547 | Down | 10 | 4333 | BP single-organism process |  |
|  | GO:0009056 | 0.0083737 | Down | 4 | 766 | BP catabolic process |  |
|  | GO:0031102 | 0.0088151 | Down | 1 | 9 | BP neuron projection regeneration |  |
|  | GO:0019538 | 0.0090769 | Down | 5 | 1295 | BP protein metabolic process |  |
|  | GO:0002685 | 0.009326 | Down | 1 | 9 | BP regulation of leukocyte migration |  |
|  | GO:0032642 | 0.0094092 | Down | 1 | 11 | BP regulation of chemokine production |  |
|  | GO:0010640 | 0.0094762 | Down | 1 | 11 | BP regulation of platelet-derived growth factor receptor signaling pathway | |
|  | GO:0010642 | 0.0094762 | Down | 1 | 11 | BP negative regulation of platelet-derived growth factor receptor signaling pathway | |
|  | GO:0048678 | 0.0103029 | Down | 1 | 11 | BP response to axon injury |  |
|  | GO:0045833 | 0.0108428 | Down | 1 | 11 | BP negative regulation of lipid metabolic process |  |
|  | GO:0048660 | 0.0125303 | Down | 1 | 15 | BP regulation of smooth muscle cell proliferation |  |
|  | GO:0061564 | 0.0125489 | Down | 1 | 11 | BP axon development |  |
|  | GO:0051893 | 0.0133679 | Down | 1 | 12 | BP regulation of focal adhesion assembly |  |
|  | GO:0090109 | 0.0133679 | Down | 1 | 12 | BP regulation of cell-substrate junction assembly |  |
|  | GO:1901888 | 0.0133679 | Down | 1 | 12 | BP regulation of cell junction assembly |  |
|  | GO:1903391 | 0.0133679 | Down | 1 | 12 | BP regulation of adherens junction organization |  |
|  | GO:0010812 | 0.0134138 | Down | 1 | 15 | BP negative regulation of cell-substrate adhesion |  |
|  | GO:0044707 | 0.0135527 | Down | 4 | 911 | BP single-multicellular organism process |  |
|  | GO:0003824 | 0.0145434 | Down | 8 | 3359 | MF catalytic activity |  |
|  | GO:0015485 | 0.0155676 | Down | 1 | 17 | MF cholesterol binding |  |
|  | GO:0042308 | 0.0157819 | Down | 1 | 17 | BP negative regulation of protein import into nucleus |  |
|  | GO:0032934 | 0.0161957 | Down | 1 | 18 | MF sterol binding |  |
|  | GO:0008812 | 0.0168825 | Down | 1 | 20 | MF choline dehydrogenase activity |  |
|  | GO:1900181 | 0.0180488 | Down | 1 | 19 | BP negative regulation of protein localization to nucleus |  |
|  | GO:0001952 | 0.0180933 | Down | 1 | 18 | BP regulation of cell-matrix adhesion |  |
|  | GO:0050728 | 0.019318 | Down | 1 | 22 | BP negative regulation of inflammatory response |  |
|  | GO:0046823 | 0.0193862 | Down | 1 | 21 | BP negative regulation of nucleocytoplasmic transport |  |
|  | GO:0005496 | 0.0201137 | Down | 1 | 23 | MF steroid binding |  |
|  | GO:0043178 | 0.0207304 | Down | 1 | 23 | MF alcohol binding |  |
|  | GO:0001818 | 0.0207803 | Down | 1 | 20 | BP negative regulation of cytokine production |  |
|  | GO:0051701 | 0.0212774 | Down | 1 | 24 | BP interaction with host |  |
|  | GO:1903650 | 0.022802 | Down | 1 | 24 | BP negative regulation of cytoplasmic transport |  |
|  | GO:0008152 | 0.0233878 | Down | 9 | 4471 | BP metabolic process |  |
|  | GO:0090317 | 0.0235051 | Down | 1 | 24 | BP negative regulation of intracellular protein transport |  |
|  | GO:0040003 | 0.0241147 | Down | 1 | 24 | BP chitin-based cuticle development |  |
|  | GO:0031099 | 0.0249879 | Down | 1 | 25 | BP regeneration |  |
|  | GO:1903035 | 0.0251492 | Down | 1 | 29 | BP negative regulation of response to wounding |  |
|  | GO:0042335 | 0.0268913 | Down | 1 | 28 | BP cuticle development |  |
|  | GO:0007162 | 0.0282308 | Down | 1 | 31 | BP negative regulation of cell adhesion |  |
|  | GO:0044723 | 0.0287645 | Down | 2 | 251 | BP single-organism carbohydrate metabolic process |  |
|  | GO:0050727 | 0.0291769 | Down | 1 | 31 | BP regulation of inflammatory response |  |
|  | GO:0031348 | 0.0298152 | Down | 1 | 32 | BP negative regulation of defense response |  |
|  | GO:0032387 | 0.0304845 | Down | 1 | 29 | BP negative regulation of intracellular transport |  |
|  | GO:0030336 | 0.0306286 | Down | 1 | 31 | BP negative regulation of cell migration |  |
|  | GO:2000146 | 0.0306286 | Down | 1 | 31 | BP negative regulation of cell motility |  |
|  | GO:0051271 | 0.0315474 | Down | 1 | 32 | BP negative regulation of cellular component movement |  |
|  | GO:0044238 | 0.0316943 | Down | 8 | 3930 | BP primary metabolic process |  |
|  | GO:0000302 | 0.0325222 | Down | 1 | 36 | BP response to reactive oxygen species |  |
|  | GO:0040013 | 0.032586 | Down | 1 | 33 | BP negative regulation of locomotion |  |
|  | GO:0051224 | 0.0328406 | Down | 1 | 34 | BP negative regulation of protein transport |  |
|  | GO:1903828 | 0.0353281 | Down | 1 | 36 | BP negative regulation of cellular protein localization |  |
|  | GO:0010810 | 0.0356587 | Down | 1 | 34 | BP regulation of cell-substrate adhesion |  |
|  | GO:0042306 | 0.0375692 | Down | 1 | 41 | BP regulation of protein import into nucleus |  |
|  | GO:0032102 | 0.0381541 | Down | 1 | 44 | BP negative regulation of response to external stimulus |  |
|  | GO:0031175 | 0.0396483 | Down | 1 | 36 | BP neuron projection development |  |
|  | GO:0071704 | 0.0400801 | Down | 8 | 4117 | BP organic substance metabolic process |  |
|  | GO:1900180 | 0.0405339 | Down | 1 | 44 | BP regulation of protein localization to nucleus |  |
|  | GO:0009611 | 0.0408791 | Down | 1 | 39 | BP response to wounding |  |
|  | GO:0051704 | 0.0450956 | Down | 2 | 334 | BP multi-organism process |  |
|  | GO:0007420 | 0.0461386 | Down | 1 | 49 | BP brain development |  |
|  | GO:0005975 | 0.0487005 | Down | 2 | 335 | BP carbohydrate metabolic process |  |
|  | GO:1903533 | 0.0497346 | Down | 1 | 53 | BP regulation of protein targeting |  |
|  |  |  |  |  |  |  |  |
| *Grylloblatta* sp. "North Cascades" Mt. Rainier | | | |  |  |  |  |
| Control vs Heat | |  |  |  |  |  |  |
|  | GO:0001562 | 8.24E-05 | Down | 1 | 3 | BP response to protozoan |  |
|  | GO:0042832 | 8.24E-05 | Down | 1 | 3 | BP defense response to protozoan |  |
|  | GO:0042742 | 0.0019197 | Down | 1 | 70 | BP defense response to bacterium |  |
|  | GO:0009617 | 0.0020301 | Down | 1 | 74 | BP response to bacterium |  |
|  | GO:0045087 | 0.0027396 | Down | 1 | 100 | BP innate immune response |  |
|  | GO:0098542 | 0.0029881 | Down | 1 | 109 | BP defense response to other organism |  |
|  | GO:0006955 | 0.0039203 | Down | 1 | 143 | BP immune response |  |
|  | GO:0051707 | 0.0043028 | Down | 1 | 157 | BP response to other organism |  |
|  | GO:0043207 | 0.0052354 | Down | 1 | 191 | BP response to external biotic stimulus |  |
|  | GO:0006952 | 0.0053705 | Down | 1 | 196 | BP defense response |  |
|  | GO:0009607 | 0.0054276 | Down | 1 | 198 | BP response to biotic stimulus |  |
|  | GO:0002376 | 0.0080884 | Down | 1 | 295 | BP immune system process |  |
|  | GO:0051704 | 0.0099557 | Down | 1 | 363 | BP multi-organism process |  |
|  | GO:0005576 | 0.0104535 | Down | 1 | 381 | CC extracellular region |  |
|  | GO:0009605 | 0.0106368 | Down | 1 | 388 | BP response to external stimulus |  |
|  | GO:0006950 | 0.0242749 | Down | 1 | 885 | BP response to stress | TR3410\|c1_g1: DFP_LOCMI, putative defense protein precursor |
|  | GO:0050896 | 0.0358159 | Down | 1 | 1306 | BP response to stimulus |  |
| Control vs. Cold | |  |  |  |  |  |  |
|  | GO:0008061 | 9.89E-05 | Up | 2 | 38 | MF chitin binding |  |
|  | GO:0006030 | 0.0001042 | Up | 2 | 39 | BP chitin metabolic process |  |
|  | GO:1901071 | 0.0001515 | Up | 2 | 47 | BP glucosamine-containing compound metabolic process |  |
|  | GO:0006040 | 0.0001785 | Up | 2 | 51 | BP amino sugar metabolic process |  |
|  | GO:0006022 | 0.0004807 | Up | 2 | 84 | BP aminoglycan metabolic process |  |
|  | GO:0005576 | 0.0006291 | Up | 3 | 446 | CC extracellular region |  |
|  | GO:1901135 | 0.0101572 | Up | 2 | 399 | BP carbohydrate derivative metabolic process |  |
|  | GO:0005770 | 0.0220752 | Up | 1 | 58 | CC late endosome |  |
|  | GO:1901564 | 0.0317916 | Up | 2 | 733 | BP organonitrogen compound metabolic process |  |
|  | GO:0004252 | 0.03372 | Up | 1 | 89 | MF serine-type endopeptidase activity |  |
|  | GO:0008236 | 0.0392149 | Up | 1 | 104 | MF serine-type peptidase activity |  |
|  | GO:0017171 | 0.0414644 | Up | 1 | 110 | MF serine hydrolase activity |  |
|  |  |  |  |  |  |  |  |
| *Grylloblatta* sp. "Sierra Buttes" | | |  |  |  |  |  |
| Control vs Heat | |  |  |  |  |  |  |
|  | GO:0000096 | 0.0003994 | Up | 2 | 24 | BP sulfur amino acid metabolic process |  |
|  | GO:0000097 | 0.016694 | Up | 1 | 14 | BP sulfur amino acid biosynthetic process |  |
|  | GO:0001561 | 0.0078443 | Up | 1 | 6 | BP fatty acid alpha-oxidation |  |
|  | GO:0001568 | 0.0132982 | Up | 1 | 10 | BP blood vessel development |  |
|  | GO:0001570 | 0.0170251 | Up | 1 | 13 | BP vasculogenesis |  |
|  | GO:0003674 | 0.0117316 | Up | 18 | 8504 | MF molecular_function |  |
|  | GO:0003824 | 0.0001148 | Up | 15 | 4100 | MF catalytic activity |  |
|  | GO:0004341 | 0.004069 | Up | 1 | 3 | MF gluconolactonase activity |  |
|  | GO:0004497 | 1.01E-05 | Up | 4 | 118 | MF monooxygenase activity |  |
|  | GO:0004505 | 0.0013604 | Up | 1 | 1 | MF phenylalanine 4-monooxygenase activity |  |
|  | GO:0004510 | 0.0013604 | Up | 1 | 1 | MF tryptophan 5-monooxygenase activity |  |
|  | GO:0004558 | 0.0105234 | Up | 1 | 7 | MF alpha-1,4-glucosidase activity |  |
|  | GO:0004714 | 0.0347895 | Up | 1 | 27 | MF transmembrane receptor protein tyrosine kinase activity |  |
|  | GO:0004731 | 0.0027283 | Up | 1 | 2 | MF purine-nucleoside phosphorylase activity |  |
|  | GO:0004806 | 0.021218 | Up | 1 | 18 | MF triglyceride lipase activity |  |
|  | GO:0005506 | 1.80E-06 | Up | 5 | 164 | MF iron ion binding |  |
|  | GO:0005615 | 0.0334572 | Up | 2 | 225 | CC extracellular space |  |
|  | GO:0005789 | 0.0131852 | Up | 3 | 388 | CC endoplasmic reticulum membrane |  |
|  | GO:0006082 | 0.0003193 | Up | 5 | 454 | BP organic acid metabolic process |  |
|  | GO:0006520 | 0.0024233 | Up | 3 | 201 | BP cellular amino acid metabolic process |  |
|  | GO:0006534 | 0.0089082 | Up | 1 | 6 | BP cysteine metabolic process |  |
|  | GO:0006555 | 0.0134084 | Up | 1 | 11 | BP methionine metabolic process |  |
|  | GO:0006558 | 0.0099626 | Up | 1 | 7 | BP L-phenylalanine metabolic process |  |
|  | GO:0006559 | 0.0083186 | Up | 1 | 6 | BP L-phenylalanine catabolic process |  |
|  | GO:0006577 | 0.0133198 | Up | 1 | 11 | BP amino-acid betaine metabolic process |  |
|  | GO:0006579 | 0.0021097 | Up | 1 | 2 | BP amino-acid betaine catabolic process |  |
|  | GO:0006720 | 0.0470892 | Up | 1 | 36 | BP isoprenoid metabolic process |  |
|  | GO:0006766 | 0.0400162 | Up | 1 | 34 | BP vitamin metabolic process |  |
|  | GO:0006767 | 0.0300473 | Up | 1 | 26 | BP water-soluble vitamin metabolic process |  |
|  | GO:0006790 | 0.0105391 | Up | 2 | 124 | BP sulfur compound metabolic process |  |
|  | GO:0006813 | 0.0304661 | Up | 1 | 26 | BP potassium ion transport |  |
|  | GO:0007616 | 0.0392036 | Up | 1 | 33 | BP long-term memory |  |
|  | GO:0008172 | 0.003914 | Up | 1 | 3 | MF S-methyltransferase activity |  |
|  | GO:0008898 | 0.0027107 | Up | 1 | 2 | MF S-adenosylmethionine-homocysteine S-methyltransferase activity |  |
|  | GO:0009056 | 0.0303625 | Up | 4 | 914 | BP catabolic process |  |
|  | GO:0009062 | 0.0497276 | Up | 1 | 38 | BP fatty acid catabolic process |  |
|  | GO:0009066 | 0.0374273 | Up | 1 | 30 | BP aspartate family amino acid metabolic process |  |
|  | GO:0009067 | 0.0199603 | Up | 1 | 16 | BP aspartate family amino acid biosynthetic process |  |
|  | GO:0009069 | 0.0306537 | Up | 1 | 22 | BP serine family amino acid metabolic process |  |
|  | GO:0009072 | 0.0204999 | Up | 1 | 14 | BP aromatic amino acid family metabolic process |  |
|  | GO:0009074 | 0.0131819 | Up | 1 | 10 | BP aromatic amino acid family catabolic process |  |
|  | GO:0009086 | 0.01167 | Up | 1 | 10 | BP methionine biosynthetic process |  |
|  | GO:0009110 | 0.01158 | Up | 1 | 10 | BP vitamin biosynthetic process |  |
|  | GO:0009612 | 0.0454107 | Up | 1 | 36 | BP response to mechanical stimulus |  |
|  | GO:0015108 | 0.0399902 | Up | 1 | 37 | MF chloride transmembrane transporter activity |  |
|  | GO:0015294 | 0.030909 | Up | 1 | 26 | MF solute:cation symporter activity |  |
|  | GO:0015296 | 0.0161691 | Up | 1 | 14 | MF anion:cation symporter activity |  |
|  | GO:0015377 | 0.0079704 | Up | 1 | 8 | MF cation:chloride symporter activity |  |
|  | GO:0015926 | 0.0190389 | Up | 1 | 15 | MF glucosidase activity |  |
|  | GO:0016042 | 0.0093171 | Up | 2 | 116 | BP lipid catabolic process |  |
|  | GO:0016053 | 0.0102104 | Up | 2 | 116 | BP organic acid biosynthetic process |  |
|  | GO:0016054 | 0.0081034 | Up | 2 | 104 | BP organic acid catabolic process |  |
|  | GO:0016298 | 0.0460493 | Up | 1 | 41 | MF lipase activity |  |
|  | GO:0016491 | 9.48E-06 | Up | 7 | 594 | MF oxidoreductase activity |  |
|  | GO:0016525 | 0.0149184 | Up | 1 | 10 | BP negative regulation of angiogenesis |  |
|  | GO:0016597 | 0.0302333 | Up | 1 | 23 | MF amino acid binding |  |
|  | GO:0016701 | 0.016957 | Up | 1 | 12 | MF oxidoreductase activity, acting on single donors with incorporation of molecular oxygen | |
|  | GO:0016702 | 0.014267 | Up | 1 | 10 | MF oxidoreductase activity, acting on single donors with incorporation of molecular oxygen, incorporation of two atoms of oxygen | |
|  | GO:0016705 | 1.22E-06 | Up | 5 | 157 | MF oxidoreductase activity, acting on paired donors, with incorporation or reduction of molecular oxygen | |
|  | GO:0016706 | 0.0399709 | Up | 1 | 31 | MF oxidoreductase activity, acting on paired donors, with incorporation or reduction of molecular oxygen, 2-oxoglutarate as one donor, and incorporation of one atom each of oxygen into both donors | |
|  | GO:0016714 | 0.0027994 | Up | 1 | 2 | MF oxidoreductase activity, acting on paired donors, with incorporation or reduction of molecular oxygen, reduced pteridine as one donor, and incorporation of one atom of oxygen | |
|  | GO:0016757 | 0.0158649 | Up | 2 | 156 | MF transferase activity, transferring glycosyl groups |  |
|  | GO:0016763 | 0.0285403 | Up | 1 | 24 | MF transferase activity, transferring pentosyl groups |  |
|  | GO:0016788 | 0.0391417 | Up | 3 | 585 | MF hydrolase activity, acting on ester bonds |  |
|  | GO:0017172 | 0.0012857 | Up | 1 | 1 | MF cysteine dioxygenase activity |  |
|  | GO:0019199 | 0.0405982 | Up | 1 | 33 | MF transmembrane receptor protein kinase activity |  |
|  | GO:0019395 | 0.0464974 | Up | 1 | 36 | BP fatty acid oxidation |  |
|  | GO:0019530 | 0.008367 | Up | 1 | 7 | BP taurine metabolic process |  |
|  | GO:0019752 | 0.0002357 | Up | 5 | 424 | BP carboxylic acid metabolic process |  |
|  | GO:0019852 | 0.0076991 | Up | 1 | 6 | BP L-ascorbic acid metabolic process |  |
|  | GO:0019853 | 0.0067819 | Up | 1 | 5 | BP L-ascorbic acid biosynthetic process |  |
|  | GO:0020037 | 0.0006674 | Up | 3 | 144 | MF heme binding |  |
|  | GO:0030336 | 0.0481678 | Up | 1 | 40 | BP negative regulation of cell migration |  |
|  | GO:0031406 | 0.0018074 | Up | 2 | 47 | MF carboxylic acid binding |  |
|  | GO:0031418 | 0.0160965 | Up | 1 | 11 | MF L-ascorbic acid binding |  |
|  | GO:0032450 | 0.0086205 | Up | 1 | 6 | MF maltose alpha-glucosidase activity |  |
|  | GO:0032526 | 0.0210547 | Up | 1 | 16 | BP response to retinoic acid |  |
|  | GO:0032781 | 0.0184719 | Up | 1 | 16 | BP positive regulation of ATPase activity |  |
|  | GO:0033477 | 0.0027107 | Up | 1 | 2 | BP S-methylmethionine metabolic process |  |
|  | GO:0033993 | 0.0102942 | Up | 2 | 112 | BP response to lipid |  |
|  | GO:0035264 | 0.0288991 | Up | 1 | 24 | BP multicellular organism growth |  |
|  | GO:0042364 | 0.01158 | Up | 1 | 10 | BP water-soluble vitamin biosynthetic process |  |
|  | GO:0042412 | 0.003904 | Up | 1 | 3 | BP taurine biosynthetic process |  |
|  | GO:0042427 | 0.0027776 | Up | 1 | 2 | BP serotonin biosynthetic process |  |
|  | GO:0042428 | 0.0095186 | Up | 1 | 7 | BP serotonin metabolic process |  |
|  | GO:0042430 | 0.0186674 | Up | 1 | 13 | BP indole-containing compound metabolic process |  |
|  | GO:0042435 | 0.0078739 | Up | 1 | 6 | BP indole-containing compound biosynthetic process |  |
|  | GO:0043094 | 0.020878 | Up | 1 | 19 | BP cellular metabolic compound salvage |  |
|  | GO:0043102 | 0.0077977 | Up | 1 | 7 | BP amino acid salvage |  |
|  | GO:0043167 | 0.000716 | Up | 13 | 3883 | MF ion binding |  |
|  | GO:0043169 | 9.79E-05 | Up | 12 | 2763 | MF cation binding |  |
|  | GO:0043177 | 0.0018074 | Up | 2 | 47 | MF organic acid binding |  |
|  | GO:0043436 | 0.0003075 | Up | 5 | 450 | BP oxoacid metabolic process |  |
|  | GO:0043462 | 0.0246614 | Up | 1 | 20 | BP regulation of ATPase activity |  |
|  | GO:0044272 | 0.0021298 | Up | 2 | 55 | BP sulfur compound biosynthetic process |  |
|  | GO:0044281 | 0.0026384 | Up | 6 | 1093 | BP small molecule metabolic process |  |
|  | GO:0044282 | 0.014074 | Up | 2 | 140 | BP small molecule catabolic process |  |
|  | GO:0044283 | 0.0217151 | Up | 2 | 178 | BP small molecule biosynthetic process |  |
|  | GO:0044432 | 0.0279919 | Up | 3 | 505 | CC endoplasmic reticulum part |  |
|  | GO:0044710 | 0.0030501 | Up | 9 | 2413 | BP single-organism metabolic process |  |
|  | GO:0044712 | 0.02888 | Up | 3 | 512 | BP single-organism catabolic process |  |
|  | GO:0044801 | 0.04976 | Up | 1 | 36 | BP single-organism membrane fusion |  |
|  | GO:0045026 | 0.0034703 | Up | 1 | 2 | BP plasma membrane fusion |  |
|  | GO:0045765 | 0.0342514 | Up | 1 | 27 | BP regulation of angiogenesis |  |
|  | GO:0046189 | 0.0082247 | Up | 1 | 7 | BP phenol-containing compound biosynthetic process |  |
|  | GO:0046364 | 0.0298229 | Up | 1 | 23 | BP monosaccharide biosynthetic process |  |
|  | GO:0046394 | 0.0102104 | Up | 2 | 116 | BP carboxylic acid biosynthetic process |  |
|  | GO:0046395 | 0.0081034 | Up | 2 | 104 | BP carboxylic acid catabolic process |  |
|  | GO:0046439 | 0.0045089 | Up | 1 | 3 | BP L-cysteine metabolic process |  |
|  | GO:0046872 | 0.0003968 | Up | 11 | 2743 | MF metal ion binding |  |
|  | GO:0046906 | 0.0006877 | Up | 3 | 145 | MF tetrapyrrole binding |  |
|  | GO:0046914 | 1.91E-05 | Up | 8 | 924 | MF transition metal ion binding |  |
|  | GO:0047150 | 0.0013007 | Up | 1 | 1 | MF betaine-homocysteine S-methyltransferase activity |  |
|  | GO:0048029 | 0.0351362 | Up | 1 | 27 | MF monosaccharide binding |  |
|  | GO:0048244 | 0.0028304 | Up | 1 | 2 | MF phytanoyl-CoA dioxygenase activity |  |
|  | GO:0048754 | 0.047133 | Up | 1 | 36 | BP branching morphogenesis of an epithelial tube |  |
|  | GO:0050848 | 0.0116894 | Up | 1 | 9 | BP regulation of calcium-mediated signaling |  |
|  | GO:0050877 | 0.0423092 | Up | 2 | 260 | BP neurological system process |  |
|  | GO:0050906 | 0.0289947 | Up | 1 | 23 | BP detection of stimulus involved in sensory perception |  |
|  | GO:0050910 | 0.004446 | Up | 1 | 4 | BP detection of mechanical stimulus involved in sensory perception of sound | |
|  | GO:0050974 | 0.0160557 | Up | 1 | 13 | BP detection of mechanical stimulus involved in sensory perception |  |
|  | GO:0050982 | 0.0213817 | Up | 1 | 17 | BP detection of mechanical stimulus |  |
|  | GO:0051213 | 0.0017492 | Up | 2 | 46 | MF dioxygenase activity |  |
|  | GO:0051336 | 0.0472543 | Up | 2 | 271 | BP regulation of hydrolase activity |  |
|  | GO:0052689 | 0.0045337 | Up | 2 | 84 | MF carboxylic ester hydrolase activity |  |
|  | GO:0055114 | 0.000354 | Up | 5 | 469 | BP oxidation-reduction process |  |
|  | GO:0060444 | 0.0075211 | Up | 1 | 7 | BP branching involved in mammary gland duct morphogenesis |  |
|  | GO:0060763 | 0.0019342 | Up | 1 | 2 | BP mammary duct terminal end bud growth |  |
|  | GO:0061138 | 0.0484917 | Up | 1 | 37 | BP morphogenesis of a branching epithelium |  |
|  | GO:0061627 | 0.0013007 | Up | 1 | 1 | MF S-methylmethionine-homocysteine S-methyltransferase activity |  |
|  | GO:0071265 | 0.0077977 | Up | 1 | 7 | BP L-methionine biosynthetic process |  |
|  | GO:0071267 | 0.0077977 | Up | 1 | 7 | BP L-methionine salvage |  |
|  | GO:0090599 | 0.0157266 | Up | 1 | 12 | MF alpha-glucosidase activity |  |
|  | GO:0097089 | 0.0028304 | Up | 1 | 2 | BP methyl-branched fatty acid metabolic process |  |
|  | GO:0098589 | 0.0064944 | Up | 4 | 586 | CC membrane region |  |
|  | GO:1901160 | 0.0095186 | Up | 1 | 7 | BP primary amino compound metabolic process |  |
|  | GO:1901162 | 0.0027776 | Up | 1 | 2 | BP primary amino compound biosynthetic process |  |
|  | GO:1901342 | 0.0350337 | Up | 1 | 28 | BP regulation of vasculature development |  |
|  | GO:1901343 | 0.0149184 | Up | 1 | 10 | BP negative regulation of vasculature development |  |
|  | GO:1901564 | 0.0191773 | Up | 4 | 803 | BP organonitrogen compound metabolic process |  |
|  | GO:1901566 | 0.0050682 | Up | 3 | 277 | BP organonitrogen compound biosynthetic process |  |
|  | GO:1901575 | 0.0218522 | Up | 4 | 823 | BP organic substance catabolic process |  |
|  | GO:1901605 | 0.0005868 | Up | 3 | 119 | BP alpha-amino acid metabolic process |  |
|  | GO:1901700 | 0.0386871 | Up | 2 | 241 | BP response to oxygen-containing compound |  |
|  | GO:1902221 | 0.0099626 | Up | 1 | 7 | BP erythrose 4-phosphate/phosphoenolpyruvate family amino acid metabolic process | |
|  | GO:1902222 | 0.0083186 | Up | 1 | 6 | BP erythrose 4-phosphate/phosphoenolpyruvate family amino acid catabolic process | |
|  | GO:2000146 | 0.0481678 | Up | 1 | 40 | BP negative regulation of cell motility |  |
|  | GO:2000181 | 0.0149184 | Up | 1 | 10 | BP negative regulation of blood vessel morphogenesis |  |
|  | GO:0001952 | 0.0047842 | Down | 2 | 39 | BP regulation of cell-matrix adhesion |  |
|  | GO:0001953 | 0.0423613 | Down | 1 | 18 | BP negative regulation of cell-matrix adhesion |  |
|  | GO:0002685 | 0.0352815 | Down | 1 | 17 | BP regulation of leukocyte migration |  |
|  | GO:0002686 | 0.0262001 | Down | 1 | 11 | BP negative regulation of leukocyte migration |  |
|  | GO:0004175 | 3.10E-08 | Down | 9 | 275 | MF endopeptidase activity |  |
|  | GO:0004181 | 0.0254935 | Down | 1 | 10 | MF metallocarboxypeptidase activity |  |
|  | GO:0004252 | 3.78E-11 | Down | 8 | 99 | MF serine-type endopeptidase activity |  |
|  | GO:0005488 | 0.0221998 | Down | 23 | 6472 | MF binding |  |
|  | GO:0005496 | 0.0017897 | Down | 2 | 25 | MF steroid binding |  |
|  | GO:0005575 | 0.0035766 | Down | 31 | 8386 | CC cellular_component |  |
|  | GO:0005576 | 1.67E-06 | Down | 9 | 460 | CC extracellular region |  |
|  | GO:0005927 | 0.0433899 | Down | 1 | 14 | CC muscle tendon junction |  |
|  | GO:0005996 | 0.0218096 | Down | 2 | 96 | BP monosaccharide metabolic process |  |
|  | GO:0006022 | 0.0009838 | Down | 3 | 85 | BP aminoglycan metabolic process |  |
|  | GO:0006030 | 0.000129 | Down | 3 | 41 | BP chitin metabolic process |  |
|  | GO:0006040 | 0.000291 | Down | 3 | 56 | BP amino sugar metabolic process |  |
|  | GO:0006508 | 3.47E-07 | Down | 9 | 366 | BP proteolysis |  |
|  | GO:0006629 | 0.0010809 | Down | 6 | 489 | BP lipid metabolic process |  |
|  | GO:0007586 | 0.0007452 | Down | 2 | 18 | BP digestion |  |
|  | GO:0008061 | 6.48E-05 | Down | 3 | 32 | MF chitin binding |  |
|  | GO:0008150 | 0.0004427 | Down | 33 | 8081 | BP biological_process |  |
|  | GO:0008152 | 1.17E-05 | Down | 29 | 5519 | BP metabolic process |  |
|  | GO:0008233 | 8.91E-08 | Down | 10 | 414 | MF peptidase activity |  |
|  | GO:0008236 | 1.68E-10 | Down | 8 | 114 | MF serine-type peptidase activity |  |
|  | GO:0008270 | 0.0262947 | Down | 5 | 713 | MF zinc ion binding |  |
|  | GO:0010640 | 0.0322739 | Down | 1 | 14 | BP regulation of platelet-derived growth factor receptor signaling pathway | |
|  | GO:0010642 | 0.0306444 | Down | 1 | 13 | BP negative regulation of platelet-derived growth factor receptor signaling pathway | |
|  | GO:0010810 | 0.0091752 | Down | 2 | 54 | BP regulation of cell-substrate adhesion |  |
|  | GO:0014012 | 0.0256472 | Down | 1 | 11 | BP peripheral nervous system axon regeneration |  |
|  | GO:0015485 | 0.0013497 | Down | 2 | 21 | MF cholesterol binding |  |
|  | GO:0016787 | 1.16E-05 | Down | 15 | 1701 | MF hydrolase activity |  |
|  | GO:0017171 | 2.31E-10 | Down | 8 | 118 | MF serine hydrolase activity |  |
|  | GO:0019028 | 0.0260363 | Down | 1 | 12 | CC viral capsid |  |
|  | GO:0019538 | 0.0051042 | Down | 10 | 1592 | BP protein metabolic process |  |
|  | GO:0022626 | 0.0286558 | Down | 1 | 12 | CC cytosolic ribosome |  |
|  | GO:0030155 | 0.0357322 | Down | 2 | 121 | BP regulation of cell adhesion |  |
|  | GO:0030334 | 0.0297691 | Down | 2 | 120 | BP regulation of cell migration |  |
|  | GO:0030574 | 1.49E-06 | Down | 3 | 9 | BP collagen catabolic process |  |
|  | GO:0031102 | 0.03315 | Down | 1 | 14 | BP neuron projection regeneration |  |
|  | GO:0031103 | 0.0268639 | Down | 1 | 12 | BP axon regeneration |  |
|  | GO:0032365 | 0.0250169 | Down | 1 | 10 | BP intracellular lipid transport |  |
|  | GO:0032366 | 0.0223124 | Down | 1 | 9 | BP intracellular sterol transport |  |
|  | GO:0032367 | 0.0223124 | Down | 1 | 9 | BP intracellular cholesterol transport |  |
|  | GO:0032501 | 0.0075808 | Down | 8 | 1198 | BP multicellular organismal process |  |
|  | GO:0032642 | 0.0348175 | Down | 1 | 15 | BP regulation of chemokine production |  |
|  | GO:0032682 | 0.0232331 | Down | 1 | 10 | BP negative regulation of chemokine production |  |
|  | GO:0032934 | 0.0013497 | Down | 2 | 21 | MF sterol binding |  |
|  | GO:0032963 | 2.16E-06 | Down | 3 | 10 | BP collagen metabolic process |  |
|  | GO:0033344 | 0.0440891 | Down | 1 | 18 | BP cholesterol efflux |  |
|  | GO:0034442 | 0.0232331 | Down | 1 | 10 | BP regulation of lipoprotein oxidation |  |
|  | GO:0034443 | 0.0232331 | Down | 1 | 10 | BP negative regulation of lipoprotein oxidation |  |
|  | GO:0040012 | 0.0464191 | Down | 2 | 154 | BP regulation of locomotion |  |
|  | GO:0040013 | 0.0050548 | Down | 2 | 47 | BP negative regulation of locomotion |  |
|  | GO:0042246 | 0.0259277 | Down | 1 | 11 | BP tissue regeneration |  |
|  | GO:0042805 | 0.0341218 | Down | 1 | 11 | MF actinin binding |  |
|  | GO:0043178 | 0.0026457 | Down | 2 | 32 | MF alcohol binding |  |
|  | GO:0044236 | 2.16E-06 | Down | 3 | 10 | BP multicellular organism metabolic process |  |
|  | GO:0044238 | 0.0006275 | Down | 23 | 4888 | BP primary metabolic process |  |
|  | GO:0044243 | 1.49E-06 | Down | 3 | 9 | BP multicellular organism catabolic process |  |
|  | GO:0044259 | 2.16E-06 | Down | 3 | 10 | BP multicellular organismal macromolecule metabolic process |  |
|  | GO:0044421 | 2.98E-05 | Down | 10 | 821 | CC extracellular region part |  |
|  | GO:0044423 | 0.037505 | Down | 1 | 18 | CC virion part |  |
|  | GO:0044699 | 0.0053963 | Down | 22 | 5274 | BP single-organism process |  |
|  | GO:0044707 | 0.006756 | Down | 8 | 1173 | BP single-multicellular organism process |  |
|  | GO:0045833 | 0.0417699 | Down | 1 | 19 | BP negative regulation of lipid metabolic process |  |
|  | GO:0048589 | 0.0290325 | Down | 2 | 112 | BP developmental growth |  |
|  | GO:0048660 | 0.0456989 | Down | 1 | 19 | BP regulation of smooth muscle cell proliferation |  |
|  | GO:0048662 | 0.0284537 | Down | 1 | 12 | BP negative regulation of smooth muscle cell proliferation |  |
|  | GO:0048678 | 0.040408 | Down | 1 | 17 | BP response to axon injury |  |
|  | GO:0050746 | 0.0241537 | Down | 1 | 11 | BP regulation of lipoprotein metabolic process |  |
|  | GO:0050748 | 0.0232331 | Down | 1 | 10 | BP negative regulation of lipoprotein metabolic process |  |
|  | GO:0051270 | 0.0434378 | Down | 2 | 149 | BP regulation of cellular component movement |  |
|  | GO:0051271 | 0.0048124 | Down | 2 | 46 | BP negative regulation of cellular component movement |  |
|  | GO:0051371 | 0.0241812 | Down | 1 | 7 | MF muscle alpha-actinin binding |  |
|  | GO:0051393 | 0.0341218 | Down | 1 | 11 | MF alpha-actinin binding |  |
|  | GO:0051895 | 0.0347066 | Down | 1 | 13 | BP negative regulation of focal adhesion assembly |  |
|  | GO:0060587 | 0.0232331 | Down | 1 | 10 | BP regulation of lipoprotein lipid oxidation |  |
|  | GO:0060588 | 0.0232331 | Down | 1 | 10 | BP negative regulation of lipoprotein lipid oxidation |  |
|  | GO:0061564 | 0.0346962 | Down | 1 | 15 | BP axon development |  |
|  | GO:0070011 | 6.35E-08 | Down | 10 | 396 | MF peptidase activity, acting on L-amino acid peptides |  |
|  | GO:0071637 | 0.0232331 | Down | 1 | 10 | BP regulation of monocyte chemotactic protein-1 production |  |
|  | GO:0071638 | 0.0232331 | Down | 1 | 10 | BP negative regulation of monocyte chemotactic protein-1 production |  |
|  | GO:0071704 | 6.33E-05 | Down | 26 | 5089 | BP organic substance metabolic process |  |
|  | GO:1900015 | 0.0232331 | Down | 1 | 10 | BP regulation of cytokine production involved in inflammatory response | |
|  | GO:1900016 | 0.0232331 | Down | 1 | 10 | BP negative regulation of cytokine production involved in inflammatory response | |
|  | GO:1901071 | 0.000205 | Down | 3 | 49 | BP glucosamine-containing compound metabolic process |  |
|  | GO:1901135 | 0.0449335 | Down | 4 | 556 | BP carbohydrate derivative metabolic process |  |
|  | GO:1901889 | 0.0347066 | Down | 1 | 13 | BP negative regulation of cell junction assembly |  |
|  | GO:1903392 | 0.0347066 | Down | 1 | 13 | BP negative regulation of adherens junction organization |  |
|  | GO:2000097 | 0.0232331 | Down | 1 | 10 | BP regulation of smooth muscle cell-matrix adhesion |  |
|  | GO:2000098 | 0.0232331 | Down | 1 | 10 | BP negative regulation of smooth muscle cell-matrix adhesion |  |
|  | GO:2000145 | 0.0321806 | Down | 2 | 125 | BP regulation of cell motility |  |
|  | GO:2000401 | 0.0266748 | Down | 1 | 12 | BP regulation of lymphocyte migration |  |
|  | GO:2000402 | 0.0232331 | Down | 1 | 10 | BP negative regulation of lymphocyte migration |  |
|  | GO:2000404 | 0.0253399 | Down | 1 | 11 | BP regulation of T cell migration |  |
|  | GO:2000405 | 0.0232331 | Down | 1 | 10 | BP negative regulation of T cell migration |  |
| Control vs. Cold | |  |  |  |  |  |  |
|  | GO:0001503 | 0.0035935 | Up | 1 | 10 | BP ossification |  |
|  | GO:0001525 | 0.012852 | Up | 1 | 40 | BP angiogenesis |  |
|  | GO:0001541 | 0.0029069 | Up | 1 | 6 | BP ovarian follicle development |  |
|  | GO:0001558 | 0.0186287 | Up | 1 | 63 | BP regulation of cell growth |  |
|  | GO:0001666 | 0.0147013 | Up | 1 | 51 | BP response to hypoxia |  |
|  | GO:0001763 | 0.0108628 | Up | 1 | 31 | BP morphogenesis of a branching structure |  |
|  | GO:0001935 | 0.0041966 | Up | 1 | 10 | BP endothelial cell proliferation |  |
|  | GO:0001952 | 0.009823 | Up | 1 | 27 | BP regulation of cell-matrix adhesion |  |
|  | GO:0001953 | 0.0043363 | Up | 1 | 14 | BP negative regulation of cell-matrix adhesion |  |
|  | GO:0001958 | 0.0019328 | Up | 1 | 5 | BP endochondral ossification |  |
|  | GO:0001990 | 0.0013205 | Up | 1 | 3 | BP regulation of systemic arterial blood pressure by hormone |  |
|  | GO:0001998 | 0.0003145 | Up | 1 | 1 | BP angiotensin mediated vasoconstriction involved in regulation of systemic arterial blood pressure | |
|  | GO:0002009 | 0.039098 | Up | 1 | 117 | BP morphogenesis of an epithelium |  |
|  | GO:0003013 | 0.0090432 | Up | 1 | 22 | BP circulatory system process |  |
|  | GO:0003014 | 0.0020559 | Up | 1 | 7 | BP renal system process |  |
|  | GO:0003018 | 0.0027318 | Up | 1 | 10 | BP vascular process in circulatory system |  |
|  | GO:0003044 | 0.0013205 | Up | 1 | 3 | BP regulation of systemic arterial blood pressure mediated by a chemical signal | |
|  | GO:0003073 | 0.0015955 | Up | 1 | 4 | BP regulation of systemic arterial blood pressure |  |
|  | GO:0003084 | 0.0005402 | Up | 1 | 2 | BP positive regulation of systemic arterial blood pressure |  |
|  | GO:0004222 | 0.0136968 | Up | 1 | 42 | MF metalloendopeptidase activity |  |
|  | GO:0004344 | 0.0023929 | Up | 1 | 8 | MF glucose dehydrogenase activity |  |
|  | GO:0004497 | 0.0269942 | Up | 1 | 104 | MF monooxygenase activity |  |
|  | GO:0005102 | 0.0386201 | Up | 1 | 130 | MF receptor binding |  |
|  | GO:0005178 | 0.0043154 | Up | 1 | 14 | MF integrin binding |  |
|  | GO:0005506 | 0.0417691 | Up | 1 | 151 | MF iron ion binding |  |
|  | GO:0005575 | 0.032795 | Up | 5 | 6749 | CC cellular_component |  |
|  | GO:0005576 | 0.0001696 | Up | 3 | 375 | CC extracellular region |  |
|  | GO:0005996 | 0.023304 | Up | 1 | 82 | BP monosaccharide metabolic process |  |
|  | GO:0006006 | 0.0126383 | Up | 1 | 43 | BP glucose metabolic process |  |
|  | GO:0006066 | 0.0338841 | Up | 1 | 112 | BP alcohol metabolic process |  |
|  | GO:0006950 | 0.0199822 | Up | 2 | 742 | **BP response to stress** |  |
|  | GO:0006979 | 0.0235305 | Up | 1 | 83 | **BP response to oxidative stress** |  |
|  | GO:0007162 | 0.0116352 | Up | 1 | 39 | BP negative regulation of cell adhesion |  |
|  | GO:0007610 | 0.0022644 | Up | 2 | 240 | BP behavior |  |
|  | GO:0007617 | 0.0073453 | Up | 1 | 27 | BP mating behavior |  |
|  | GO:0007622 | 0.0098379 | Up | 1 | 31 | BP rhythmic behavior |  |
|  | GO:0007623 | 0.0179996 | Up | 1 | 59 | BP circadian rhythm |  |
|  | GO:0007631 | 0.0076373 | Up | 1 | 28 | BP feeding behavior |  |
|  | GO:0008047 | 0.0314272 | Up | 1 | 100 | MF enzyme activator activity |  |
|  | GO:0008049 | 0.0083797 | Up | 1 | 29 | BP male courtship behavior |  |
|  | GO:0008150 | 0.0289351 | Up | 5 | 6506 | BP biological_process |  |
|  | GO:0008217 | 0.003388 | Up | 1 | 11 | BP regulation of blood pressure |  |
|  | GO:0008237 | 0.0279958 | Up | 1 | 90 | MF metallopeptidase activity |  |
|  | GO:0008283 | 0.0427453 | Up | 1 | 139 | BP cell proliferation |  |
|  | GO:0008343 | 0.0039237 | Up | 1 | 15 | BP adult feeding behavior |  |
|  | GO:0008347 | 0.0063677 | Up | 1 | 21 | BP glial cell migration |  |
|  | GO:0008364 | 0.0023929 | Up | 1 | 8 | BP pupal chitin-based cuticle development |  |
|  | GO:0008406 | 0.009211 | Up | 1 | 30 | BP gonad development |  |
|  | GO:0008584 | 0.0021754 | Up | 1 | 6 | BP male gonad development |  |
|  | GO:0008812 | 0.0048967 | Up | 1 | 17 | MF choline dehydrogenase activity |  |
|  | GO:0009605 | 0.0035535 | Up | 2 | 301 | BP response to external stimulus |  |
|  | GO:0009612 | 0.0090363 | Up | 1 | 28 | BP response to mechanical stimulus |  |
|  | GO:0009719 | 0.0468474 | Up | 1 | 153 | BP response to endogenous stimulus |  |
|  | GO:0009725 | 0.0319491 | Up | 1 | 100 | BP response to hormone |  |
|  | GO:0009991 | 0.0236865 | Up | 1 | 83 | BP response to extracellular stimulus |  |
|  | GO:0010810 | 0.0146258 | Up | 1 | 42 | BP regulation of cell-substrate adhesion |  |
|  | GO:0010812 | 0.0061463 | Up | 1 | 19 | BP negative regulation of cell-substrate adhesion |  |
|  | GO:0010952 | 0.0060458 | Up | 1 | 17 | BP positive regulation of peptidase activity |  |
|  | GO:0014070 | 0.029816 | Up | 1 | 100 | BP response to organic cyclic compound |  |
|  | GO:0016485 | 0.0220219 | Up | 1 | 71 | BP protein processing |  |
|  | GO:0016491 | 0.0095439 | Up | 2 | 519 | **MF oxidoreductase activity** |  |
|  | GO:0016504 | 0.0033845 | Up | 1 | 9 | MF peptidase activator activity |  |
|  | GO:0016614 | 0.0325545 | Up | 1 | 108 | MF oxidoreductase activity, acting on CH-OH group of donors |  |
|  | GO:0016705 | 0.0370371 | Up | 1 | 138 | MF oxidoreductase activity, acting on paired donors, with incorporation or reduction of molecular oxygen | |
|  | GO:0016712 | 0.0034897 | Up | 1 | 13 | MF oxidoreductase activity, acting on paired donors, with incorporation or reduction of molecular oxygen, reduced flavin or flavoprotein as one donor, and incorporation of one atom of oxygen | |
|  | GO:0019098 | 0.0001035 | Up | 2 | 52 | BP reproductive behavior |  |
|  | GO:0019318 | 0.0193341 | Up | 1 | 68 | BP hexose metabolic process |  |
|  | GO:0020037 | 0.034937 | Up | 1 | 130 | MF heme binding |  |
|  | GO:0022414 | 6.32E-06 | Up | 4 | 427 | BP reproductive process |  |
|  | GO:0022602 | 0.0033555 | Up | 1 | 8 | BP ovulation cycle process |  |
|  | GO:0030155 | 0.0307776 | Up | 1 | 97 | BP regulation of cell adhesion |  |
|  | GO:0030162 | 0.0419496 | Up | 1 | 138 | **BP regulation of proteolysis** |  |
|  | GO:0030307 | 0.0076994 | Up | 1 | 25 | BP positive regulation of cell growth |  |
|  | GO:0030324 | 0.0030185 | Up | 1 | 9 | BP lung development |  |
|  | GO:0030334 | 0.0280819 | Up | 1 | 95 | BP regulation of cell migration |  |
|  | GO:0030335 | 0.0113778 | Up | 1 | 39 | BP positive regulation of cell migration |  |
|  | GO:0030534 | 0.0236674 | Up | 1 | 80 | BP adult behavior |  |
|  | GO:0030574 | 0.0023423 | Up | 1 | 7 | BP collagen catabolic process |  |
|  | GO:0031012 | 0.017927 | Up | 1 | 56 | CC extracellular matrix |  |
|  | GO:0031638 | 0.0069523 | Up | 1 | 21 | BP zymogen activation |  |
|  | GO:0031667 | 0.0228583 | Up | 1 | 80 | BP response to nutrient levels |  |
|  | GO:0032368 | 0.0046544 | Up | 1 | 15 | BP regulation of lipid transport |  |
|  | GO:0032370 | 0.0030457 | Up | 1 | 9 | BP positive regulation of lipid transport |  |
|  | GO:0032403 | 0.0458857 | Up | 1 | 147 | MF protein complex binding |  |
|  | GO:0032501 | 0.000128 | Up | 4 | 922 | BP multicellular organismal process |  |
|  | GO:0032879 | 0.0059917 | Up | 2 | 386 | BP regulation of localization |  |
|  | GO:0032963 | 0.0028824 | Up | 1 | 9 | BP collagen metabolic process |  |
|  | GO:0033057 | 0.0122008 | Up | 1 | 40 | none |  |
|  | GO:0033993 | 0.031318 | Up | 1 | 98 | BP response to lipid |  |
|  | GO:0035150 | 0.0077351 | Up | 1 | 21 | BP regulation of tube size |  |
|  | GO:0035239 | 0.0322705 | Up | 1 | 99 | BP tube morphogenesis |  |
|  | GO:0035988 | 0.0010724 | Up | 1 | 2 | BP chondrocyte proliferation |  |
|  | GO:0036075 | 0.0019328 | Up | 1 | 5 | BP replacement ossification |  |
|  | GO:0036293 | 0.0149852 | Up | 1 | 52 | BP response to decreased oxygen levels |  |
|  | GO:0040003 | 0.0082947 | Up | 1 | 22 | BP chitin-based cuticle development |  |
|  | GO:0040008 | 0.0484987 | Up | 1 | 163 | BP regulation of growth |  |
|  | GO:0040012 | 0.0359963 | Up | 1 | 123 | BP regulation of locomotion |  |
|  | GO:0040017 | 0.0157047 | Up | 1 | 54 | BP positive regulation of locomotion |  |
|  | GO:0042310 | 0.0007874 | Up | 1 | 3 | BP vasoconstriction |  |
|  | GO:0042335 | 0.0096121 | Up | 1 | 27 | BP cuticle development |  |
|  | GO:0042337 | 0.0026568 | Up | 1 | 9 | BP cuticle development involved in chitin-based cuticle molting cycle |  |
|  | GO:0042470 | 0.0127397 | Up | 1 | 37 | CC melanosome |  |
|  | GO:0042594 | 0.015095 | Up | 1 | 54 | BP response to starvation |  |
|  | GO:0042595 | 0.0036807 | Up | 1 | 14 | BP behavioral response to starvation |  |
|  | GO:0043615 | 0.0013226 | Up | 1 | 3 | BP astrocyte cell migration |  |
|  | GO:0043627 | 0.0081509 | Up | 1 | 26 | BP response to estrogen |  |
|  | GO:0044057 | 0.0176479 | Up | 1 | 52 | BP regulation of system process |  |
|  | GO:0044060 | 0.001318 | Up | 1 | 5 | BP regulation of endocrine process |  |
|  | GO:0044236 | 0.0028824 | Up | 1 | 9 | BP multicellular organism metabolic process |  |
|  | GO:0044243 | 0.0023423 | Up | 1 | 7 | BP multicellular organism catabolic process |  |
|  | GO:0044259 | 0.0028824 | Up | 1 | 9 | BP multicellular organismal macromolecule metabolic process |  |
|  | GO:0044699 | 0.0308432 | Up | 4 | 4262 | BP single-organism process |  |
|  | GO:0044703 | 0.000139 | Up | 2 | 59 | BP multi-organism reproductive process |  |
|  | GO:0044705 | 0.0128033 | Up | 1 | 42 | BP multi-organism reproductive behavior |  |
|  | GO:0044706 | 0.0001219 | Up | 2 | 55 | BP multi-multicellular organism process |  |
|  | GO:0044707 | 0.002351 | Up | 3 | 901 | BP single-multicellular organism process |  |
|  | GO:0044710 | 0.0185697 | Up | 3 | 2002 | BP single-organism metabolic process |  |
|  | GO:0045297 | 0.0024977 | Up | 1 | 10 | BP post-mating behavior |  |
|  | GO:0045777 | 0.0005402 | Up | 1 | 2 | BP positive regulation of blood pressure |  |
|  | GO:0045862 | 0.0187423 | Up | 1 | 61 | **BP positive regulation of proteolysis** |  |
|  | GO:0045927 | 0.0171289 | Up | 1 | 54 | BP positive regulation of growth |  |
|  | GO:0046692 | 0.0023929 | Up | 1 | 8 | BP sperm competition |  |
|  | GO:0046693 | 0.0023929 | Up | 1 | 8 | BP sperm storage |  |
|  | GO:0046883 | 0.0089167 | Up | 1 | 29 | BP regulation of hormone secretion |  |
|  | GO:0046887 | 0.0030875 | Up | 1 | 11 | BP positive regulation of hormone secretion |  |
|  | GO:0046903 | 0.0293746 | Up | 1 | 93 | BP secretion |  |
|  | GO:0046906 | 0.0353638 | Up | 1 | 131 | MF tetrapyrrole binding |  |
|  | GO:0046914 | 0.0212133 | Up | 2 | 774 | MF transition metal ion binding |  |
|  | GO:0048511 | 0.0002949 | Up | 2 | 83 | BP rhythmic process |  |
|  | GO:0048518 | 0.0404043 | Up | 2 | 1062 | BP positive regulation of biological process |  |
|  | GO:0048522 | 0.029625 | Up | 2 | 899 | BP positive regulation of cellular process |  |
|  | GO:0048545 | 0.0156255 | Up | 1 | 51 | BP response to steroid hormone |  |
|  | GO:0048562 | 0.0067584 | Up | 1 | 20 | BP embryonic organ morphogenesis |  |
|  | GO:0048598 | 0.0376299 | Up | 1 | 116 | BP embryonic morphogenesis |  |
|  | GO:0048608 | 0.0186292 | Up | 1 | 57 | BP reproductive structure development |  |
|  | GO:0048609 | 0.0019874 | Up | 2 | 224 | BP multicellular organismal reproductive process |  |
|  | GO:0048701 | 0.0024084 | Up | 1 | 5 | BP embryonic cranial skeleton morphogenesis |  |
|  | GO:0048704 | 0.0048484 | Up | 1 | 13 | BP embryonic skeletal system morphogenesis |  |
|  | GO:0048705 | 0.0061038 | Up | 1 | 18 | BP skeletal system morphogenesis |  |
|  | GO:0048729 | 0.0429419 | Up | 1 | 127 | BP tissue morphogenesis |  |
|  | GO:0048754 | 0.0091914 | Up | 1 | 27 | BP branching morphogenesis of an epithelial tube |  |
|  | GO:0048770 | 0.0127397 | Up | 1 | 37 | CC pigment granule |  |
|  | GO:0048771 | 0.0025501 | Up | 1 | 8 | BP tissue remodeling |  |
|  | GO:0048856 | 0.0290652 | Up | 2 | 855 | BP anatomical structure development |  |
|  | GO:0050660 | 0.0193138 | Up | 1 | 63 | MF flavin adenine dinucleotide binding |  |
|  | GO:0050662 | 0.0440835 | Up | 1 | 151 | MF coenzyme binding |  |
|  | GO:0050673 | 0.0067604 | Up | 1 | 20 | BP epithelial cell proliferation |  |
|  | GO:0050839 | 0.0081155 | Up | 1 | 28 | MF cell adhesion molecule binding |  |
|  | GO:0050880 | 0.0021579 | Up | 1 | 8 | BP regulation of blood vessel size |  |
|  | GO:0050886 | 0.0013205 | Up | 1 | 3 | BP endocrine process |  |
|  | GO:0050896 | 0.0435624 | Up | 2 | 1111 | BP response to stimulus |  |
|  | GO:0051046 | 0.0238822 | Up | 1 | 80 | BP regulation of secretion |  |
|  | GO:0051047 | 0.0107478 | Up | 1 | 37 | BP positive regulation of secretion |  |
|  | GO:0051050 | 0.0335962 | Up | 1 | 111 | BP positive regulation of transport |  |
|  | GO:0051129 | 0.0465222 | Up | 1 | 147 | BP negative regulation of cellular component organization |  |
|  | GO:0051270 | 0.0335422 | Up | 1 | 113 | BP regulation of cellular component movement |  |
|  | GO:0051272 | 0.0129708 | Up | 1 | 44 | BP positive regulation of cellular component movement |  |
|  | GO:0051345 | 0.0366179 | Up | 1 | 115 | BP positive regulation of hydrolase activity |  |
|  | GO:0051704 | 0.0044473 | Up | 2 | 329 | BP multi-organism process |  |
|  | GO:0051705 | 0.0199758 | Up | 1 | 66 | BP multi-organism behavior |  |
|  | GO:0051893 | 0.007877 | Up | 1 | 22 | BP regulation of focal adhesion assembly |  |
|  | GO:0051895 | 0.0038666 | Up | 1 | 12 | BP negative regulation of focal adhesion assembly |  |
|  | GO:0052547 | 0.0196365 | Up | 1 | 63 | BP regulation of peptidase activity |  |
|  | GO:0055114 | 0.0062261 | Up | 2 | 404 | BP oxidation-reduction process |  |
|  | GO:0060179 | 0.0094947 | Up | 1 | 32 | BP male mating behavior |  |
|  | GO:0061134 | 0.0194687 | Up | 1 | 60 | MF peptidase regulator activity |  |
|  | GO:0061138 | 0.0095014 | Up | 1 | 28 | BP morphogenesis of a branching epithelium |  |
|  | GO:0070330 | 0.0034897 | Up | 1 | 13 | MF aromatase activity |  |
|  | GO:0070482 | 0.0153647 | Up | 1 | 53 | BP response to oxygen levels |  |
|  | GO:0090066 | 0.0350658 | Up | 1 | 98 | BP regulation of anatomical structure size |  |
|  | GO:0090109 | 0.007877 | Up | 1 | 22 | BP regulation of cell-substrate junction assembly |  |
|  | GO:0097094 | 0.0010894 | Up | 1 | 2 | BP craniofacial suture morphogenesis |  |
|  | GO:0097254 | 0.0003145 | Up | 1 | 1 | BP renal tubular secretion |  |
|  | GO:1901615 | 0.0457483 | Up | 1 | 152 | BP organic hydroxy compound metabolic process |  |
|  | GO:1901888 | 0.007877 | Up | 1 | 22 | BP regulation of cell junction assembly |  |
|  | GO:1901889 | 0.0038666 | Up | 1 | 12 | BP negative regulation of cell junction assembly |  |
|  | GO:1903391 | 0.007877 | Up | 1 | 22 | BP regulation of adherens junction organization |  |
|  | GO:1903392 | 0.0038666 | Up | 1 | 12 | BP negative regulation of adherens junction organization |  |
|  | GO:1903530 | 0.0195732 | Up | 1 | 66 | BP regulation of secretion by cell |  |
|  | GO:1903532 | 0.008892 | Up | 1 | 30 | BP positive regulation of secretion by cell |  |
|  | GO:2000145 | 0.0288724 | Up | 1 | 98 | BP regulation of cell motility |  |
|  | GO:2000147 | 0.0116508 | Up | 1 | 40 | BP positive regulation of cell motility |  |
|  | GO:2000831 | 0.001318 | Up | 1 | 5 | BP regulation of steroid hormone secretion |  |
|  | GO:2000833 | 0.0008027 | Up | 1 | 3 | BP positive regulation of steroid hormone secretion |  |
|  | GO:2000861 | 0.0003145 | Up | 1 | 1 | BP regulation of estrogen secretion |  |
|  | GO:2000863 | 0.0003145 | Up | 1 | 1 | BP positive regulation of estrogen secretion |  |
|  | GO:0003824 | 0.0033608 | Down | 9 | 3325 | MF catalytic activity |  |
|  | GO:0004175 | 6.45E-08 | Down | 6 | 208 | MF endopeptidase activity |  |
|  | GO:0004252 | 3.75E-07 | Down | 4 | 65 | MF serine-type endopeptidase activity |  |
|  | GO:0005509 | 0.0179423 | Down | 2 | 199 | MF calcium ion binding |  |
|  | GO:0005615 | 3.35E-05 | Down | 4 | 189 | CC extracellular space |  |
|  | GO:0006030 | 0.0309177 | Down | 1 | 32 | BP chitin metabolic process |  |
|  | GO:0006040 | 0.0409009 | Down | 1 | 44 | BP amino sugar metabolic process |  |
|  | GO:0006508 | 1.18E-05 | Down | 5 | 288 | **BP proteolysis** |  |
|  | GO:0007586 | 0.0089272 | Down | 1 | 10 | BP digestion |  |
|  | GO:0008061 | 0.0242959 | Down | 1 | 25 | MF chitin binding |  |
|  | GO:0008152 | 0.006599 | Down | 10 | 4466 | BP metabolic process |  |
|  | GO:0008233 | 9.41E-07 | Down | 6 | 328 | MF peptidase activity |  |
|  | GO:0008236 | 8.49E-07 | Down | 4 | 78 | MF serine-type peptidase activity |  |
|  | GO:0009056 | 0.0065046 | Down | 4 | 747 | BP catabolic process |  |
|  | GO:0016787 | 0.0003105 | Down | 7 | 1368 | MF hydrolase activity |  |
|  | GO:0017171 | 1.04E-06 | Down | 4 | 82 | MF serine hydrolase activity |  |
|  | GO:0019538 | 0.0085875 | Down | 5 | 1290 | BP protein metabolic process |  |
|  | GO:0043170 | 0.0286532 | Down | 7 | 3211 | BP macromolecule metabolic process |  |
|  | GO:0044238 | 0.028252 | Down | 8 | 3933 | BP primary metabolic process |  |
|  | GO:0044421 | 0.0045619 | Down | 4 | 686 | CC extracellular region part |  |
|  | GO:0044712 | 0.000827 | Down | 4 | 424 | BP single-organism catabolic process |  |
|  | GO:0044877 | 0.0393891 | Down | 2 | 310 | MF macromolecular complex binding |  |
|  | GO:0070011 | 7.46E-07 | Down | 6 | 312 | MF peptidase activity, acting on L-amino acid peptides |  |
|  | GO:0071704 | 0.0120396 | Down | 9 | 4098 | BP organic substance metabolic process |  |
|  | GO:1901071 | 0.036141 | Down | 1 | 38 | BP glucosamine-containing compound metabolic process |  |
|  |  |  |  |  |  |  |  |
| *Grylloblatta gurneyi* Mt. Shasta | | |  |  |  |  |  |
| Control vs Heat | |  |  |  |  |  |  |
|  | GO:0000166 | 0.0479844 | Up | 2 | 1378 | MF nucleotide binding |  |
|  | GO:0003674 | 0.0082858 | Down | 20 | 7380 | MF molecular_function |  |
|  | GO:0003824 | 0.0006329 | Down | 15 | 3704 | MF catalytic activity |  |
|  | GO:0004175 | 1.45E-07 | Down | 7 | 245 | MF endopeptidase activity |  |
|  | GO:0004180 | 0.0005061 | Down | 2 | 18 | MF carboxypeptidase activity |  |
|  | GO:0004181 | 0.0001183 | Down | 2 | 10 | MF metallocarboxypeptidase activity |  |
|  | GO:0004252 | 6.10E-11 | Down | 7 | 83 | MF serine-type endopeptidase activity |  |
|  | GO:0004344 | 2.06E-06 | Up | 2 | 9 | MF glucose dehydrogenase activity |  |
|  | GO:0004558 | 0.0175862 | Down | 1 | 9 | MF alpha-1,4-glucosidase activity |  |
|  | GO:0005496 | 0.037841 | Down | 1 | 25 | MF steroid binding |  |
|  | GO:0005575 | 0.0080202 | Down | 20 | 7334 | CC cellular_component |  |
|  | GO:0005576 | 0.004811 | Up | 2 | 416 | CC extracellular region |  |
|  | GO:0005615 | 2.00E-06 | Down | 6 | 209 | CC extracellular space |  |
|  | GO:0005975 | 0.0037403 | Up | 2 | 356 | BP carbohydrate metabolic process |  |
|  | GO:0005996 | 0.0002158 | Up | 2 | 83 | BP monosaccharide metabolic process |  |
|  | GO:0006006 | 6.22E-05 | Up | 2 | 45 | BP glucose metabolic process |  |
|  | GO:0006022 | 0.0002675 | Down | 3 | 73 | BP aminoglycan metabolic process |  |
|  | GO:0006030 | 4.18E-05 | Down | 3 | 37 | BP chitin metabolic process |  |
|  | GO:0006040 | 8.75E-05 | Down | 3 | 53 | BP amino sugar metabolic process |  |
|  | GO:0006066 | 0.0004564 | Up | 2 | 122 | BP alcohol metabolic process |  |
|  | GO:0006508 | 7.25E-09 | Down | 9 | 341 | BP proteolysis |  |
|  | GO:0006629 | 0.0053827 | Down | 4 | 418 | BP lipid metabolic process |  |
|  | GO:0007586 | 0.0003361 | Down | 2 | 18 | BP digestion |  |
|  | GO:0008061 | 2.17E-05 | Down | 3 | 32 | MF chitin binding |  |
|  | GO:0008150 | 0.0125109 | Down | 19 | 7079 | BP biological_process |  |
|  | GO:0008152 | 0.0001308 | Down | 19 | 4865 | BP metabolic process |  |
|  | GO:0008233 | 1.59E-08 | Down | 9 | 393 | MF peptidase activity |  |
|  | GO:0008235 | 0.0006793 | Down | 2 | 24 | MF metalloexopeptidase activity |  |
|  | GO:0008236 | 2.66E-10 | Down | 7 | 102 | MF serine-type peptidase activity |  |
|  | GO:0008237 | 0.0134676 | Down | 2 | 104 | MF metallopeptidase activity |  |
|  | GO:0008238 | 0.0075989 | Down | 2 | 83 | MF exopeptidase activity |  |
|  | GO:0008364 | 2.06E-06 | Up | 2 | 9 | BP pupal chitin-based cuticle development |  |
|  | GO:0008374 | 0.0045707 | Up | 1 | 18 | MF O-acyltransferase activity |  |
|  | GO:0008812 | 1.15E-05 | Up | 2 | 21 | MF choline dehydrogenase activity |  |
|  | GO:0009056 | 0.0004989 | Down | 7 | 845 | BP catabolic process |  |
|  | GO:0015485 | 0.0281636 | Down | 1 | 18 | MF cholesterol binding |  |
|  | GO:0015850 | 0.0489087 | Down | 1 | 34 | BP organic hydroxy compound transport |  |
|  | GO:0015918 | 0.0393148 | Down | 1 | 27 | BP sterol transport |  |
|  | GO:0015926 | 0.0265804 | Down | 1 | 14 | MF glucosidase activity |  |
|  | GO:0016042 | 0.0249036 | Up | 1 | 95 | BP lipid catabolic process |  |
|  | GO:0016491 | 0.0099113 | Up | 2 | 603 | MF oxidoreductase activity |  |
|  | GO:0016614 | 0.0004474 | Up | 2 | 118 | MF oxidoreductase activity, acting on CH-OH group of donors |  |
|  | GO:0016746 | 0.03343 | Up | 1 | 131 | MF transferase activity, transferring acyl groups |  |
|  | GO:0016747 | 0.027827 | Up | 1 | 109 | MF transferase activity, transferring acyl groups other than amino-acyl groups | |
|  | GO:0016787 | 7.90E-07 | Down | 13 | 1530 | MF hydrolase activity |  |
|  | GO:0016788 | 0.0463052 | Down | 3 | 501 | MF hydrolase activity, acting on ester bonds |  |
|  | GO:0017171 | 3.49E-10 | Down | 7 | 105 | MF serine hydrolase activity |  |
|  | GO:0019028 | 2.68E-05 | Down | 2 | 6 | CC viral capsid |  |
|  | GO:0019318 | 0.0001447 | Up | 2 | 68 | BP hexose metabolic process |  |
|  | GO:0019538 | 0.0004853 | Down | 9 | 1399 | BP protein metabolic process |  |
|  | GO:0022414 | 0.0066885 | Up | 2 | 495 | BP reproductive process |  |
|  | GO:0030301 | 0.0351475 | Down | 1 | 24 | BP cholesterol transport |  |
|  | GO:0030574 | 1.96E-09 | Down | 4 | 9 | BP collagen catabolic process |  |
|  | GO:0032365 | 0.0142943 | Down | 1 | 10 | BP intracellular lipid transport |  |
|  | GO:0032366 | 0.0102307 | Down | 1 | 7 | BP intracellular sterol transport |  |
|  | GO:0032367 | 0.0102307 | Down | 1 | 7 | BP intracellular cholesterol transport |  |
|  | GO:0032450 | 0.0152742 | Down | 1 | 8 | MF maltose alpha-glucosidase activity |  |
|  | GO:0032501 | 0.0271216 | Up | 2 | 1027 | BP multicellular organismal process |  |
|  | GO:0032934 | 0.0281636 | Down | 1 | 18 | MF sterol binding |  |
|  | GO:0032963 | 5.20E-09 | Down | 4 | 11 | BP collagen metabolic process |  |
|  | GO:0033344 | 0.0260483 | Down | 1 | 17 | BP cholesterol efflux |  |
|  | GO:0040003 | 1.96E-05 | Up | 2 | 27 | BP chitin-based cuticle development |  |
|  | GO:0042335 | 2.71E-05 | Up | 2 | 32 | BP cuticle development |  |
|  | GO:0042337 | 3.04E-06 | Up | 2 | 11 | BP cuticle development involved in chitin-based cuticle molting cycle |  |
|  | GO:0042632 | 0.0293339 | Down | 1 | 18 | BP cholesterol homeostasis |  |
|  | GO:0043168 | 0.0464504 | Up | 2 | 1355 | MF anion binding |  |
|  | GO:0043170 | 0.0079888 | Down | 12 | 3475 | BP macromolecule metabolic process |  |
|  | GO:0043178 | 0.0384067 | Down | 1 | 28 | MF alcohol binding |  |
|  | GO:0044236 | 5.20E-09 | Down | 4 | 11 | BP multicellular organism metabolic process |  |
|  | GO:0044238 | 0.0008883 | Down | 16 | 4282 | BP primary metabolic process |  |
|  | GO:0044243 | 1.96E-09 | Down | 4 | 9 | BP multicellular organism catabolic process |  |
|  | GO:0044259 | 5.20E-09 | Down | 4 | 11 | BP multicellular organismal macromolecule metabolic process |  |
|  | GO:0044281 | 0.0280695 | Up | 2 | 1030 | BP small molecule metabolic process |  |
|  | GO:0044421 | 0.0002448 | Down | 7 | 730 | CC extracellular region part |  |
|  | GO:0044423 | 8.57E-05 | Down | 2 | 9 | CC virion part |  |
|  | GO:0044703 | 0.0001285 | Up | 2 | 68 | BP multi-organism reproductive process |  |
|  | GO:0044706 | 0.0001125 | Up | 2 | 64 | BP multi-multicellular organism process |  |
|  | GO:0044707 | 0.0050489 | Down | 6 | 1001 | BP single-multicellular organism process |  |
|  | GO:0044710 | 0.0159494 | Up | 3 | 2176 | BP single-organism metabolic process |  |
|  | GO:0044712 | 1.52E-05 | Down | 7 | 475 | BP single-organism catabolic process |  |
|  | GO:0044723 | 0.0021123 | Up | 2 | 267 | BP single-organism carbohydrate metabolic process |  |
|  | GO:0046692 | 2.06E-06 | Up | 2 | 9 | BP sperm competition |  |
|  | GO:0046693 | 2.06E-06 | Up | 2 | 9 | BP sperm storage |  |
|  | GO:0048037 | 0.0016864 | Up | 2 | 241 | MF cofactor binding |  |
|  | GO:0048609 | 0.001932 | Up | 2 | 264 | BP multicellular organismal reproductive process |  |
|  | GO:0048856 | 0.0225634 | Up | 2 | 929 | BP anatomical structure development |  |
|  | GO:0050660 | 0.0001682 | Up | 2 | 76 | MF flavin adenine dinucleotide binding |  |
|  | GO:0050662 | 0.0009034 | Up | 2 | 176 | MF coenzyme binding |  |
|  | GO:0051704 | 0.0033779 | Up | 2 | 344 | BP multi-organism process |  |
|  | GO:0055088 | 0.0450416 | Down | 1 | 30 | BP lipid homeostasis |  |
|  | GO:0055092 | 0.0338036 | Down | 1 | 21 | BP sterol homeostasis |  |
|  | GO:0070011 | 1.12E-08 | Down | 9 | 378 | MF peptidase activity, acting on L-amino acid peptides |  |
|  | GO:0071704 | 4.22E-05 | Down | 19 | 4490 | BP organic substance metabolic process |  |
|  | GO:0090599 | 0.021485 | Down | 1 | 11 | MF alpha-glucosidase activity |  |
|  | GO:1901071 | 6.94E-05 | Down | 3 | 49 | BP glucosamine-containing compound metabolic process |  |
|  | GO:1901265 | 0.0479844 | Up | 2 | 1378 | MF nucleoside phosphate binding |  |
|  | GO:1901615 | 0.0008753 | Up | 2 | 170 | BP organic hydroxy compound metabolic process |  |
| Control vs. Cold | |  |  |  |  |  |  |
|  | Not applicable | |  |  |  |  |  |
|  |  |  |  |  |  |  |  |
| *Grylloblatta gurneyi* Lava Beds NM | | |  |  |  |  |  |
| Control vs Heat | |  |  |  |  |  |  |
|  | Nothiing |  |  |  |  |  |  |
| Control vs. Cold | |  |  |  |  |  |  |
|  | Not applicable | |  |  |  |  |  |

**Supplementary Table S14.** Mean expression of key heat shock proteins in experimental treatments of Mantophasmatodea and Grylloblattodea. Expression values are based on trimmed mean of M (TMM), a normalized measure of expression within each species.

| **Annotation** | **Transcript ID** | **Control TMM** | **Cold TMM** | **Heat TMM** |
| --- | --- | --- | --- | --- |
| ***Karoophasma biedouwense*** | | | | |
| Alpha-crystallin A chain (hsp) | DN139921_c2_g10 | 17.30 | 31.53 | 325.94 |
| Heat shock 70 kDa protein cognate 1 | DN131494_c0_g5 | 0.03 | 0.00 | 0.29 |
| Heat shock 70 kDa protein cognate 1 | DN153399_c0_g1 | 0.00 | 0.10 | 0.00 |
| Heat shock 70 kDa protein cognate 2 | DN122737_c0_g1 | 18.20 | 28.10 | 66.92 |
| Heat shock 70 kDa protein cognate 3 | DN134699_c0_g2 | 9.38 | 12.43 | 27.89 |
| Heat shock 70 kDa protein cognate 3 | DN156706_c0_g1 | 0.00 | 0.10 | 0.00 |
| Heat shock 70 kDa protein cognate 3 | DN18183_c0_g1 | 0.09 | 0.02 | 0.00 |
| Heat shock 70 kDa protein cognate 3 | DN219317_c0_g1 | 0.00 | 0.00 | 0.04 |
| Heat shock 70 kDa protein cognate 3 | DN36788_c0_g1 | 0.00 | 0.04 | 0.00 |
| Heat shock 70 kDa protein cognate 4 | DN119835_c0_g1 | 0.45 | 0.31 | 0.11 |
| Heat shock 70 kDa protein cognate 4 | DN121968_c0_g1 | 0.00 | 0.44 | 0.00 |
| Heat shock 70 kDa protein cognate 4 | DN121968_c0_g1 | 0.00 | 0.44 | 0.00 |
| Heat shock 70 kDa protein cognate 4 | DN136142_c0_g1 | 614.59 | 911.83 | 1315.51 |
| Heat shock 70 kDa protein cognate 4 | DN137035_c0_g11 | 0.77 | 2.36 | 3.98 |
| Heat shock 70 kDa protein cognate 4 | DN211102_c0_g1 | 0.10 | 0.00 | 0.01 |
| Heat shock 70 kDa protein cognate 4 | DN68858_c0_g1 | 0.17 | 0.00 | 0.01 |
| Heat shock 70 kDa protein cognate 5 | DN138720_c3_g2 | 23.57 | 38.46 | 49.65 |
| Heat shock 70 kDa protein cognate 5 | DN142320_c0_g1 | 0.00 | 0.00 | 0.02 |
| Heat shock 70 kDa protein cognate 5 | DN157641_c0_g1 | 0.00 | 0.00 | 0.06 |
| Heat shock 70 kDa protein cognate 5 | DN49153_c0_g2 | 0.00 | 0.00 | 0.02 |
| Heat shock cognate 71 kDa protein | DN138673_c2_g1 | 0.86 | 1.33 | 119.08 |
| Heat shock protein 68 | DN132648_c1_g2 | 0.10 | 0.69 | 119.20 |
| Heat shock protein 70 A1 | DN138673_c2_g2 | 1.27 | 3.30 | 383.52 |
| Heat shock protein 70 A2 | DN138673_c2_g3 | 1.61 | 4.45 | 671.43 |
| Heat shock-related 70 kDa protein 2 | DN132648_c0_g1 | 0.00 | 0.00 | 10.43 |
| Major heat shock 70 kDa protein Ab | DN123600_c0_g1 | 0.32 | 0.98 | 10.04 |
| Heat shock protein 90 | DN147011_c0_g1 | 0.00 | 0.00 | 0.06 |
| Heat shock protein 90 | DN179523_c0_g1 | 0.00 | 0.00 | 0.08 |
| Heat shock protein 90 | DN121604_c0_g1 | 0.00 | 0.05 | 0.04 |
| Heat shock protein 90 | DN174468_c0_g1 | 0.00 | 0.00 | 0.25 |
| Heat shock protein 90 | DN12501_c0_g1 | 0.20 | 0.00 | 0.00 |
| Heat shock protein 90 | DN56672_c0_g1 | 0.00 | 0.00 | 0.21 |
| Heat shock protein 90 | DN56672_c0_g2 | 0.00 | 0.00 | 0.08 |
| Heat shock protein 90 | DN202295_c0_g1 | 0.60 | 0.00 | 0.00 |
| Heat shock protein 90 | DN242001_c0_g1 | 0.05 | 0.00 | 0.00 |
| Heat shock protein 90 | DN249470_c0_g1 | 0.00 | 0.00 | 0.02 |
| Heat shock protein 90 | DN267116_c0_g1 | 0.00 | 0.00 | 0.00 |
| Heat shock protein 90 | DN266724_c0_g1 | 0.03 | 0.00 | 0.05 |
| Heat shock protein 90 | DN264887_c0_g1 | 0.00 | 0.00 | 0.04 |
| Hsc70-interacting protein | DN7250_c0_g1 | 0.00 | 0.25 | 0.00 |
| Hsc70-interacting protein | DN135064_c0_g3 | 32.37 | 57.69 | 57.48 |
| Hsc70-interacting protein | DN202045_c0_g1 | 0.00 | 0.00 | 0.02 |
| Hsc70-interacting protein | DN295562_c0_g1 | 0.16 | 0.00 | 0.00 |
| Hsp70-Hsp90 organizing protein 1 | DN286792_c0_g1 | 0.23 | 0.00 | 0.00 |
| Hsp70-Hsp90 organizing protein 2 | DN109999_c0_g1 | 0.00 | 0.14 | 0.00 |
| Protein lethal(2)essential for life (hsp20) | DN124219_c0_g1 | 0.90 | 1.50 | 0.90 |
| Protein lethal(2)essential for life (hsp20) | DN128348_c0_g1 | 0.25 | 0.01 | 0.01 |
| Protein lethal(2)essential for life (hsp20) | DN130404_c0_g1 | 9.28 | 14.39 | 102.23 |
| Protein lethal(2)essential for life (hsp20) | DN130404_c0_g4 | 0.00 | 0.44 | 0.00 |
| Protein lethal(2)essential for life (hsp20) | DN131368_c0_g1 | 8.98 | 11.17 | 8.91 |
| Protein lethal(2)essential for life (hsp20) | DN133517_c2_g2 | 7.54 | 10.31 | 121.44 |
| Protein lethal(2)essential for life (hsp20) | DN134903_c4_g1 | 43.60 | 47.74 | 62.76 |
| Protein lethal(2)essential for life (hsp20) | DN135377_c1_g1 | 4.19 | 3.81 | 38.88 |
| Protein lethal(2)essential for life (hsp20) | DN135378_c2_g1 | 1.26 | 2.59 | 30.94 |
| Protein lethal(2)essential for life (hsp20) | DN137076_c3_g2 | 2.37 | 11.21 | 104.10 |
| Protein lethal(2)essential for life (hsp20) | DN137076_c3_g3 | 6.33 | 7.87 | 93.71 |
| Protein lethal(2)essential for life (hsp20) | DN133225_c0_g1 | 0.00 | 0.04 | 12.04 |
| Protein lethal(2)essential for life (hsp20) | DN139921_c2_g4 | 128.06 | 218.29 | 1227.09 |
| Protein lethal(2)essential for life (hsp20) | DN139952_c4_g1 | 4.19 | 2.73 | 169.07 |
| Protein lethal(2)essential for life (hsp20) | DN19123_c0_g1 | 0.04 | 0.00 | 0.01 |
| Protein lethal(2)essential for life (hsp20) | DN199991_c0_g1 | 0.00 | 0.13 | 0.00 |
| Protein lethal(2)essential for life (hsp20) | DN237735_c0_g1 | 0.00 | 0.16 | 0.00 |
| Protein lethal(2)essential for life (hsp20) | DN287457_c0_g1 | 0.00 | 0.18 | 0.00 |
| Protein lethal(2)essential for life (hsp20) | DN77607_c0_g1 | 0.00 | 0.21 | 0.00 |
| Protein lethal(2)essential for life (hsp20) | DN77607_c0_g2 | 0.00 | 0.00 | 0.00 |
| Protein lethal(2)essential for life (hsp20) | DN87858_c0_g1 | 0.05 | 0.01 | 0.00 |
| Protein lethal(2)essential for life (hsp20) | DN87858_c0_g2 | 0.03 | 0.05 | 0.00 |
| ***Grylloblatta bifratrilecta*** | | | | |
| Alpha-crystallin A chain | TR23551/c0_g1 | 0.00 | 0.00 | 0.00 |
| Heat shock 70 kDa protein cognate 3 | TR54732/c0_g1 | 6.40 | 2.39 | 2.08 |
| Heat shock 70 kDa protein cognate 4 | TR27372/c1_g1 | 0.00 | 0.00 | 0.00 |
| Heat shock 70 kDa protein cognate 4 | TR54307/c0_g1 | 37.43 | 26.67 | 40.40 |
| Heat shock 70 kDa protein cognate 5 | TR37786/c0_g1 | 0.00 | 0.00 | 0.00 |
| Heat shock 70 kDa protein 14 | TR11511/c16_g1 | 0.00 | 0.00 | 0.00 |
| Heat shock 70 kDa protein 4L | TR1170/c7_g1 | 0.00 | 0.00 | 0.00 |
| Heat shock cognate 71 kDa protein | TR50188/c0_g1 | 0.00 | 0.00 | 0.00 |
| Major heat shock 70 kDa protein Ab | TR26770/c0_g1 | 0.00 | 0.00 | 0.00 |
| Heat shock protein 70 A2 | TR32766/c8_g1 | 5.90 | 0.00 | 0.00 |
| Heat shock protein 90 | TR30799/c0_g1 | 0.00 | 0.00 | 0.00 |
| Heat shock protein HSP 90-alpha | TR24105/c19_g1 | 7.47 | 0.00 | 1.73 |
| Heat shock protein HSP 90-alpha | TR24105/c19_g2 | 20.28 | 2.38 | 8.24 |
| Hsc70-interacting protein | TR56327/c0_g1 | 0.00 | 0.99 | 0.00 |
| Hsp70-Hsp90 organizing protein 3 | TR39084/c0_g1 | 0.00 | 0.00 | 0.00 |
| Heat shock protein beta-1 | TR23037/c0_g1 | 0.00 | 0.00 | 0.00 |
| HSPB1-associated protein 1 | TR14130/c14_g2 | 0.00 | 0.00 | 0.00 |
| HSPB1-associated protein 1 | TR14130/c14_g3 | 0.00 | 0.00 | 0.00 |
| Protein lethal(2)essential for life (hsp20) | TR21324/c9_g1 | 0.00 | 0.00 | 0.00 |
| Protein lethal(2)essential for life (hsp20) | TR21324/c9_g2 | 0.00 | 0.00 | 0.00 |
| Protein lethal(2)essential for life (hsp20) | TR26349/c0_g1 | 0.00 | 6.48 | 0.00 |
| Protein lethal(2)essential for life (hsp20) | TR48919/c10_g1 | 0.00 | 0.00 | 2.80 |
| ***Grylloblatta marmoreus*** | | | | |
| Heat shock 70 kDa protein cognate 3 | TR4223\|c0_g1 | 329.48 | 190.42 | 193.43 |
| Heat shock 70 kDa protein cognate 4 | TR1383\|c0_g1 | 3.23 | 0.15 | 0.00 |
| Heat shock 70 kDa protein cognate 4 | TR629\|c0_g1 | 505.57 | 615.84 | 545.93 |
| Heat shock 70 kDa protein cognate 5 | TR11722\|c0_g1 | 60.81 | 50.60 | 47.00 |
| Heat shock 70 kDa protein 14 | TR12555\|c11_g1 | 4.46 | 4.95 | 6.90 |
| Heat shock 70 kDa protein 4L | TR700\|c6_g1 | 10.17 | 13.55 | 13.71 |
| Major heat shock 70 kDa protein Ab | TR10854\|c4_g1 | 33.17 | 32.34 | 19.49 |
| Heat shock protein 70 A2 | TR11655\|c2_g1 | 6.76 | 11.17 | 17.28 |
| Heat shock protein 90 | TR3833\|c0_g1 | 2.42 | 0.00 | 0.00 |
| Hsc70-interacting protein | TR1825\|c2_g1 | 26.90 | 26.10 | 29.43 |
| Heat shock protein beta-1 | TR4786\|c0_g1 | 12.44 | 10.55 | 3.95 |
| Heat shock protein beta-1 | TR9200\|c0_g1 | 4.31 | 0.00 | 0.00 |
| HSPB1-associated protein 1 | TR4925\|c0_g1 | 4.85 | 3.03 | 3.91 |
| Protein lethal(2)essential for life (hsp20) | TR4697\|c12_g1 | 100.14 | 182.67 | 60.27 |
| Protein lethal(2)essential for life (hsp20) | TR629\|c10_g1 | 3.11 | 1.47 | 1.62 |
| Protein lethal(2)essential for life (hsp20) | TR8671\|c4_g1 | 25.25 | 43.21 | 22.78 |
| ***Grylloblattella pravdini*** | | | | |
| Alpha-crystallin A chain | TR3602\|c20_g1 | 16.99 | 12.52 | 18.59 |
| Alpha-crystallin B chain | TR1331\|c0_g1 | 0.00 | 5.71 | 0.00 |
| Alpha-crystallin B chain | TR1331\|c0_g2 | 0.00 | 2.26 | 0.00 |
| Heat shock 70 kDa protein cognate 3 | TR15471\|c0_g1 | 183.57 | 120.92 | 158.50 |
| Heat shock 70 kDa protein cognate 4 | TR16681\|c0_g1 | 824.74 | 764.41 | 524.31 |
| Heat shock 70 kDa protein cognate 4 | TR18340\|c1_g1 | 0.00 | 3.27 | 0.00 |
| Heat shock 70 kDa protein cognate 4 | TR243\|c1_g1 | 1.95 | 0.58 | 0.42 |
| Heat shock 70 kDa protein cognate 5 | TR19269\|c0_g1 | 28.03 | 22.33 | 18.85 |
| Heat shock cognate 71 kDa protein | TR13851\|c0_g1 | 0.01 | 39.45 | 0.02 |
| Heat shock 70 kDa protein 14 | TR3687\|c20_g1 | 2.57 | 2.06 | 2.51 |
| Heat shock 70 kDa protein 4L | TR15168\|c0_g1 | 0.00 | 3.09 | 0.00 |
| Major heat shock 70 kDa protein Ab | TR9790\|c1_g2 | 3.63 | 2.39 | 3.00 |
| Heat shock protein 70 A2 | TR11245\|c4_g1 | 16.69 | 5.26 | 8.40 |
| Heat shock protein 90 | TR17964\|c0_g1 | 0.05 | 0.78 | 2.06 |
| Heat shock protein 90 | TR17964\|c0_g2 | 0.00 | 0.44 | 0.94 |
| Heat shock protein 90 | TR17964\|c1_g1 | 0.06 | 1.79 | 6.08 |
| Heat shock protein 90 | TR8308\|c0_g1 | 5.82 | 1.98 | 1.29 |
| Heat shock protein HSP 90-alpha | TR3674\|c1_g1 | 0.01 | 22.45 | 0.01 |
| Hsc70-interacting protein | TR1193\|c0_g1 | 0.02 | 5.44 | 0.00 |
| Hsc70-interacting protein | TR13529\|c1_g1 | 53.22 | 37.90 | 30.86 |
| Hsp70-Hsp90 organizing protein 1 | TR2586\|c0_g1 | 0.55 | 0.32 | 0.48 |
| Heat shock protein beta-1 | TR1025\|c0_g1 | 2.75 | 0.91 | 0.82 |
| Heat shock protein beta-1 | TR4199\|c4_g1 | 6.95 | 12.80 | 11.78 |
| HSPB1-associated protein 1 | TR2043\|c0_g1 | 5.99 | 8.97 | 10.37 |
| HSPB1-associated protein 1 | TR2043\|c0_g2 | 3.95 | 4.18 | 5.74 |
| Protein lethal(2)essential for life (hsp20) | TR18852\|c1_g1 | 115.73 | 72.23 | 53.61 |
| Protein lethal(2)essential for life (hsp20) | TR6610\|c0_g1 | 2.45 | 0.62 | 0.64 |
| Protein lethal(2)essential for life (hsp20) | TR7776\|c6_g1 | 108.02 | 87.22 | 77.36 |
| ***Galloisiana yezoensis*** | | | | |
| Alpha-crystallin B chain | TR7242\|c0_g1 | 49.50 | 0.00 | 0.00 |
| Heat shock 70 kDa protein cognate 3 | TR1100\|c0_g1 | 1808.50 | 1833.50 | 7346.00 |
| Heat shock 70 kDa protein cognate 3 | TR2099\|c2_g1 | 142.00 | 0.00 | 0.00 |
| Heat shock 70 kDa protein cognate 4 | TR15232\|c0_g1 | 47919.50 | 85839.50 | 71280.50 |
| Heat shock 70 kDa protein cognate 5 | TR4748\|c0_g1 | 3295.00 | 4175.00 | 5616.50 |
| Heat shock cognate 71 kDa protein | TR11271\|c0_g1 | 174.50 | 0.00 | 0.00 |
| Heat shock protein 70 B2 | TR6666\|c0_g1 | 171.50 | 97.50 | 37913.00 |
| Major heat shock 70 kDa protein Ab | TR17693\|c1_g1 | 343.00 | 393.00 | 430.00 |
| Heat shock 70 kDa protein 14 | TR7041\|c6_g1 | 241.50 | 361.00 | 224.00 |
| Heat shock 70 kDa protein 4L | TR2631\|c7_g1 | 100.00 | 107.00 | 281.50 |
| Heat shock protein HSP 90-alpha | TR14992\|c0_g1 | 613.50 | 0.00 | 0.00 |
| Hsc70-interacting protein | TR12369\|c0_g1 | 233.50 | 0.00 | 0.00 |
| Hsc70-interacting protein | TR8815\|c0_g1 | 1436.50 | 1702.00 | 2299.50 |
| Heat shock protein beta-1 | TR2342\|c0_g1 | 110.00 | 0.00 | 0.00 |
| HSPB1-associated protein 1 | TR7876\|c0_g1 | 112.50 | 126.00 | 113.00 |
| Protein lethal(2)essential for life (hsp20) | TR10714\|c0_g1 | 0.00 | 0.00 | 0.00 |
| Protein lethal(2)essential for life (hsp20) | TR10714\|c0_g2 | 1943.00 | 1942.50 | 2943.50 |
| Protein lethal(2)essential for life (hsp20) | TR14849\|c24_g1 | 836.50 | 736.50 | 1454.00 |
| ***Grylloblatta* sp. ‘North Cascades’ at Whitechuck Mountain** | | | | |
| Alpha-crystallin A chain | TR7330\|c0_g1 | 17.50 | 0.00 | 0.03 |
| Heat shock 70 kDa protein cognate 1 | TR10375\|c39_g1 | 2.36 | 2.13 | 1.90 |
| Heat shock 70 kDa protein cognate 3 | TR1338\|c0_g1 | 110.37 | 207.37 | 151.59 |
| Heat shock 70 kDa protein cognate 4 | TR211\|c0_g1 | 9.34 | 0.00 | 0.01 |
| Heat shock 70 kDa protein cognate 4 | TR5402\|c0_g1 | 799.55 | 1705.82 | 896.03 |
| Heat shock 70 kDa protein cognate 5 | TR1043\|c0_g1 | 47.82 | 62.44 | 53.18 |
| Heat shock cognate 71 kDa protein | TR62\|c0_g1 | 39.22 | 0.00 | 0.03 |
| Heat shock cognate protein HSP 90-beta | TR14721\|c0_g1 | 238.70 | 451.96 | 409.74 |
| Heat shock 70 kDa protein 13 | TR7827\|c0_g1 | 2.70 | 0.00 | 0.00 |
| Heat shock 70 kDa protein 14 | TR15650\|c1_g1 | 7.80 | 11.46 | 10.11 |
| Heat shock 70 kDa protein 2 | TR8624\|c0_g1 | 0.00 | 1.42 | 0.00 |
| Heat shock 70 kDa protein C | TR7080\|c0_g1 | 33.69 | 0.02 | 0.01 |
| Heat shock protein 70 A1 | TR10375\|c2_g1 | 5.47 | 7.42 | 7.21 |
| Heat shock protein 70 B2 | TR10375\|c15_g1 | 2.61 | 4.06 | 5.33 |
| Major heat shock 70 kDa protein Ab | TR6255\|c0_g1 | 48.07 | 39.34 | 30.36 |
| Heat shock protein 75 kDa, mitochondrial | TR15952\|c2_g1 | 4.24 | 0.00 | 0.00 |
| Heat shock protein HSP 90-alpha 1 | TR13210\|c0_g1 | 30.37 | 0.01 | 0.02 |
| Hsc70-interacting protein | TR8\|c0_g1 | 20.89 | 0.00 | 0.00 |
| Hsc70-interacting protein | TR833\|c10_g1 | 39.52 | 50.58 | 47.34 |
| Heat shock factor-binding protein 1 | TR5176\|c0_g1 | 6.40 | 0.00 | 0.00 |
| Hsp70-binding protein 1 | TR1321\|c0_g1 | 8.88 | 11.81 | 10.88 |
| Hsp70-binding protein 1 | TR7809\|c0_g1 | 3.56 | 0.00 | 0.00 |
| HSPB1-associated protein 1 | TR12066\|c7_g1 | 3.88 | 4.59 | 4.19 |
| Protein lethal(2)essential for life (hsp20) | TR14915\|c0_g1 | 190.56 | 119.72 | 107.74 |
| Protein lethal(2)essential for life (hsp20) | TR15768\|c5_g1 | 102.72 | 115.04 | 94.27 |
| ***Grylloblatta* sp. “Lillburn Cave”** | | | | |
| Heat shock 70 kDa protein cognate 3 | TR10159\|c0_g1 | 187.99 | 220.58 | 162.64 |
| Heat shock 70 kDa protein cognate 4 | TR3425\|c0_g1 | 1063.30 | 981.52 | 1359.58 |
| Heat shock 70 kDa protein cognate 5 | TR8756\|c0_g1 | 40.15 | 38.12 | 43.53 |
| Heat shock cognate protein HSP 90-beta | TR2458\|c0_g1 | 400.96 | 481.86 | 458.89 |
| Heat shock 70 kDa protein 14 | TR8020\|c10_g1 | 3.78 | 3.52 | 3.51 |
| Heat shock 70 kDa protein 4L | TR3362\|c0_g1 | 13.42 | 13.36 | 14.46 |
| Major heat shock 70 kDa protein Ab | TR13950\|c2_g1 | 23.55 | 23.71 | 26.60 |
| Heat shock protein 70 A2 | TR3090\|c0_g1 | 11.56 | 29.68 | 49.63 |
| Heat shock protein 75 kDa, mitochondrial | TR9251\|c0_g1 | 2.05 | 2.07 | 2.69 |
| Hsc70-interacting protein | TR1863\|c0_g1 | 34.38 | 40.64 | 31.99 |
| Heat shock protein beta-1 | TR11506\|c0_g1 | 16.92 | 17.69 | 26.27 |
| Hsp70-binding protein 1 | TR3460\|c12_g1 | 6.76 | 4.20 | 6.17 |
| Hsp70-binding protein 1 | TR3460\|c18_g1 | 3.78 | 2.63 | 4.13 |
| HSPB1-associated protein 1 homolog | TR2172\|c5_g1 | 3.90 | 1.83 | 3.68 |
| Protein lethal(2)essential for life (hsp20) | TR11643\|c9_g1 | 3.40 | 1.88 | 5.32 |
| Protein lethal(2)essential for life (hsp20) | TR1210\|c0_g1 | 77.70 | 89.01 | 147.51 |
| Protein lethal(2)essential for life (hsp20) | TR7235\|c11_g1 | 110.36 | 161.55 | 146.82 |

**Supplementary Table S15**. Gene families found to be under selection using the FUBAR branch-site test implemented in FUSTR. Gene families are grouped by biological function. Gene families with orthologs present in Mantophasmatodea and Grylloblattodea are labeled as ‘both’ taxa represented.

| **Gene Family #** | **# of sites under selection** | **BLAST result** | **Annotation** | **Taxon Represented** |
| --- | --- | --- | --- | --- |
| **Stress/Immune Reponse, DNA Damage, Proteolysis, Apoptosis** | | | | |
| 13544 | 3 | Beta-catenin-like protein 1 | apoptotic process | Both |
| 17661 | 3 | protein croquemort-like | apoptotic process; phagocytosis; immune response | Both |
| 14748 | 9 | E3 SUMO-protein ligase NSE2-like | cell division; cellular senescence | Grylloblattodea |
| 12494 | 7 | CD9 antigen-like | cell surface receptor signaling pathway; response to water deprivation | Both |
| 5518 | 3 | superoxide dismutase [Mn], mitochondrial | cellular response to oxidative stress; defense response | Both |
| 6021 | 6 | 46 kDa FK506-binding nuclear protein | DNA binding; regulates protein folding | Both |
| 2484 | 3 | Barrier-to-autointegration factor-like | DNA binding; response to virus | Both |
| 3272 | 3 | growth arrest and DNA-damage inducible protein GADD45 | DNA damage response | Both |
| 4064 | 4 | COP9 signalosome complex subunit 6 | DNA damage response | Both |
| 9548 | 5 | attacin-B/A like / sarcotoxin-2A-like | innate immune response; response to hyperoxia | Mantophasmatodea |
| 6662 | 9 | baculoviral IAP repeat-containing protein 5 | negative regulation of apoptotic process; transcription | Both |
| 6961 | 3 | Pathogenesis-related protein 5-like | pathogen resistance | Both |
| 10822 | 3 | hsp70-binding protein 1 | positive regulation of proteasomal ubiquitin-dependent protein catabolic process | Both |
| 11236 | 6 | U4/U6.U5 tri snRNP associated protein 2 | protein deubiquitination | Both |
| 4680 | 4 | ATP-dependent Clp protease proteolytic subunit | proteolysis | Both |
| 10278 | 3 | serine protease gd-like / serine proteinase stubble-like | proteolysis | Both |
| 16536 | 4 | serine proteinase stubble-like isoform | proteolysis | Grylloblattodea |
| 6186 | 5 | trypsin-1/trypsin 3A1 | proteolysis; digestion | Mantophasmatodea |
| 12624 | 4 | lipophorin receptor / low-density lipoprotein receptor 1-like | proteolysis; lipid metabolic process; response to caloric restriction | Both |
| 16441 | 4 | serine protease inhibitor 28Dc-like | proteolysis; multicellular organism development | Both |
| 6608 | 4 | E3 ubiquitin-protein ligase RNF146 | proteolysis; ubiquitin-dependent protein catabolic process | Both |
| 6597 | 9 | serine proteinase stubble-like | proteolysis; wing disc development | Both |
| 7570 | 3 | NEDD8-activating enzyme E1 regulatory subunit | regulation of apoptotic process; signal transduction | Both |
| 6089 | 3 | Segmentation protein cap’n’collar | response to oxidative stress; determination of adult lifespan; development; DNA binding | Both |
| 4754 | 3 | activating transcription factor of chaperone | response to unfolded protein | Both |
| 5414 | 3 | X-box-binding protein 1 | response to unfolded protein | Both |
| 5797 | 3 | 26S proteasome non-ATPase regulatory subunit 4 | response to unfolded protein; proteasome assembly | Both |
| 5999 | 3 | hspD1 60 kDa heat shock protein, mitochondrial | response to unfolded protein; stress response | Both |
| 10737 | 3 | F-box only protein | SCF-dependent proteasomal ubiquitin-dependent protein catabolic process | Both |
| 9829 | 3 | CDK-activating kinase assembly factor MAT1 | transcription; negative regulation of apoptotic process | Both |
| 14646 | 5 | saxiphilin-like | xenobiotic detoxification | Grylloblattodea |
| **Oxygen Reduction and Cellular Respiration** | | | | |
| 3408 | 3 | thioredoxin domain containing protein15 | cell redox homeostasis | Both |
| 3049 | 3 | FAD-linked sulfhydryl-oxidase ALR | oxidoreductase activity | Both |
| 3226 | 5 | NADH quinone oxidoreductase subunit | oxidoreductase activity | Both |
| 5943 | 3 | sarcosine dehydrogenase, mitochondrial | oxidoreductase activity | Both |
| 8809 | 4 | dehydrogenase/reductase SDR family member 7 | oxidoreductase activity | Both |
| 18739 | 4 | cytochrome P450 9e2-like | oxidoreductase activity | Grylloblattodea |
| 3468 | 3 | NADH-dehydrogenase ubiquinone-1 alpha subcomplex subunit-11 | oxidoreductase activity; respiration | Both |
| 3580 | 3 | PET100 homolog | respiration | Both |
| 3673 | 3 | L-lactate dehydrogenase | respiration | Both |
| 4014 | 3 | glycerol-3-phosphate dehydrogenase[NAD(+)], cytoplasmic | respiration | Both |
| 19772 | 3 | DNA polymerase subunit gamma-2, mitochondrial | respiration; DNA repair | Both |
| 5310 | 3 | Ubiquinol-cytochrome-c reductase complex assembly factor 1 | respiration; mitochondrial translation | Both |
| 8682 | 6 | surfeit locus protein 1 | respiration; oxidation-reduction process | Both |
| **Metabolism and Development** | | | | |
| 11440 | 3 | putative aminopeptidase W07G4.4 | aminopeptidase activity | Both |
| 11086 | 3 | ABC transporter G family member 23 | ATP binding | Both |
| 3027 | 4 | ATP synthase subunit-g | ATP biosynthetic process | Both |
| 5917 | 4 | galectin-8-like/galectin-4-like isoform | carbohydrate binding | Both |
| 6684 | 5 | flocculation protein FLO11/probable GPI-anchored adhesin-like protein PGA55 | cell adhesion | Both |
| 15585 | 3 | uncharacterized protein / peritrophin-like | chitin binding | Grylloblattodea |
| 5186 | 3 | chitooligosaccharidolytic beta-N-acetylglucosaminidase | chitin catabolic process | Both |
| 4563 | 4 | protein obstructor-E-like isoform X1 | chitin metabolic process | Both |
| 2596 | 3 | matrix remodeling associated protein | extracellular matrix | Both |
| 6140 | 3 | aldose-1-epimerase | glucose metabolic process | Both |
| 17227 | 32 | apolipoprotein D-like | glucose metabolic process; response to reactive oxygen species | Both |
| 6411 | 3 | hydroxyacylglutathione hydrolase, mitochondrial isoform | glutathione biosynthetic process | Both |
| 3880 | 6 | persulfide dioxygenase ETHE1, mitochondrial | glutathione metabolic process | Both |
| 4208 | 4 | putative GPI-anchor transamidase | glycolipid biosynthesis | Both |
| 11108 | 3 | protein Peter pan / suppressor of SWI4 1 | larval development; oogenesis | Both |
| 12715 | 3 | protein ABHD4 | lipid homeostasis | Both |
| 12707 | 5 | microsomal triglyceride transfer protein large subunit | lipid metabolic process | Both |
| 11579 | 4 | prosaposin | lipid transport | Both |
| 3916 | 3 | protein yellow-like | melanin biosynthetic process | Both |
| 4704 | 5 | esterase FE4 | methyl indole-3-acetate esterase activity | Mantophasmatodea |
| 14538 | 5 | esterase E4-like | methyl indole-3-acetate esterase activity | Both |
| 6780 | 4 | Phytanoyl-CoA dioxygenase, peroxisomal-like | methyl-branched fatty acid metabolic process; protein targeting to peroxisome | Both |
| 2393 | 4 | TPPP Protein | microtubule organization | Both |
| 2460 | 3 | Brick-1 B | microtubule organization | Both |
| 3177 | 6 | Dynactin subunit-3 like | microtubule organization | Both |
| 6336 | 3 | regulator of microtubule dynamics protein 1 | microtubule organization | Both |
| 12947 | 3 | serine/threonine-protein kinase MARK2-like | microtubule organization; autophagy of mitochondrion | Both |
| 6114 | 3 | Protein slowmo | multicellular organism development; phospholipid transport | Both |
| 20196 | 6 | deoxycytidylate deaminase | pyrimidine nucleotide metabolic process | Grylloblattodea |
| 6174 | 4 | ragulator complex protein LAMTOR2 | regulation of cell growth; cellular response to amino acids | Both |
| 6047 | 4 | 2-oxoisovalerate dehydrogenase subunit beta, mitochondrial | response to nutrient | Both |
| **DNA Replication and Protein Synthesis** | | | | |
| 6855 | 3 | protein RTF2 homolog | DNA replication | Both |
| 6260 | 4 | protein dpy-30 homolog | histone H3-K4 methylation | Both |
| 7583 | 4 | probable cysteine desulfurase, mitochondrial | iron-sulfur cluster assembly; removal of elemental sulfur | Both |
| 3788 | 4 | 28S ribosomal protein-S31 | mitochondrial translation | Both |
| 4287 | 3 | 39S ribosomal protein L38, mitochondrial | mitochondrial translation | Both |
| 4357 | 3 | probable 28S ribosomal protein S26, mitochondrial | mitochondrial translation | Both |
| 5385 | 3 | probable 39S ribosomal protein L23, mitochondrial | mitochondrial translation | Both |
| 6427 | 4 | 28S ribosomal protein S7, Mitochondrial | mitochondrial translation | Both |
| 7372 | 3 | 28S ribosomal protein S28, mitochondrial | mitochondrial translation | Both |
| 16105 | 5 | growth arrest and DNA damage-inducible proteins-interacting protein 1 | mitochondrial translation | Grylloblattodea |
| 4415 | 3 | Pre-mRNA-splicing factor CWC25 homolog | mRNA splicing | Both |
| 6199 | 3 | protein RER1 | protein retention in ER lumen | Both |
| 3840 | 4 | secretory carrier-associated membrane protein 1 | protein transport | Both |
| 15921 | 3 | stromal membrane-associate protein/DNA primase small subunit | regulation of clathrin-dependent endocytosis; replication | Both |
| 15542 | 4 | DNA primase large subunit | replication | Both |
| 2899 | 3 | 60S ribosomal protein L 24 | ribosomal large subunit assembly | Both |
| 20931 | 3 | ribosome biogenesis protein BRX1 | ribosomal large subunit assembly | Grylloblattodea |
| 6802 | 3 | uncharacterized protein C1orf131 | RNA binding | Both |
| 7554 | 3 | THUMP domain-containing protein 1 | RNA binding | Both |
| 19091 | 3 | ribosome biogenesis protein TSR3 | rRNA modification | Both |
| 3509 | 3 | RRP15-like protein | rRNA processing | Both |
| 4946 | 4 | surfeit locus protein 6 homolog | rRNA processing | Both |
| 5753 | 3 | WD repeat containing protein 46 | rRNA processing | Both |
| 6248 | 3 | ribosome biogenesis protein BMS1 homolog | rRNA processing | Both |
| 7026 | 3 | nucleolar complex protein 4 homolog A/B | rRNA processing | Both |
| 11489 | 4 | probable ATP-dependent RNA helicase DDX27 | rRNA processing | Both |
| 4387 | 3 | periodic tryptophan protein 1 homolog | rRNA processing; transcription | Both |
| 7177 | 3 | U3 small nucleolar RNA-associated protein 15 homolog | rRNA processing; transcription | Both |
| 6746 | 3 | NKAP family protein CG6066 | transcription | Both |
| 13833 | 5 | zinc finger protein | transcription regulation | Grylloblattodea |
| 13900 | 7 | zinc finger protein | transcription regulation | Grylloblattodea |
| 14030 | 5 | BTB/POZ domain containing protein | transcription regulation | Grylloblattodea |
| 14299 | 3 | zinc finger protein | transcription regulation | Grylloblattodea |
| 14379 | 4 | zinc finger protein | transcription regulation | Grylloblattodea |
| 33614 | 4 | zinc finger MYM-type protein 1 | transcription | Grylloblattodea |
| 2622 | 5 | Coiled-coil domain containing protein | transcription; gene regulation | Both |
| 2748 | 3 | prefoldin subunit-5 | transcription; protein folding | Both |
| 4906 | 3 | ankyrin repeat domain containing protein / various | transcription; response to hypoxia | Mantophasmatodea |
| 11149 | 3 | 28S ribosomal protein S30, mitochondrial | translation | Both |
| 10379 | 3 | Methyltransferase-like protein 2 | tRNA methylation | Both |
| 20504 | 3 | la protein homolog | tRNA processing | Grylloblattodea |
| 4657 | 3 | protein SMG5 | ubiquitin protein ligase binding; mRNA degradation | Both |
| 2555 | 3 | Predicted myelin and lymphocyte protein | vesicular transport of proteins | Both |
| **Olfaction and Nervous System** | | | | |
| 16321 | 3 | Slit-like protein / leucine rich repeat containing protein/ various | axon guidance | Grylloblattodea |
| 13268 | 3 | Prohormone-2 | extracellular signalling | Both |
| 16838 | 10 | general odorant binding protein /acidic ribosomal protein /various | olfaction | Grylloblattodea |
| 18670 | 10 | general odorant binding protein | olfaction | Grylloblattodea |
| 20209 | 3 | general odorant-binding protein | olfaction | Grylloblattodea |
| 11293 | 3 | syntaxin-1A isoform | positive regulation of neurotransmitter secretion | Both |
| 13541 | 3 | rap1 GTPase-activating protein 1 | signal transduction | Both |
| **Unknown Proteins** | | | | |
| 117 | 5 | Uncharacterized protein | Unknown protein | Both |
| 3007 | 3 | No significant similarity | Unknown protein | Both |
| 3929 | 3 | INCONCLUSIVE | Unknown protein | Mantophasmatodea |
| 8116 | 3 | uncharacterized protein | Unknown protein | Both |
| 8404 | 14 | No significant similarity found / various | Unknown protein | Mantophasmatodea |
| 8570 | 3 | tetratricopeptide repeat protein 4 | Unknown protein | Both |
| 9100 | 9 | no significant similarity found | Unknown protein | Mantophasmatodea |
| 9533 | 3 | no significant similarity found | Unknown protein | Mantophasmatodea |
| 9840 | 5 | hypothetical protein B7P43 G02634 | Unknown protein | Both |
| 11313 | 3 | uncharacterized protein | Unknown protein | Both |
| 11632 | 7 | hypothetical protein/mixed results | Unknown protein | Both |
| 14110 | 25 | uncharacterized protein/various | Unknown protein | Both |
| 14483 | 13 | uncharacterized protein | Unknown protein | Both |
| 14542 | 6 | uncharacterized protein | Unknown protein | Grylloblattodea |
| 14634 | 4 | uncharacterized protein/various | Unknown protein | Grylloblattodea |
| 15687 | 8 | no significant similarity (few results poor alignment scores) | Unknown protein | Grylloblattodea |
| 15711 | 7 | no significant similarity (few results poor alignment scores) | Unknown protein | Grylloblattodea |
| 15928 | 13 | no significant similarity found | Unknown protein | Grylloblattodea |
| 15976 | 32 | various | Unknown protein | Grylloblattodea |
| 16437 | 4 | no significant similarity found | Unknown protein | Grylloblattodea |
| 16692 | 9 | prion-likel(QN=/N-rich) domain-bearing protein 25 / various | Unknown protein | Grylloblattodea |
| 18269 | 3 | ARL14 effector protein-like | Unknown protein | Grylloblattodea |
| 18310 | 6 | no significant similarity found | Unknown protein | Grylloblattodea |
| 18774 | 3 | Uncharacterized / hypothetical protein | Unknown protein | Both |
| 18822 | 5 | uncharacterized protein | Unknown protein | Grylloblattodea |
| 20211 | 3 | various | Unknown protein | Grylloblattodea |
| 20781 | 5 | armadillo repeat-containing protein 7 | Unknown protein | Grylloblattodea |
| 20853 | 3 | various | Unknown protein | Both |
| 25546 | 5 | Various / no significant similarity | Unknown protein | Mantophasmatodea |


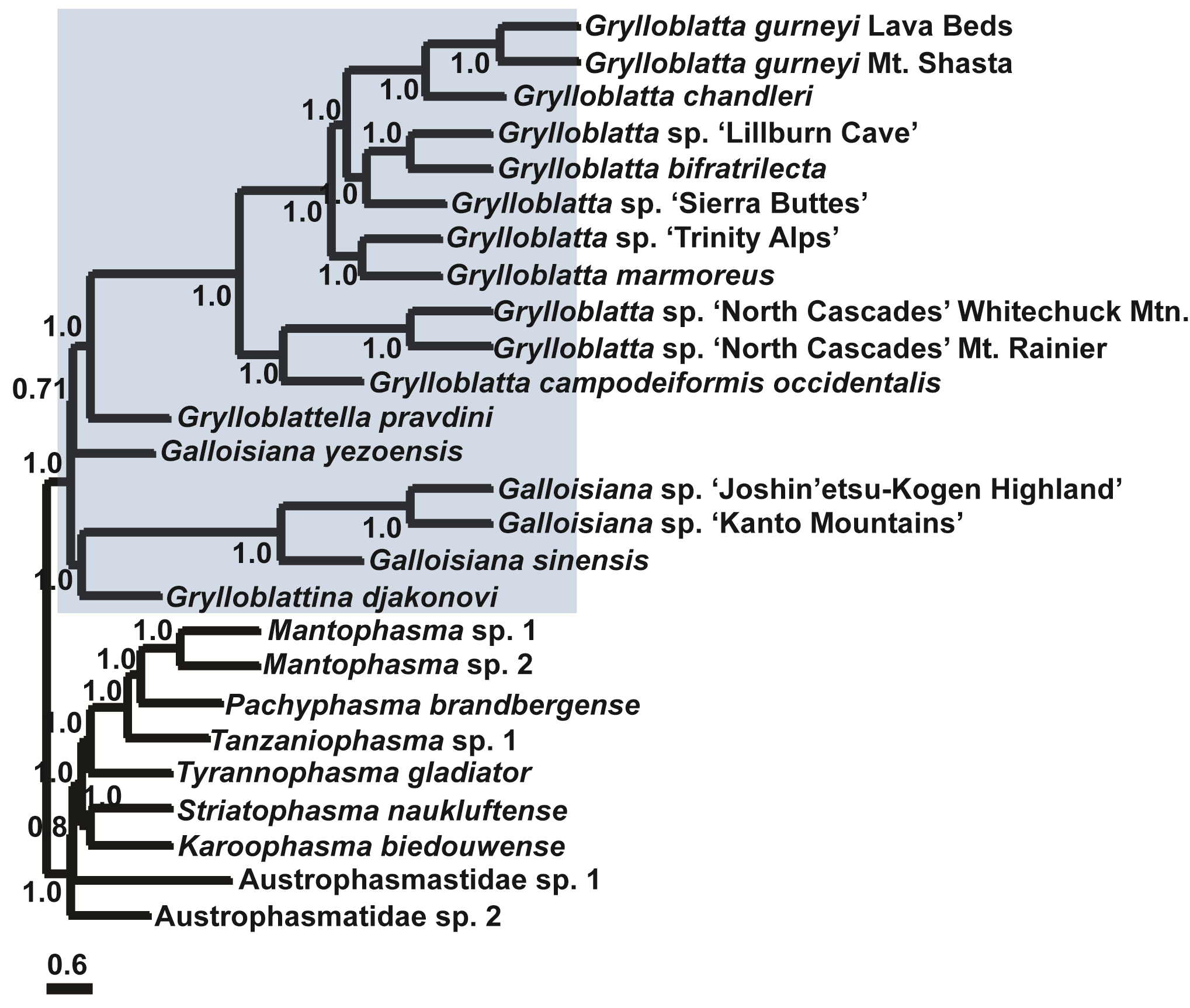


**Supplementary Figure S1**. Multi-species coalescent trees inferred from astral-II showing the phylogenetic relationships of Mantophasmatodea and Grylloblattodea based on dataset 2 from hamstr, with 809 genes. Numbers at nodes indicate local posterior probability, and branch lengths are represented in coalescent units.


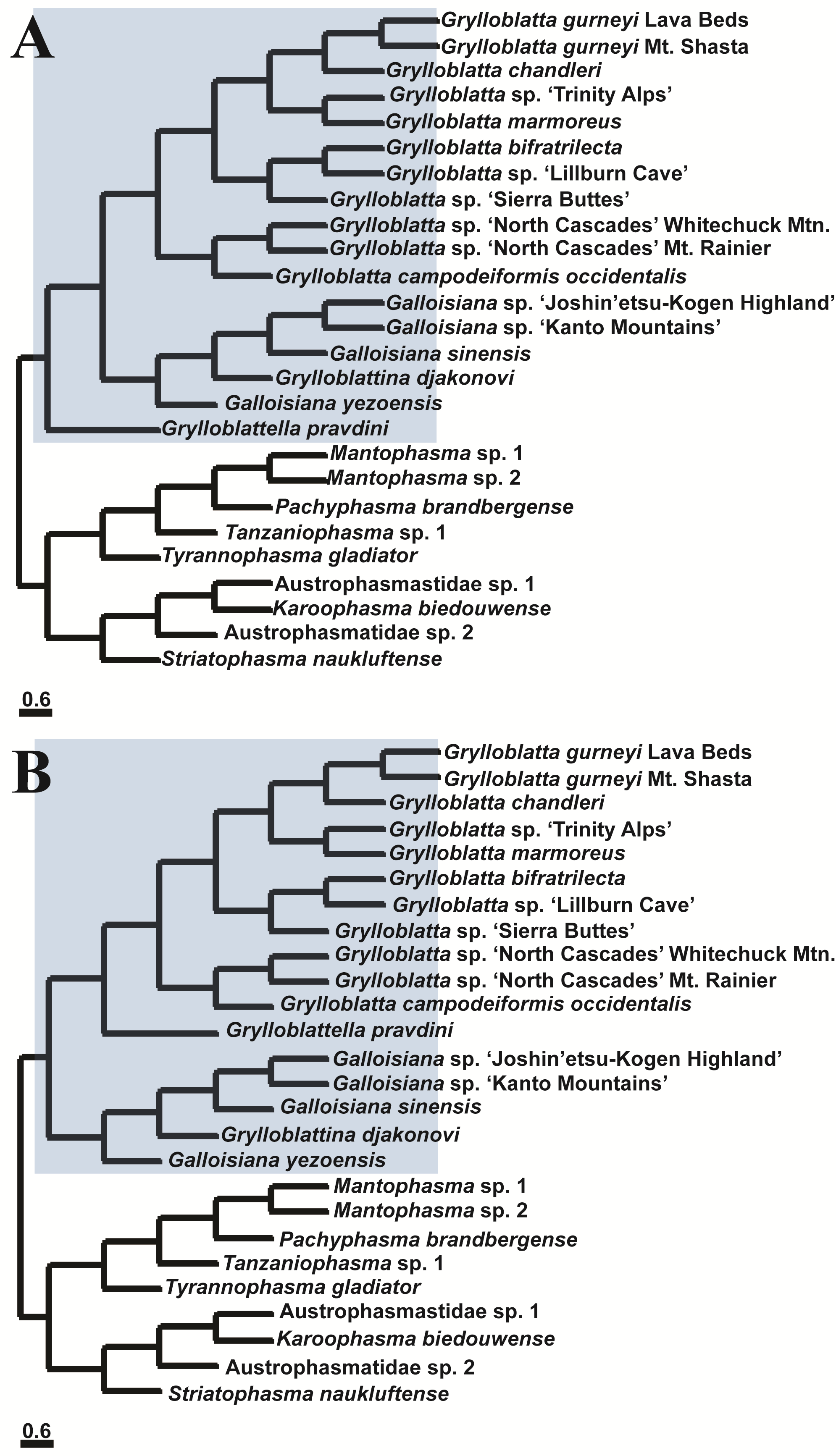


**Supplementary Figure S2**. The maximum likelihood tree inferred from the concatenated amino-acid dataset of 3,022 genes. ML tree A) received the best log-likelihood score eight times across 50 independent tree searches, while B) received the best log-likelihood score three times. Branch lengths in both trees are unscaled.
